# Separation of xylene isomers in an adaptable metal-organic framework

**DOI:** 10.1126/sciadv.aeg1691

**Published:** 2026-08-14

**Authors:** Jiaqi Li, Rundao Chen, Lidong Guo, Haoran Sun, Jialei Yan, Ying Liu, Fuxing Shen, Qiwei Yang, Zhiguo Zhang, Qilong Ren, Dan Zhao, Zongbi Bao

**Affiliations:** ^1^ Key Laboratory of Biomass Chemical Engineering of Ministry of Education, College of Chemical and Biological Engineering, Zhejiang University, 310058 Hangzhou, Zhejiang, China.; ^2^ Department of Chemical and Biomolecular Engineering, National University of Singapore, 4 Engineering Drive 4, Singapore 117585, Singapore.; ^3^ Institute of Zhejiang University-Quzhou, 324000 Quzhou, Zhejiang, China.

## Abstract

Efficient separation of xylene isomers by adsorption remains challenging due to insufficient selectivity arising from their molecular similarity. We report an adaptable metal-organic framework, Ni(BIC)_2_ [1-*H*-benzimidazole-5-carboxylic acid (HBIC)], that exhibits high selectivity for *para*-xylene over other xylene isomers. Ni(BIC)_2_ features a guest-adaptive one-dimensional channel that precisely matches the molecular projection dimensions of *para*-xylene, enabling sieving from a quaternary mixture of xylene isomers. The structural transformation of Ni(BIC)_2_ upon guest adsorption substantially widens the disparity in binding energies between *para*-xylene and its counterparts. Weakly adsorbed components are converted into nonadsorbed entities, leading to temperature-independent *para*-xylene uptake while excluding *meta*-xylene and *ortho*-xylene. Breakthrough experiments demonstrate remarkable dynamic selectivity, delivering high-purity *para*-xylene within a single fixed-bed adsorption process. Simulated moving bed (SMB) process analysis demonstrates that Ni(BIC)_2_ delivers a 3.2-fold increase in production efficiency relative to conventional zeolite under milder operating conditions.

## INTRODUCTION

*Para*-xylene (PX) is an essential raw material for polyester production (*1*). PX is typically obtained from the catalytic reforming of naphtha, which produces a mixture of C8 aromatic compounds. This mixture mainly consists of PX along with *meta*-xylene (MX), *ortho*-xylene (OX), and ethylbenzene (EB) (*2*). The separation and purification of PX from this blend of highly similar isomers represent a substantial but technologically challenging process (*3*). Separating all four isomers through distillation is practically unfeasible due to their close boiling points (table S1) (*4*). While PX could ideally be isolated by fractional crystallization, this method requires considerable energy input to maintain cryogenic temperatures for PX crystallization (*5*). Now, more than 75% of industrial PX production relies on simulated moving bed (SMB) technology using FAU (Faujasite) zeolites as adsorbents at high temperatures ranging from 393 to 523 K (*6*, *7*). While elevated temperatures are necessary to achieve sufficient mass-transfer rates for bulky adsorbates such as xylene, they inevitably reduce the selectivity and adsorption capacity of zeolites (*8*, *9*). This trade-off between selectivity/capacity and temperature arises from the lack of structural tunability and flexibility in zeolite frameworks (*10*). Furthermore, the strong binding interactions between xylenes and zeolite lead to problematic coadsorption and reduced working capacities. Therefore, designing advanced adsorbents is crucial for developing more energy-efficient separation processes for xylene mixtures.

Solid adsorbents, such as modified zeolites (*11**–**13*), organic cages (*14*), metal complexes (*15*–*17*), and molecular crystals (*18*, *19*), have garnered extensive research interest for their potential in the efficient separation of xylene isomers. Among these, metal-organic frameworks (MOFs) stand out as a promising class of materials due to their exceptional structural versatility and flexibility (*20*, *21*). This includes features like reversible “breathing” (*22*, *23*), “pore opening” (*24*, *25*), “shape memory” (*26*), and dynamic “gating flexibility” (*10*, *27*, *28*). These characteristics enable precise molecular recognition of many industrially relevant mixtures (*29*–*33*) and the effective separation of xylene isomers (*34*–*36*). Despite these advances, achieving complete PX sieving from its C8 aromatic counterparts at high temperatures remains an unsolved challenge.

**Fig. 1.**
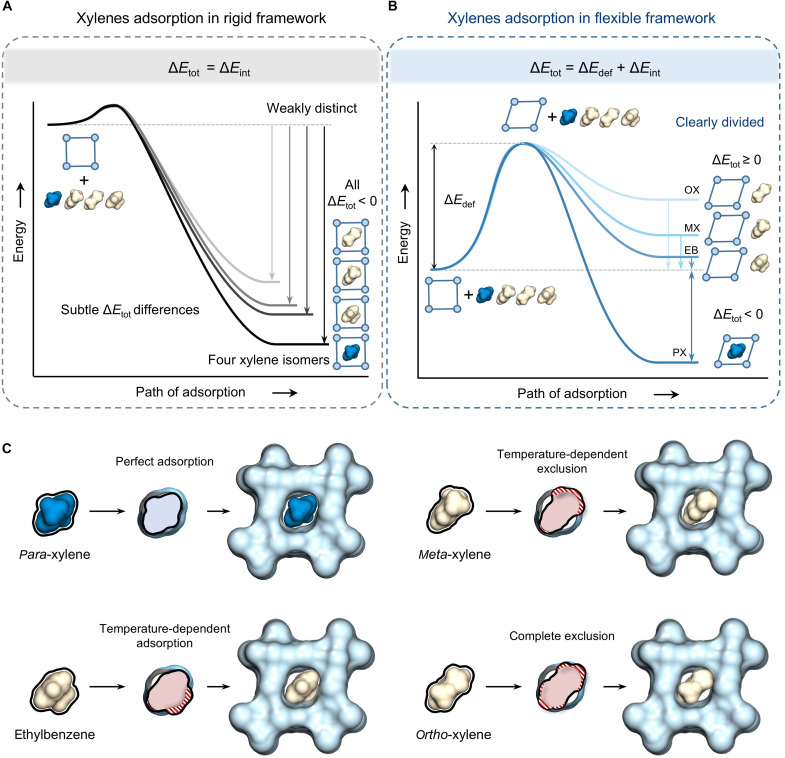
Mechanistic illustrations for the efficient separation of xylene isomers. (**A** and **B**) Schematic representation of contrast enhancement strategy used to amplify the difference of Δ*E*_tot_ between typical (A) rigid and (B) flexible frameworks, where Δ*E*_int_ is the interaction energy between guest and framework, Δ*E*_def_ is the framework deformation energy, and Δ*E*_tot_ is the total adsorption energy. These schematics illustrate an idealized and qualitative description of the contrast enhancement mechanism, rather than quantitative calculated energy values. (**C**) Illustration of shape matching between xylene isomers and the transformed channel of Ni(BIC)_2_. Variations in shape matching between isomers and channels lead to corresponding differences in interaction energy, ultimately resulting in distinct adsorption behaviors. PX shows the best shape fitness and readily enters the pore, whereas poorly matched isomers show limited accessibility, hindered by the deformation energy barrier.

We propose an approach that leverages differences in adsorption enthalpy, similar to how activation energy differences are exploited in chemical reactions. For a rigid framework, the total adsorption enthalpy corresponds to the interaction energy (Fig. 1A). However, within a flexible framework, the total adsorption enthalpy encompasses both the interaction energy and the framework deformation energy (Fig. 1B) (*26*, *37*, *38*). Thus, when the interaction energy between guest molecules and the framework is generally high, a portion of this interaction energy can be counterbalanced by the deformation energy of the flexible framework. This concept is demonstrated in an adaptable MOF, termed Ni(BIC)_2_ [1-*H*-benzimidazole-5-carboxylic acid (HBIC)] (*39*), which features one-dimensional channels capable of guest-adaptive lattice transformations (Fig. 1C and fig. S1). By imposing a thermodynamic penalty on interaction through framework deformation, weakly adsorbed components tend to behave as excluded components as they are less likely to surmount the energy barrier necessary for structural transformation. We term this mechanism “contrast enhancement,” inspired by analogous techniques in photography where image details are amplified by reducing the intensities of bright pixels, serving as a powerful demonstration of pore-opening principles (*37*) for a multicomponent separation task. In Ni(BIC)_2_, such an induced-fit mechanism akin to enzyme-substrate binding facilitates exceptional PX selectivity, thereby overcoming the constraints of previous adsorbents.

## RESULTS AND DISCUSSION

**Fig. 2.**
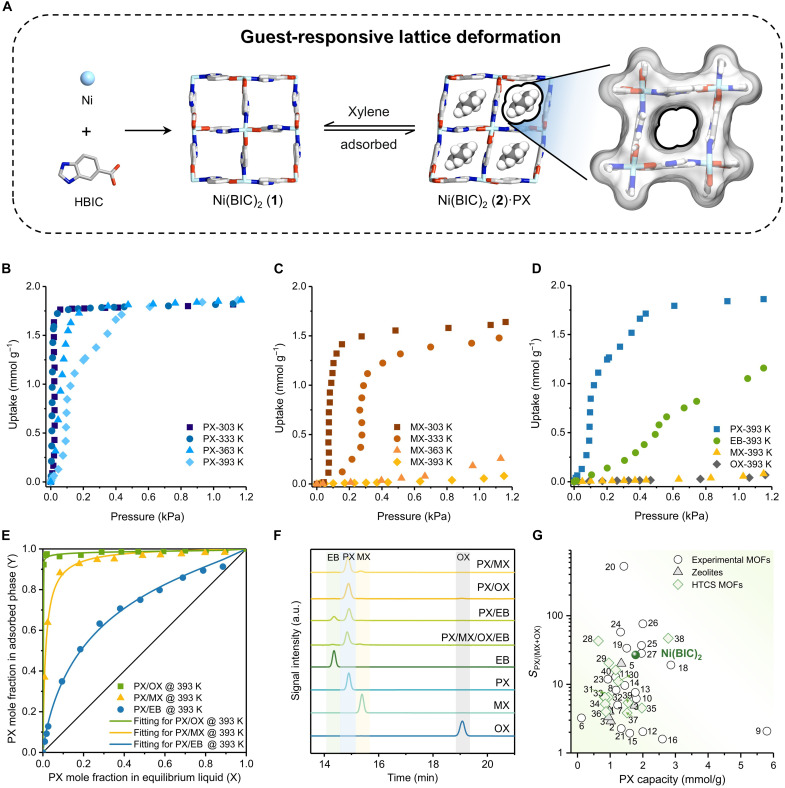
Crystallographic structure transformation and adsorption behavior of xylene isomers on Ni(BIC)_2_. (**A**) Crystal structure of Ni(BIC)_2_ and the single-crystal–to–single-crystal (SCSC) transition of Ni(BIC)_2_ during xylene adsorption (color code: Ni, blue; C, gray; O, red; N, dark blue; H, white). The right figure is the Connolly surface of Ni(BIC)_2_ on the *ab* plane with a probe radius of 1 Å. (**B**) Single-component adsorption isotherms of PX vapor on Ni(BIC)_2_ at 303 to 393 K. (**C**) Single-component adsorption isotherms of MX on Ni(BIC)_2_ at 303 to 393 K. (**D**) Comparison of single-component vapor adsorption of xylene isomers at 393 K. (**E**) Adsorption equilibrium of binary solutions of PX/OX, PX/MX, and PX/EB on Ni(BIC)_2_ at 393 K, with a total capacity in a range of 1.70 to 1.76 mmol g^−1^. (**F**) Gas chromatograms of adsorbed phase on Ni(BIC)_2_ equilibrated with equimolar binary liquid mixtures of PX/MX, PX/OX, PX/EB, and quaternary mixture of PX/MX/OX/EB with a molar ratio of 22/50/22/6 at 393 K, as well as standard sample of EB, PX, MX, and OX, respectively. a.u., arbitrary units. (**G**) Comparison of S_PX/(MX+OX)_-PX capacity performance on zeolites, experimental MOFs, and HTCS MOFs. Data are collected from reported adsorbents (table S3), with the highest reported selectivity for each material adopted for comparison. Material names are numbered as listed in table S3.

Green obelisk-like crystals of Ni(BIC)_2_ (where BIC stands for Benzimidazole-5-carboxylic acid) were synthesized by solvothermal reaction between nickel(II) nitrate hexahydrate and HBIC under autogenous pressure (fig. S2). Single-crystal x-ray diffraction (SCXRD) revealed that Ni(BIC)_2_ crystallizes in the tetragonal P4_3_2_1_2 space group, with an asymmetric unit containing two BIC linkers and one Ni^2+^ ion (table S2). Each Ni^2+^ is octahedrally coordinated by two pairs of bidentate chelating carboxylate oxygen atoms from two HBIC linkers and two nitrogen atoms from another two HBIC linkers, forming an infinite helical chain propagating along the *c* axis. Three of these helical polymer chains twist together, creating tetragonal channels with dimensions of ∼5 Å by 5 Å. These channels are lined with benzimidazole groups from the HBIC linkers and share edges with each other to construct a three-dimensional supramolecular porous network (fig. S3). Topologically, the framework can be represented as a unimodal 4-connected network with **dia** topology, having a vertex symbol of {66} (fig. S4). Upon exposure of C8 aromatic guest molecules except OX, Ni(BIC)_2_ underwent a single-crystal–to–single-crystal (SCSC) transformation from a P4_3_2_1_2 tetragonal phase [denoted as Ni(BIC)_2_ (**1**)] to a C222_1_ orthorhombic phase [denoted as Ni(BIC)_2_ (**2**)] (Fig. 2A and table S2). Powder x-ray diffraction (PXRD) patterns confirmed the phase purity of the as-synthesized Ni(BIC)_2_, with the experimental pattern showing excellent agreement with the simulated pattern calculated from the single-crystal data (fig. S5). Variable temperature PXRD patterns and thermogravimetric analysis verified the remarkable thermal stability of Ni(BIC)_2_ up to 673 K under nitrogen atmosphere (fig. S6). The Brunauer-Emmett-Teller surface area of Ni(BIC)_2_ was estimated to be 319 m^2^ g^−1^ based on CO_2_ adsorption isotherm at 195 K (fig. S7).

The vapor adsorption isotherms of PX, MX, OX, and EB on Ni(BIC)_2_ were measured at 303 to 393 K (Fig. 2, B and C, and fig. S8). Ni(BIC)_2_ shows strong preferential affinity for PX at all temperatures, with uptake reaching 1.8 mmol g^−1^ (∼191 mg g^−1^) at 1.2 kPa. Even at 393 K, PX uptake remains high (1.86 mmol g^−1^), surpassing commercial zeolites such as BaX (0.98 mmol g^−1^) (*12*) and ZSM-5 (1.34 mmol g^−1^) (*13*), as well as most MOFs (table S3). In contrast, MX, OX, and EB display temperature-dependent, stepwise adsorption, with saturated uptakes decreasing markedly with temperature, MX (95.1%), OX (92.2%), and EB (33.2%), whereas PX uptake remains nearly constant.

The isotherm shapes also vary with temperature, from steep rises at low temperature to S-shaped profiles at higher temperature, indicating guest-specific structural transitions in Ni(BIC)_2_. A notable “pressure-reducing” phenomenon appears in MX isotherms at 303 and 333 K, suggesting a temporary deviation from the equilibrium pressure due to crystal cell expansion. Ni(BIC)_2_ adsorbs negligible OX except at 303 K, implying OX exclusion across 303 to 393 K and simultaneous MX and OX exclusion at ≥363 K (Fig. 2C and fig. S9). This temperature-dependent thermodynamic sieving, together with a PX shape-matching channel, underpins the framework’s unique selectivity. To preliminarily assess the separation performance, the separation potential (Δ*q*) (*40*, *41*) of Ni(BIC)_2_ was calculated to be 9.36 at 393 K (table S4). Differential scanning calorimetry (DSC) confirms these trends, with heats of adsorption of PX (−74.5 kJ mol^−1^), EB (−55.1 kJ mol^−1^), MX (−44.5 kJ mol^−1^), and negligible for OX (fig. S10).

To understand the diffusion behavior and kinetics of C8 aromatics adsorption in Ni(BIC)_2_, vapor-phase adsorption kinetics experiments were conducted simultaneously with the isotherm measurements. The adsorption uptake curves as a function of time (fig. S11) were fitted with the linear driving force model (*42*) to extract the mass-transfer coefficient (*k*_s_*a*_p_, s^−1^) (table S5). At 303 K, the *k*_s_*a*_p_ value for PX was estimated to be 6.49 × 10^−5^ s^−1^, which is approximately an order of magnitude higher than that of MX (4.14 × 10^−6^ s^−1^). This substantial difference in diffusion rates results in a high PX/MX kinetic selectivity of 15.7 at 303 K, consistent with the comparison of PXRD patterns of Ni(BIC)_2_ samples loaded with different isomers for soaking time of 15 min and 12 hours (fig. S12). These results indicate that, in addition to thermodynamic molecular sieving effects, faster diffusion kinetics also facilitate effective separation of PX from MX at lower temperatures in Ni(BIC)_2_. The rapid PX diffusion kinetics were maintained at higher temperatures as well, with the *k*_s_*a*_p_ reaching 5.83 × 10^−4^ s^−1^ at 393 K, outperforming the value of 2.09 × 10^−5^ s^−1^ at the same temperature for zeolite ADS-37 used in the UOP Parex process (fig. S11). This represents nearly an order of magnitude increase compared to 303 K, highlighting the advantage of high-temperature operation.

To assess the liquid-phase separation performance of Ni(BIC)_2_ for xylene isomers, adsorption experiments were conducted on binary, ternary, and quaternary mixtures (Fig. 2, E and F; figs. S13 to S16; and table S6). In binary systems (PX/OX, PX/MX, and PX/EB) at 333 and 393 K, equilibrium data fitted well with the Stuart model (*43*), with mean deviations below 3.3% (Fig. 2E, fig. S17, and table S7). The average separation factors (α_avg_) reached 176.8 (PX/OX), 32.8 (PX/MX), and 3.7 (PX/EB), consistent with batch adsorption experiments (*S*_PX/OX_ = 56.1, *S*_PX/MX_ = 21.8, and *S*_PX/EB_ = 3.1). For equimolar ternary PX/MX/OX mixtures, PX selectivity increased with temperature, from 32.8/3.4/1 at 303 K to 77.1/4.4/1 at 393 K. Figure 2G compares the *S*_PX/(MX+OX)_-PX capacity performance of Ni(BIC)_2_ with reported zeolites, experimental MOFs, and HTCS (High-Throughput Computational Screening) MOFs. In quaternary mixtures mimicking industrial feeds (PX/MX/OX/EB = 22/50/22/6), Ni(BIC)_2_ preferentially adsorbed PX with selectivities of 57.8/3.8/1/23.3 at 303 K and 155.5/7.1/1/49.0 at 393 K (table S6), comparing favorably with benchmark adsorbents such as AgLClO_4_ (24/3.9/1/2.4) and Zn-MOF (50/4.2/1/7.9) (table S3).

To elucidate the C8 separation mechanism on Ni(BIC)_2_, we used a combination of SCXRD, microcrystal electron diffraction (MicroED), Rietveld refinement, high-resolution transmission electron microscopy (HRTEM), and in situ PXRD to investigate the structural transformation of Ni(BIC)_2_ upon exposure to C8 isomers. Initially, SCXRD revealed that the as-synthesized Ni(BIC)_2_ crystallizes in the P4_3_2_1_2 space group with cell parameters of *a* = *b* = 14.9659(7) Å, *c* = 8.3925(4) Å, and α = β = γ = 90° [Ni(BIC)_2_ (**1**)] (table S2). To verify the structural changes, high-resolution electron diffraction (ED) data were collected from guest-adsorbed Ni(BIC)_2_ crystals using MicroED. The results showed that Ni(BIC)_2_ underwent a SCSC transformation from a tetragonal lattice in space group P4_3_2_1_2 (**1**) to an orthorhombic lattice in space group C222_1_ with *a* = 20.69 Å, *b* = 23.88 Å, and *c* = 7.84 Å [Ni(BIC)_2_ (**2**)] (Fig. 2A and table S2), indicating that the channel shape adapts to the guest molecules and the primitive cell expands by 1.3%. Notably, across all C8 isomers investigated, only these two framework phases (**1**) and (**2**) were observed. If we draw an analogy between the adsorption process and a reversible chemical reaction, then the ratio of deformed to nondeformed crystal can be analogous to the chemical equilibrium constant *K* of the reactants. To confirm the transformation ratios, Rietveld refinement was conducted with the PXRD pattern of Ni(BIC)_2_ after exposure to PX and EB (figs. S18 and S19). The ratio of **2**/**1** after PX adsorption was 7.9, whereas that after EB adsorption was 2.4, which well explains the intriguing guest-dependent adsorption behavior of Ni(BIC)_2_.

**Fig. 3.**
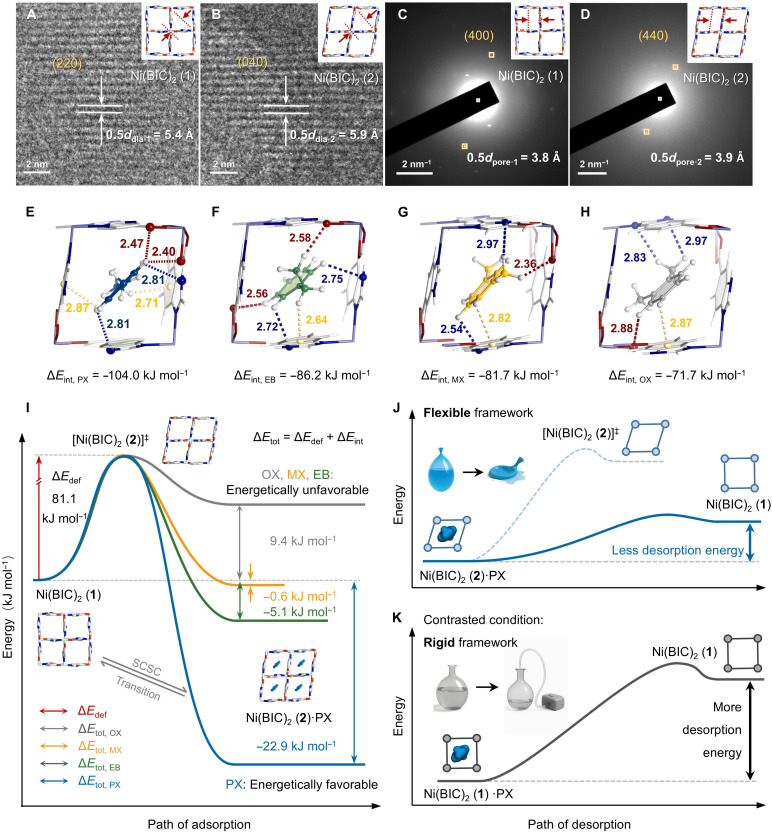
Guest-specific structural transformation of Ni(BIC)_2_. (**A** to **D**) HRTEM images along the direction of pore-diagonal (A) before and (B) after PX adsorption, and the fast Fourier transform images along the direction of pore diameter (C) before and (D) after PX adsorption. (**E** to **H**) DFT calculated adsorption binding sites and interaction energy (Δ*E*_int_) between xylene isomer molecules and frameworks for (E) PX, (F) EB, (G) MX, and (H) OX, respectively (color code: Ni, purple; C, gray; O, red; N, blue; H, white. The unit of distance is angstrom). (**I**) Total adsorption energy (Δ*E*_tot_) and framework deformation energy (Δ*E*_def_) diagram of xylene isomers on Ni(BIC)_2_ (color code: PX, blue; EB, green; MX, yellow; OX, gray). (**J** and **K**) Illustration of desorption mechanisms in (J) flexible and (K) rigid frameworks. Noted that the adsorption/desorption process is illustrated in two steps, with [Ni(BIC)_2_(**x**)] denoting different framework states: [Ni(BIC)_2_(**1**)] for the undeformed, guest-free framework; [Ni(BIC)_2_(**2**)]^‡^ for the deformed, guest-free framework; and [Ni(BIC)_2_(**2**)] for the deformed, guest-loaded framework. In the real adsorption/desorption process, these two steps are supposed to occur simultaneously, and [Ni(BIC)_2_(**2**)]^‡^ is a hypothetical state.

Furthermore, HRTEM images of **1** and **2** (before and after PX adsorption) were compared to verify the crystal transformation. HRTEM images and fast Fourier transform images (Fig. 3, A to D) show the lattice fringes of ·**1** and·**2** with 0.5*d*_dia_ spacing distributions of ∼5.4 (0.5*d*_dia·**1**_) and 5.9 Å (0.5*d*_dia·**2**_) and 0.5*d*_pore_ of 3.8 (0.5*d*_pore·**1**_) and 3.9 Å (0.5*d*_pore·**2**_), respectively (where *d*_dia_ and *d*_pore_ represent length of the pore-diagonal and the pore size, respectively). These values are in good agreement with the changes in 2θ [2θ_dia_, 16.42° (**1**) to 14.82° (**2**); 2θ_pore_, 23.30° (**1**) to 22.72° (**2**)] and *d* spacing [0.5*d*_dia_, 5.395 (**1**) to 5.970 Å (**2**); 0.5*d*_pore_, 3.815 (**1**) to 3.909 Å (**2**)] obtained from the MicroED-obtained structure models of **1** and **2**. The alterations in lattice spacing observed in *d*_dia_ and *d*_pore_ represent the elongation of the pore-diagonal and the pore size of Ni(BIC)_2_ after PX adsorption. To complement these findings, in situ PXRD measurements were conducted under an atmosphere switched from nitrogen to PX vapor (fig. S20). Notably, the predominant (200) diffraction peak of Ni(BIC)_2_ (**1**) vanished markedly upon PX introduction, while a new peak emerged at 2θ = 11.3°, corresponding to a *d*-spacing of 7.8 Å [attributed to the (220) plane of Ni(BIC)_2_ (**2**)]. These changes signify the transformation of Ni(BIC)_2_ from a tetragonal to an orthorhombic lattice, indicating adaptation into the molecular projection shape channel via a solid-state guest-induced crystal transformation.

To unravel the mechanism behind the PX preference in Ni(BIC)_2_ and illustrate our contrast enhancement strategy, we established the adsorption state of C8 aromatics in Ni(BIC)_2_ using first principles dispersion-corrected density functional theory (DFT-D) calculations (Fig. 3, E to I). The optimized C8 molecules were placed in the channel along the longer diagonal direction, which was consistent with the electronic density distribution in the crystal structure obtained by MicroED. The structure [Ni(BIC)_2_@guest] was then optimized by relaxing the guest positions while holding the host framework fixed. The DFT-D calculations confirmed the presence of multiple weak interactions between the guest molecules and the framework, including C─H⋯O, C─H⋯N, and C─H⋯π interactions. To quantify the guest-specific response, we analyzed the guest interaction energy (Δ*E*_int_), framework deformation energy (Δ*E*_def_), and total adsorption energy (Δ*E*_tot_), where Δ*E*_tot_ is calculated as the sum of Δ*E*_int_ and Δ*E*_def_. The calculated Δ*E*_int_ values for PX, EB, MX, and OX were calculated to be −104.0, −86.2, −81.7, and −71.7 kJ mol^−1^, respectively (Fig. 3, E to H, and table S8). The higher negative value of Δ*E*_int_ for PX compared to EB, MX, and OX indicates much stronger host-guest interactions. However, the differences in Δ*E*_int_ alone were insufficient to explain the selective PX adsorption.

Crucially, the flexibility of Ni(BIC)_2_ imposed a Δ*E*_def_ of 81.1 kJ mol^−1^ required for framework deformation. The occurrence of a SCSC transition is indicated when Δ*E*_int_ dominates over Δ*E*_def_, resulting in a negative Δ*E*_tot_. Consequently, Δ*E*_tot_ values for PX, EB, MX, and OX were calculated to be −22.9, −5.1, −0.6, and 9.4 kJ mol^−1^, respectively, thereby amplifying the differences in Δ*E*_tot_ between PX and other isomers, making PX the energetically favorable guest for inducing the dynamic structural transformation from the tetragonal (**1**) to orthorhombic phase (**2**) (Fig. 3I, table S8, and fig. S21). For EB, MX, and OX, the lower magnitude of Δ*E*_tot_ meant that they could not overcome the energy barrier of structural change, resulting in their exclusion from PX. These density functional theory (DFT) results provide insights into how the integration of binding interactions and framework flexibility enables targeted molecular recognition in Ni(BIC)_2_.

The guest-specific structural transformation reveals the mechanism of molecular projection shape discrimination. The projection shape can be quantified by the minimum and second-minimum dimensions (“MIN-1” and “MIN-2”) (*44*). While PX, MX, and OX have similar MIN-1 values (3.810, 3.949, and 3.834 Å, respectively), their MIN-2 differs, making it the decisive factor (PX of 6.618 Å versus MX of 7.258 Å and OX of 7.269 Å). The channel diagonal (7 Å) lies between these values, allowing PX with the smaller MIN-2 to be preferentially trapped. In contrast, PX and EB share similar MIN-2 (6.618 versus 6.625 Å), but EB’s larger MIN-1 (5.285 versus 3.810 Å) renders it unfavorable for channel transformation (fig. S1 and table S9). This enables complete PX/EB separation by tuning temperature. Thus, the PX preference of Ni(BIC)_2_ arises from the synergy of projection shape matching and framework flexibility.

We further elucidated how structural flexibility controls the desorption step. The contrast between flexible and rigid frameworks is akin to a balloon and a glass vessel. In Ni(BIC)_2_, elastic recovery drives xylene release, while rigid frameworks desorb only via displacement (Fig. 3J). Under identical conditions, thermogravimetric measurements show faster desorption from Ni(BIC)_2_ than from zeolite (fig. S22), demonstrating the intrinsic productivity advantage of flexibility (*45*).

**Fig. 4.**
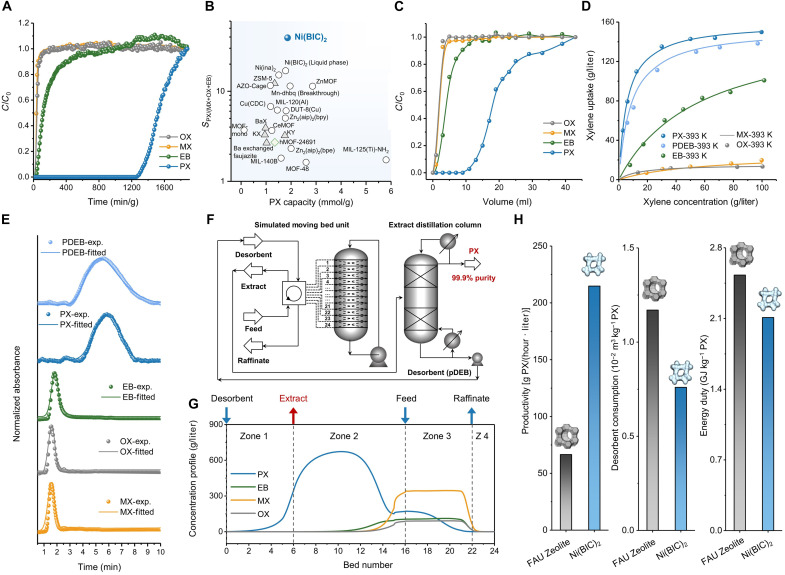
Separation performance and SMB process simulation results of Ni(BIC)_2_ for xylene isomer mixtures. (**A**) Vapor breakthrough curves of PX/MX/OX/EB mixtures with a molar ratio of 22:50:22:6 on Ni(BIC)_2_ at 393 K. (**B**) Comparison of PX separation performance using an *S*_PX/(MX+OX+EB)_-PX capacity plot. Data are collected from reported adsorbents (table S3), with the highest reported selectivity for each material adopted for comparison. (**C**) Liquid phase breakthrough curve on Ni(BIC)_2_ column under 393 K. (**D**) The liquid adsorption isotherms of PX, MX, OX, EB, and PDEB on Ni(BIC)_2_ column at 393 K. (**E**) Pulse curves of PX, MX, OX, and EB on Ni(BIC)_2_ column at 393 K. (**F**) Schematic illustration of the four-zone SMB process and downstream PX/pDEB distillation. (**G**) Internal concentration profiles across the 24 beds at cyclic steady state of the SMB process on Ni(BIC)_2_. (**H**) Comparison of productivities, desorbent consumptions, and energy duties in the SMB process on Ni(BIC)_2_ and FAU zeolite.

To evaluate the separation capacity of Ni(BIC)_2_ for mixed C_8_ aromatics, fixed-bed dynamic breakthrough and desorption experiments were conducted with binary, ternary, and quaternary feeds (Fig. 4, A to C, and figs. S23 to S27). A Ni(BIC)_2_-packed column [4.6 mm inside diameter (ID) by 30 mm] was tested at 15 ml min^−1^. The breakthrough curves confirmed superior PX purification consistent with single-component adsorption isotherms and kinetics. Ni(BIC)_2_ achieved molecular sieving of PX from PX/MX/OX at 303 K and, notably, extracted PX from PX/MX/OX/EB mixtures at 393 K (22/50/22/6, identical to the UOP Parex feed), demonstrating the feasibility of efficient PX isolation (Fig. 4A). The dynamic selectivities at 393 K (*S*_PX/MX_ = 38.8, *S*_PX/OX_ = 116.9, and *S*_PX/EB_ = 11.7) compare favorably with Mn-dhbq (24.0, 47.8, and 1.4) and ADS-37 (2.3, 2.3, and 1.8) (Fig. 4C and fig. S28) and demonstrate competitive performance for MOF-based quaternary separation (figs. S29 and S30). These dynamic selectivities are much higher than the static ones (21.8, 56.1, and 3.1), reflecting the synergy of thermodynamics and kinetics: PX combines higher capacity with faster diffusion. To provide a more comprehensive comparison, the *S*_PX/(MX+OX+EB)_-PX capacity plot is shown in Fig. 4B. It should be noted that the PX uptake of Ni(BIC)_2_ is moderate compared to some reported materials but remains competitive as many high-capacity materials are evaluated at near-ambient temperatures, whereas Ni(BIC)_2_ is measured under elevated temperatures. Desorption under N_2_ at 423 K yielded PX with 95% purity and 69% recovery from the quaternary mixture in a single cycle (fig. S31). Moreover, liquid-phase breakthrough experiments at 393 K revealed excellent separation of PX from a quaternary C8 aromatic mixture, with PX exhibiting substantially longer retention than MX, OX, and EB (Fig. 4C), representing the first reported MOF-based quaternary xylene separation in the liquid phase.

To benchmark Ni(BIC)_2_ against FAU-type zeolite, we conducted comprehensive SMB process simulations using experimentally determined parameters (Fig. 4, D to G). Single-batch experiments confirmed retention of high PX selectivity (PX/MX/OX/EB = 76.9/3.3/1/19.0) in 50% *p*-diethylbenzene (pDEB) solution (figs. S32 and S33). Liquid-phase isotherms and mass-transfer coefficients extracted from chromatographic analysis (Fig. 4, D and E, and tables S10 and S11) enabled rigorous SMB modeling via Aspen Chromatography (Fig. 4F and figs. S34 and S35). Parallel simulations of 24-bed FAU zeolite systems used established parameters from the literature (table S12) (*46*, *47*). Under substantially milder conditions (393 K versus 453 K), Ni(BIC)_2_ achieved productivity of 214.78 kg of PX hour^−1^ m^−3^ and processing capacity of 1.1652 m^3^ of xylene hour^−1^ m^−3^, representing 3.2-fold and 3.0-fold improvements over FAU zeolites, respectively (Fig. 4H and table S12). Critically, these performance gains were achieved while maintaining industrial specifications: 99.98% purity, 99.95% recovery, and competitive solvent consumption (7.6 liters kg^−1^ PX versus 11.7 liter kg^−1^ PX for FAU) (Fig. 4F). Upon the SMB results, distillation simulations were further conducted to quantify the energy required for PX/pDEB separation in the downstream step. The Ni(BIC)_2_-based process exhibited significantly lower energy consumption (2.11 MJ kg^−1^ PX) than the conventional zeolite-based process (2.52 MJ kg^−1^ PX) (Fig. 4H and table S13).

Ni(BIC)_2_ showed high stability in the presence of PX and air as the crystallinity of Ni(BIC)_2_ samples remained intact after immersion in pure PX and a quaternary xylenes mixture at 393 K for 1 week, after 30 cycles, and even exposure to air for 3 months (figs. S36 and S37). Recyclability tests for PX adsorption-desorption were conducted on Ni(BIC)_2_ samples in both liquid and vapor phases at 393 K. These tests confirmed that Ni(BIC)_2_ could be regenerated at 453 K without PX uptake loss in either phase. Additionally, the long-term liquid-phase adsorption cycling experiments with a hexanary mixture of PX/MX/OX/EB/pDEB/toluene (mass ratio, 11/25/11/3/49.25/0.75) demonstrated that Ni(BIC)_2_ maintained its PX selectivity and capacity over 30 cycles (fig. S38).

In summary, this work presents Ni(BIC)_2_, an adaptable MOF that exhibits high PX selectivity for C8 aromatic isomers at elevated temperatures. The framework has a shape-matching channel that enables preferential binding of PX. The framework’s flexibility significantly amplifies total adsorption energy differences between PX and other isomers through a contrast enhancement mechanism. This synergy between shape matching and dynamic structural transformations allows Ni(BIC)_2_ to achieve high PX separation performance. Temperature-independent PX uptake, complete OX exclusion up to 393 K, and competitive selectivities for multicomponent mixtures demonstrate promising performance relative to traditional adsorbents. This rational design approach helps address the selectivity-temperature trade-off commonly encountered in conventional adsorbents and provides a promising platform for developing next-generation adsorbents for efficient xylene separation.

## MATERIALS AND METHODS

### Materials

All reagents and solvents were obtained from commercial sources and used as received without further purification. HBIC (99%) was purchased from TCI (Japan). Nickel(II) nitrate hexahydrate [Ni(NO_3_)_2_·6H_2_O, 99%], acetonitrile (CH_3_CN; 99%), formic acid (HCOOH; 99%), methanol (CH_3_OH; 99%), hydrochloric acid (HCl; 12 M), and dichloromethane (CH_2_Cl_2_; 99%) were purchased from Sinopharm (China). The C8 isomers, including PX (99%), OX (99%), MX (99%), EB (99%), and pDEB (98%), as well as toluene (Tol; 99.9%), were provided by Sigma-Aldrich (USA). Ultrahigh-purity gases, including carbon dioxide (99.999%), helium (99.999%), and nitrogen (99.999%), were supplied by Jingong Industiral Co. (China).

### Methods

#### *Synthesis of Ni*(*BIC*)*_2_·2.5H_2_O*

The Ni(BIC)_2_·2.5H_2_O material was synthesized using a modified version of a method previously reported (*39*). Specifically, Ni(NO_3_)_2_·6H_2_O (0.1 mmol, 29.1 mg) and HBIC (0.1 mmol, 16.3 mg) were dissolved in a mixture of H_2_O (3 ml) and CH_3_CN (3 ml). To this solution, a small volume of HCOOH (6 μl) was added to improve the phase purity of the resulting material. The mixture underwent ultrasonic treatment for 20 min, after which it was sealed in a 20-ml Teflon-lined reactor. The reactor was then placed in a preheated oven at 433 K for 48 hours. Following this incubation, the autoclave was cooled down to room temperature. The resulting green, rod-shaped crystals were thoroughly washed with water and methanol, and then dried in air. The product yield was 49.4%.

#### Characterization

PXRD patterns were collected on a SmartLab SE diffractometer (Rigaku, Japan) using Cu Kα radiation. Rietveld refinements were performed using the TOPAS package (*48*). Variable-temperature PXRD (VT-PXRD) and in situ PXRD measurements were conducted on a Rigaku SmartLab diffractometer equipped with a rotating-anode Cu Kα source and a ReactorX sample chamber. VT-PXRD data were collected from 303 to 773 K under flowing N_2_, while in situ PXRD measurements were performed during exposure to PX vapor at 303 K after activation of the samples at 423 K under vacuum. Thermogravimetric analysis was carried out on an SDT 650 simultaneous thermal analyzer (TA Instruments, USA) under N_2_. CO_2_ adsorption-desorption isotherms at 195 K were measured on an ASAP 2020 analyzer (Micromeritics, USA) for surface area and pore analysis. SCXRD data were collected on a Bruker D8 Venture diffractometer at 170 K, and structures were solved and refined using Olex2, SHELXT, and SHELXL (*49*–*51*). Scanning electron microscopy and HRTEM images were acquired using Hitachi S-3400 and JEOL JEM-2100F microscopes, respectively. Additional instrumental parameters are provided in the Supplementary Text.

#### Vapor adsorption experiments

Approximately 40 mg of the as-synthesized sample was transferred into an empty, preweighted sample tube and degassed at 423 K under high vacuum for 24 hours to ensure complete activation. The tube containing the activated sample was reweighed to determine the precise amount of the activated adsorbent. The activated sample was then used to acquire single-component xylene adsorption isotherms at 303, 333, 363, and 393 K on a BELSORP MAX II vapor adsorption analyzer (MicrotracBEL, Japan). Vapor adsorption kinetics were measured concurrently during the single-component adsorption experiments using the built-in kinetic measurement function of the instrument. Throughout the adsorption experiments, the vapor source and manifold system were maintained at 323 K. Temperature regulation during adsorption was achieved via a thermostatically controlled heating mantle encasing the sample tube.

#### *Adsorption heats of xylenes on Ni*(*BIC*)*_2_*

The adsorption heats of xylene isomers on Ni(BIC)_2_ were determined using DSC on a SDT 650 simultaneous thermal analyzer (TA Instruments, USA). Before the adsorption measurements, the thermal analyzer was calibrated to establish a stable heat flow baseline, using an inert atmosphere of high-purity nitrogen at 303 K. Following this, the activated adsorbent, weighing ∼5 mg, was exposed to a diluted mixture of xylene isomers in nitrogen. This mixture was prepared by sparging the nitrogen carrier gas through a liquid xylene isomer-filled bubbler, ensuring a consistent vapor-phase introduction to the sample chamber, also maintained at 303 K. The real-time data acquisition of both DSC and thermogravimetric signals facilitated the simultaneous determination of the adsorption amount and the corresponding heats of adsorption for the xylene isomers.

#### Vapor-phase multicomponent breakthrough measurements

Vapor-phase breakthrough experiments were conducted using binary, ternary, and quaternary mixtures of xylene isomers as the feed. Before each measurement, Ni(BIC)_2_ was activated at 423 K under ultrahigh vacuum for 24 hours and packed into a stainless-steel column (4.6 mm ID by 30 mm, 0.4 g of adsorbent) in an N_2_-filled glovebox. A nitrogen carrier gas (99.999%) was introduced at 15 ml min^−1^ to deliver xylene vapors generated from bubblers maintained at 298 K. For the quaternary mixture, the partial pressure ratio of PX/MX/OX/EB was set at 22/50/22/6, while equimolar compositions were used for ternary and binary mixtures. The outlet stream was analyzed using a GC-2010 Pro gas chromatograph (Shimadzu, Japan) equipped with an FID detector and a PEG-20 M capillary column. After each run, the sample was regenerated under flowing N_2_ at 423 K for 48 hours. Additional details are provided in the Supplementary Text.

#### Multicomponent liquid-phase breakthrough experiments

Liquid-phase breakthrough experiments were conducted using mixtures of xylene isomers dissolved in 1,3,5-trimethylbenzene (TMB) as the feed. Before measurement, the Ni(BIC)_2_-packed column (4.6 mm ID by 25 mm) was flushed with pure TMB at 393 K to establish equilibrium. Following equilibration, a feed solution containing PX, MX, OX, and EB with a molar ratio of 22/50/22/6 and a total concentration of 0.19 M in TMB was continuously introduced using a high-performance liquid chromatography pump at a flow rate of 0.1 ml min^−1^ at 393 K. Effluent samples were collected from the column outlet at fixed time intervals and analyzed by gas chromatography (GC) under conditions identical to those used for vapor-phase breakthrough measurements. Breakthrough curves were recorded until the outlet concentration of each xylene isomer approached that of the inlet feed.

#### *Calculation of ternary selectivity S*_*PX/*(*MX+OX*)_ *and quaternary selectivity S*_*PX/*(*MX+OX+EB*)_

To provide a more consistent comparison of PX-selective separation performance across different reported materials, combined ternary and quaternary selectivities were calculated from the reported pairwise selectivities obtained under the same multicomponent conditions. The ternary selectivity *S*_PX/(MX+OX)_ was calculated according to

$$S_{\text{PX}/\left(\text{MX}+\text{OX}\right)}=\frac{x_{\text{MX}}+x_{\text{OX}}}{\frac{x_{\text{MX}}}{S_{\text{PX}/\text{MX}}}+\frac{x_{\text{OX}}}{S_{\text{PX}/\text{OX}}}}$$

where *x_i_* represents the mole fraction of component *i* in the feed mixture and *S*_PX/*i*_ is the reported pairwise selectivity between PX and component *i*.

Similarly, the quaternary selectivity *S*_PX/(MX+OX+EB)_ was calculated as

$$S_{\text{PX}/\left(\text{MX}+\text{OX}+\text{EB}\right)}=\frac{x_{\text{MX}}+x_{\text{OX}}+x_{\text{EB}}}{\frac{x_{\text{MX}}}{S_{\text{PX}/\text{MX}}}+\frac{x_{\text{OX}}}{S_{\text{PX}/\text{OX}}}+\frac{x_{\text{EB}}}{S_{\text{PX}/\text{EB}}}}$$

For materials reported under equimolar feed conditions, the corresponding feed compositions were assumed to be equal unless otherwise specified in the original literature.

#### Structure determination by ED

For ED analysis, activated samples were contacted with PX, EB, MX, or OX and dispersed onto carbon-coated copper grids, followed by cryogenic preservation in liquid nitrogen. Data were collected at 77 K on a JEOL JEM-2100Plus transmission electron microscope operated at 200 kV and equipped with a direct electron detector. Diffraction datasets were processed using REDp (*52*), XDS (*53*), and Coeus software (ReadCrystal Tech, China), and structure models were solved using SHELXT (*50*). Additional experimental details are provided in the Supplementary Text.

#### DFT calculations

DFT calculations were carried out using the CASTEP code (*54*). The Perdew-Burke-Ernzerhof functional within the generalized gradient approximation was used to describe the exchange-correlation interactions (*55*). Dispersion interactions were described using the semi-empirical Grimme G06 correction scheme as implemented in CASTEP, with the default short-range damping function applied (*56*). A 1 × 1 × 2 supercell was constructed to accommodate the xylene molecules within the pore channels as the molecular length exceeds the dimensions of the primitive unit cell. A plane-wave cutoff energy of 544 eV and a 2 × 2 × 2 *k*-point mesh were adopted. Detailed convergence tests are provided in the Supplementary Materials.

For the evaluation of Δ*E*_int_, the framework structures were taken from experimentally determined MicroED models. In particular, the deformed Ni(BIC)_2_ (2) structure obtained from ED measurements was used after removal of the guest molecules. Then, xylene molecules were introduced into the channels to construct initial adsorption configurations. Last, constrained geometry optimizations were subsequently performed, in which the framework atoms were kept fixed and only the guest molecules were allowed to relax, yielding the host-guest interaction energies. Δ*E*_int_ was calculated using the equation Δ*E*_int_ = *E*_(absorbed adsorbent+xylene)_ − *E*_(xylene)_ − *E*_(absorbed adsorbent)_. The structural deformation energy (Δ*E*_def_) was calculated as the energy difference between the experimentally determined Ni(BIC)_2_ (1) and Ni(BIC)_2_ (2) framework structures, with equation Δ*E*_def_ = *E*_(adsorbed adsorbent)_ − *E*_(activated adsorbent)_. The total adsorption energy (Δ*E*_tot_) was then obtained as the sum of Δ*E*_int_ and Δ*E*_def_, with equation Δ*E*_tot_ = Δ*E*_int_ + Δ*E*_def_.

Here, Δ*E*_def_ represents the energetic cost associated with framework deformation during the structural transformation process. Experimental structural characterization indicates that different xylene isomers induce the same framework transformation from Ni(BIC)_2_ (1) to Ni(BIC)_2_ (2) and, therefore, are expected to experience similar deformation barriers associated with this common structural transition. In this case, the selective adsorption behavior is associated with whether the host-guest interaction energy is sufficient to overcome this common energetic threshold. The DFT calculations at 0 K are used to capture relative energetic trends underlying the selective transformation behavior, rather than to provide exact quantitative predictions of finite-temperature adsorption uptake.

#### Batch adsorption-desorption experiments in eluent

The potential application of Ni(BIC)_2_ within SMB processes was assessed through liquid-phase adsorption and desorption experiments using pDEB as an eluent. An activated sample of Ni(BIC)_2_, weighing ∼200 mg, underwent activation under vacuum conditions at 423 K for 24 hours. This activated sample was then immersed in a 10-ml mixture of PX/MX/OX/EB/pDEB with varying mass ratios: 11/25/11/3/50, 4.4/10/4.4/1.2/80, 2.2/5/2.2/0.6/90, and 1.1/2.5/1.1/0.3/95 at 393 K for 3 hours. Following immersion, the samples from the mixture with a mass composition of 11/25/11/3/50 were filtered and then air-dried at room temperature for 2 hours to conduct the desorption experiment. Subsequently, 10 mg of the resultant sample was digested with 2 ml of 12 M hydrochloric acid overnight. Aromatics were extracted from the digestion solution using 2 ml of dichloromethane, and the isolated organic phase was analyzed by GC to determine the mass ratios of the aromatics. The overall uptake amount was quantified by TG analysis, and the adsorption selectivity in the presence of the eluent was calculated using eq. S15. Following adsorption, elution experiments were conducted by agitating 100 mg of the adsorption-saturated adsorbents in 5 ml of pure pDEB at 393 K for 2 hours. The mixture was then filtered using a 0.22-μm organic membrane to separate the supernatants from the adsorbent. The solid residue, after air-drying for 2 hours, was subjected to the same digestion and extraction protocol as in the adsorption phase. Both the organic extract and the supernatants, before and after elution, were analyzed under identical GC conditions to determine the purity and recovery rate of PX during a single elution cycle.

#### Liquid-phase adsorption and mass-transfer measurements

Liquid-phase adsorption isotherms were measured using a fixed-bed chromatographic method following reported procedures (*57*, *58*). A stainless-steel column (15 cm by 0.46 cm ID) packed with Ni(BIC)_2_ (2.1 g, 300 mesh) was operated at 393 K using TMB as the mobile phase. Feed solutions containing xylene isomers were continuously introduced until equilibrium was reached, and adsorbed amounts were determined from desorption mass balances. Equilibrium data were fitted using Langmuir and extended Langmuir models (*59*). Mass-transfer kinetics were evaluated by pulse chromatography on a Ni(BIC)_2_-packed column using TMB as the carrier fluid at 393 K. Kinetic parameters were obtained by fitting pulse-response curves with an axial-dispersion model coupled with linear driving-force mass transfer (*60*, *61*). Additional experimental details and equations are provided in the Supplementary Text.

#### SMB process simulation

A four-zone SMB process with 24 columns was simulated in Aspen Chromatography to evaluate PX separation performance using Ni(BIC)_2_ and FAU zeolite adsorbents (*62*–*66*). The model considered adsorption equilibrium, axial dispersion, and mass-transfer resistance using experimentally determined thermodynamic and kinetic parameters. Operating windows were first estimated using triangle theory and then optimized by dynamic simulations. Product purity, recovery, productivity, solvent consumption, and processing capacity were used as performance metrics. Full model equations and simulation details are provided in the Supplementary Text.

#### PX/PDEB distillation simulation

Steady-state distillation simulations for PX/PDEB separation were performed in Aspen Plus V11 using the RadFrac model with the NRTL thermodynamic method (*67*). Feed conditions were based on the SMB extract stream, and operating parameters were optimized to achieve target PX purity while minimizing energy consumption. Additional simulation details are provided in the Supplementary Text.

#### Stability and recyclability tests

The stability of Ni(BIC)_2_ was evaluated after exposure to pure PX, quaternary PX/MX/OX/EB mixtures, and ambient air under representative conditions, followed by PXRD analysis to examine structural integrity. Recyclability was assessed through repeated adsorption-desorption cycles in both vapor and liquid phases at 393 K. In vapor-phase cycling, PX adsorption and thermal regeneration were conducted under flowing N_2_ using a thermogravimetric analyzer. In liquid-phase cycling, PX-loaded samples were regenerated thermally after adsorption. Long-term cycling performance was further examined using a multicomponent aromatic mixture containing xylene isomers, toluene, and pDEB. Adsorption capacities and selectivities during cycling were monitored by TG and GC analyses. Additional experimental details are provided in the Supplementary Text.

## Supplementary Materials

### The PDF file includes:

Supplementary Text Figs. S1 to S40 Tables S1 to S16 Legends for data S1 and S2 References
