# Supplementary material for: Separation of xylene isomers in an adaptable metal-organic framework
Source: Sci Adv. 2026 Aug 14;12(33):eaeg1691. doi: 10.1126/sciadv.aeg1691 (PMC13475607; doi:10.1126/sciadv.aeg1691)
Supplement: Supplementary file 1 [file sciadv.aeg1691_sm.pdf]

Science Advances
AAAS

Supplementary Materials for

Separation of xylene isomers in an adaptable metal-organic framework

Jiaqi Li *et al.*

Corresponding author: Qiwei Yang, yangqw@zju.edu.cn; Dan Zhao, chezhao@nus.edu.sg;
Zongbi Bao, baozb@zju.edu.cn

Sci. Adv. **12**, eaeg1691 (2026)
DOI: 10.1126/sciadv.aeg1691

The PDF file includes:

Supplementary Text
Figs. S1 to S40
Tables S1 to S16
Legends for data S1 and S2
References

Other Supplementary Material for this manuscript includes the following:

Data S1 and S2

Supplementary Text

Characterization. Powder X-ray diffraction (PXRD) patterns of the samples were obtained using a SmartLab SE powder X-ray diffractometer (Rigaku, Japan) with a Cu Kα radiation at 40 kV, 50 mA, in the 2θ range of 5-80° (0.02° step^{-1}) with a 1D scanning rate of 10° min^{-1}. Rietveld refinement of the samples was performed with the TOPAS (*48*) package in the 2θ range of 5-80° with Pseudo-Voigt profile function. For variable-temperature PXRD (VT-PXRD) and in-situ PXRD analyses, measurements were conducted on a SmartLab diffractometer (Rigaku, Japan) equipped with a high-intensity rotating anode Cu Kα source at 45 kV and 200 mA, and a ReactorX in-situ sample chamber. The VT-PXRD studies were carried out from 303 K to 773 K under a nitrogen atmosphere, with a controlled heating rate of 10 K min^{-1}. Data were collected within a 2θ range of 5 to 60°, using a step size of 0.02° and a scan rate of 20° min^{-1}. In-situ PXRD experiments were designed to capture structural transformations under a controlled atmosphere, transitioning from nitrogen to PX vapor, at 303 K. The data collection strategy was the same as for VT-PXRD, except the 2θ range was 10-30°. Prior to these measurements, powder samples were activated at 423 K under vacuum for 24 h to eliminate water and solvent molecules. Sample preparation was meticulously performed within a glovebox environment to prevent contamination from air and moisture. Thermal gravimetric (TG) analysis was carried out on an SDT 650 simultaneous thermal analyzer (TA Instruments, USA) with a heating rate of 10 K/min under a N_2 atmosphere, with the temperature ranging from 323 to 973 K. CO_2 adsorption-desorption isotherms at 195 K were obtained using an ASAP 2020 adsorption analyzer (Micromeritics, USA) to calculate the BET surface area and pore distribution of the sample. The system was allowed for an equilibration interval of 20 seconds to ensure accurate measurement of the gas uptake at the specified pressures.

Calculation of separation potential. The separation potential, denoted Δq (mol L^{-1}), was calculated using the combined selectivity/capacity metric proposed by Krishna et al. (*40*). The metric assesses the differential adsorption characteristics of an equimolar, industrial-relevant quaternary vapor mixture, composed of PX, OX, MX, and EB, with a preference for PX adsorption and exclusion of OX, MX, and EB:

$$\Delta q_{(\mathrm{OX+MX+EB})/\mathrm{PX}} = \left(q_{\mathrm{PX}}\right)\frac{y_{\mathrm{OX}}+y_{\mathrm{MX}}+y_{\mathrm{EB}}}{1-y_{\mathrm{OX}}-y_{\mathrm{MX}}-y_{\mathrm{EB}}}-\left(q_{\mathrm{OX}}+q_{\mathrm{MX}}+q_{\mathrm{EB}}\right) \qquad \text{(Eq. S1)}$$

Here, Δq represents the volumetric separation potential, q_i (mol L^{-1}) denotes the volumetric uptake capacities of each aromatic component on the adsorbent, and y_{i} is the mole fractions of component *i*. The composition of the quaternary xylene mixture was set as PX/OX/MX/EB = 1/1/1/1 for a fair comparison. To determine the uptake capacities, q_i in Eq. S1, pure component isotherms were integrated along with the Extended Langmuir model (*59*),

$$q_i = q_{sat,i}\frac{K_i P_i}{1+\sum_{j=1}^{n}\left(K_j P_j\right)} \qquad \text{(Eq. S2)}$$

where $q_{\mathrm{sat},i}$ (mol L^{-1}) is the saturated volumetric uptake capacity of each of the four isomers on the adsorbent, K_i represents Langmuir constants for each pure component i, and P_i (kPa) indicates the partial pressure of component i.

Vapor phase adsorption kinetic measurements. The vapor kinetic adsorption characteristics were measured concurrently with the single-component adsorption experiments on a BELSORP MAX II vapor adsorption analyzer (MicrotracBEL, Japan). Measuring vapor adsorption kinetics is a well-established function of the BEL vapor adsorption analyzer. During the measurement, the additional pressure drop caused by valves and sensors has been pre-calibrated, ensuring that the recorded pressure accurately reflects the adsorption behavior of the adsorbent. The kinetic profile corresponding to the point at ~ 0.1 kPa on the isotherm was used for mass-transfer coefficient fitting. With a dosing amount of 2 cm^3 g^{-1}, changes of pressures of the sample tube and manifold system were recorded over time (t, s) before and after vapor dosing until equilibrium was reached. These time-dependent changes were converted into transient adsorption amounts as functions of time. The mass-transfer coefficient ($k_{\mathrm{s}}a_{\mathrm{p}}$, s^{-1}) was fitted using the classical linear driving force (LDF) model (*42*), as expressed by the following equations,

$$\rho \frac{dq}{dt} = k_{\mathrm{s}} a_{\mathrm{p}} \left(C - C^{*} \right) \quad \text{(Eq. S3)}$$

$$k_{\mathrm{s}} = D_m / L \quad \text{(Eq. S4)}$$

where ρ is the density of adsorbent (kg m^{-3}), q is the average adsorption amount on adsorbent (mol kg^{-1}), k_{s} is the film mass-transfer coefficient (m s^{-1}), a_{p} is the specific surface area of adsorbent (m^2 m^{-3}), C is the fluid phase density (mol m^{-3}), C^* is the virtual adsorbate density on pore surface corresponding to an average adsorption amount (mol m^{-3}), D_{m} is the molecule diffusion coefficient (m^2 s^{-1}), L is the film thickness (m). Through the LDF Eq. S3, the adsorbate density (C) is divided by the adsorbent density (ρ), which is expressed by

$$\frac{dq}{dt} = k_{\mathrm{s}} a_{\mathrm{p}} \left(q - q^{*} \right) \quad \text{(Eq. S5)}$$

With Henry (linear) adsorption equilibrium equation in a narrow pressure zone, the relationship between amount of adsorption (q, mmol g^{-1}) and pressure (p, kPa) can be expressed by

$$q = Hp \quad \text{(Eq. S6)}$$

where H (mmol g^{-1} kPa^{-1}) is the Henry equilibrium constant. Considering the adsorption measurement on a single pressure point is a batch operation, Eq. S7 for batch adsorption operation is used,

$$W \left(q - q_0 \right) = \frac{V \left(p_0 - p \right)}{RT} \quad \text{(Eq. S7)}$$

where W is the mass of adsorbent (g), V is the total fluid volume (m^3), R is the gas constant, T is the temperature (K), and the subscript 0 stands for the initial state. Eq. S8 is then derived from Eqs. S5-S7,

$$q=\left(\frac{\alpha}{\alpha+1}\right)Hp_0-\left(\frac{\alpha}{\alpha+1}\right)Hp_0\exp\left(-\left(\frac{\alpha}{\alpha+1}\right)k_\mathrm{s}a_\mathrm{p}t\right) \quad \text{(Eq. S8)}$$

where $\alpha = V/(W\,H)$ is the separation factor. As separation factor α becomes smaller, it indicates that adsorbate shifts from fluid phase to solid phase. By solving Eq. S8 for p/p_0, the following equation is obtained:

$$\frac{p}{p_0}=1-\left(\frac{1}{\alpha+1}\right)\left[1-\exp\left(-\left(\frac{\alpha+1}{\alpha}\right)k_\mathrm{s}a_\mathrm{p}t\right)\right] \quad \text{(Eq. S9)}$$

Eq. S10 was obtained by solving the equation for the relationship between amount of adsorption and pressure before and after adsorption measurement at a single point of the isotherm,

$$\frac{p-p_\mathrm{e}}{p_0-p_\mathrm{e}}=1-\left(\frac{p_0-p_\mathrm{d}}{p_0-p_\mathrm{e}}\right)\left(\frac{1}{\alpha+1}\right)\left[1-\exp\left(-\left(\frac{\alpha+1}{\alpha}\right)k_\mathrm{s}a_\mathrm{p}t\right)\right] \quad \text{(Eq. S10)}$$

where p_d and p_0 are pressures of the initial state before and after vapor dosing, respectively, and p_e is the equilibrium pressure at the present point of the isotherm. With the ideal gas state equation ($p = CRT$), Eq. S10 is transformed to the following equation, from which the analysis solution of the LDF model can be obtained:

$$\frac{C-C_\mathrm{e}}{C_0-C_\mathrm{e}}=1-\left(\frac{C_0-C_\mathrm{d}}{C_0-C_\mathrm{e}}\right)\left(\frac{1}{\alpha+1}\right)\left[1-\exp\left(-\left(\frac{\alpha+1}{\alpha}\right)k_\mathrm{s}a_\mathrm{p}t\right)\right] \quad \text{(Eq. S11)}$$

The relationship between each pressure value and the adsorbate density C is expressed by Eqs. S12-S14, respectively,

$$C_0=\frac{p_\mathrm{S,0}V_\mathrm{S}+p_\mathrm{D,d}V_\mathrm{D}}{\left(V_\mathrm{S}+V_\mathrm{D}\right)RT} \quad \text{(Eq. S12)}$$

$$C=\frac{p_\mathrm{D}}{RT} \quad \text{(Eq. S13)}$$

$$C_\mathrm{e}=\frac{p_\mathrm{D,e}}{RT} \quad \text{(Eq. S14)}$$

where $p_\mathrm{S,0}$ (kPa) is the initial pressure of manifold system (S), $p_\mathrm{D,d}$ and p_D (kPa) are the pressure of sample tube (D) before and after vapor dosing, V_D and V_S (mL) are sample tube volume and manifold system volume, respectively. Note that V_S has been measured during instrument manufacturing at 34.257 mL for our instrument, whereas V_D depends on the specific sample tube and the loaded sample amount, with 28 ± 2 mL during our experiments. Considering the small amount of sample involved in the measurement (30~50 mg), the structure transformation caused volume change of $Ni(BIC)_2$ (1.3%) is negligible for the overall volume (< 0.002%). Therefore, the adsorption kinetics of the flexible material could be measured credibly by this volumetric method built into the instrument.

Liquid-phase adsorption equilibria. $Ni(BIC)_2$ (50 mg) samples were evacuated under vacuum at 423 K for 24 h and then immersed in 2 mL varied xylene isomer mixtures at different temperatures for 3 h. Following immersion, the samples were filtered and dried in air at room temperature for 2 h. Subsequently, 10 mg of each sample was digested with 2 mL of 12 M HCl aqueous solution. Xylene in the digestion solution was extracted overnight using 2 mL of chromatographic-grade CH_2Cl_2. The organic phase was then collected for gas chromatography (GC) analyses to determine the molar ratios of the isomers. The overall uptake amount was determined by TG analysis. The selectivity (S_{ij}) is defined by:

$$S_{ij} = \left(y_i / y_j\right) / \left(x_i / x_j\right) \quad \text{(Eq. S15)}$$

where S_{ij} is the liquid-phase adsorption selectivity of component i over j, y_i and y_j are the mole fractions of components i and j in the adsorbed phase, x_i and x_j are the molar fractions of components i and j in the equilibrium liquid phase. The x_i/x_j and y_i/y_j ratios were derived from the peak area ratios of individual components in GC chromatograms. The selectivities were measured in binary mixtures (PX/MX, PX/OX, and PX/EB) with compositions ranging from 0.005/0.995 to 0.9/0.1 at 333 and 393 K, equimolar ternary mixture of PX/MX/OX at 303 to 393 K, quaternary mixture with composition of PX/MX/OX/EB = 22/50/22/6 at 303 to 393 K. The binary liquid-phase adsorption data were further fitted with the Stuart model (*43*). This model assumes that upon reaching equilibrium in the adsorption process, the chemical potentials of each component in the adsorption phase and the main phase are equal. Consequently, the *P*-*V*-*T* properties of the two phases can be described by the van der Waals equation of state. The Stuart model presents a correlating Eq. S16 for liquid-phase adsorption equilibria, which includes the effects of temperature:

$$\log\left(\frac{Y}{1-Y}\right) = \left(1-\frac{\varphi}{T}\right)\left[\log\left(\frac{X}{1-X}\right)\right] + \frac{K}{T} \quad \text{(Eq. S16)}$$

Here, X and Y represent the molar fraction of a component in the main phase and the adsorbed phase, respectively, while φ and K are model parameters. At constant temperature, if let $M = (1-\varphi/T)$, and $N = K/T$, Eq. S16 can be written as:

$$\log\left(\frac{Y}{1-Y}\right) = M\left[\log\left(\frac{X}{1-X}\right)\right] + N \quad \text{(Eq. S17)}$$

Vapor-phase multicomponent breakthrough measurements. Vapor-phase multicomponent breakthrough measurements were carried out with a mixture of xylene isomers in the vapor state as the feed. Typically, the $Ni(BIC)_2$ sample was activated at 423 K for 24 h under an ultrahigh vacuum before measurement. The activated sample was then manually packed into a stainless-steel column (4.6 mm I.D. × 30 mm, containing 0.4 g of the sample) within an N_2-filled glovebox. A stream of nitrogen (99.999% purity) at a flow rate of 15 mL min^{-1} was used to carry the xylene isomers, which were volatilized from bubblers maintained at 298 K. The partial pressure ratios of PX, MX, OX, and EB in the quaternary mixture were set at 22/50/22/6. For ternary and binary

xylene mixtures, equimolar ratios were used. To achieve the desired partial pressure ratio of 22/50/22/6, the saturated pressure of each pure component was calculated by the Antoine equation:

$$\log(P) = \mathrm{A} - \frac{B}{T+C} \tag{Eq. S18}$$

where P (mmHg) is the saturated pressure of pure component, A, B, and C are empirical constants with values listed in Table S14, T (°C) represents the temperature. These saturated pressures provided an initial estimate of the liquid composition, assuming an ideal solution. The vapor-phase ratios were then calibrated by performing a control experiment with an empty column, adjusting the liquid composition in the bubblers iteratively until the desired vapor mixture (22/50/22/6) was detected by GC. Typically, the liquid volumes for the quaternary xylene mixture at 298 K were about 11, 26, 15, and 2.8 mL for PX, MX, OX, and EB, respectively. The total amount of feed liquid is large enough to ensure a negligible vapor constitute change in a single vapor breakthrough experiment. The same iterative approach was adopted to determine the compositions of ternary and binary mixtures. The outlet vapor from the adsorption column was analyzed with a GC-2010 pro gas chromatograph (Shimadzu, Japan) equipped with a flame ionization detector (FID), and a PEG-20M capillary column (50 m length, 0.32 mm internal diameter, 0.5 μm film thickness). The column temperature is 353 K. Following each breakthrough experiment, the sample column was reactivated and regenerated with a N_2 flow at 423 K for 48 h. The adsorption capacity for each vapor component (q_i, mmol g^{-1}) was calculated using the equation:

$$q_i = \frac{\left(Ft - V_{\mathrm{dead}}\right)C_{0,i} - \int_0^t FC_{\mathrm{e},i}\Delta t}{m} \tag{Eq. S19}$$

where F (mL min^{-1}) represents the total flow rate, t (min) is the adsorption time, V_{dead} (mL) is the dead volume of the system (determined using helium), $C_{0,i}$ (mmol mL^{-1}) is the inlet concentration of the component i, $C_{\mathrm{e},i}$ (mmol mL^{-1}) is the effluent concentration of the component i, and m (g) is the mass of the adsorbents. The dynamic adsorption selectivity (S) for a component i relative to component j was then calculated according to the equation:

$$S = \frac{q_i / x_i}{q_j / x_j} \tag{Eq. S20}$$

where x_i is the molar fraction of the component i in the inlet stream.

Single-crystal X-ray diffraction. X-ray diffraction data for the as-synthesized and fully desolvated single crystals of the $Ni(BIC)_2$ were obtained using a D8 Venture single crystal X-ray diffractometer (Bruker, Germany) equipped with Ga Kα radiation (λ = 1.34139 Å) and a PHOTON II detector. The crystal was maintained at 170 K during data collection. The initial structure was solved with Olex2 package (*49*) using the SHELXT (*50*) structure solution program with the Intrinsic Phasing method. Subsequent refinements were performed with the SHELXL (*51*) refinement program using the Least Squares minimization method.

Electron microscopy. The scanning electron microscopy (SEM) images were obtained using an

S-3400 scanning electron microscope (Hitachi, Japan) with an accelerating voltage of 15 kV. For high-resolution transmission electron microscopy (HRTEM) analysis, samples of both pre-adsorption, degassed $Ni(BIC)_2$ (**1**), and post-adsorption $Ni(BIC)_2$ (**2**) were dispersed in an ethanol medium. This process was facilitated by ultrasonication to ensure a uniform suspension. An aliquot of the resultant suspension was then carefully deposited onto a carbon-coated copper grid. The microstructural details and lattice fringes were examined with a JEM-2100F transmission electron microscope (JEOL, Japan) operated at an accelerating voltage of 200 kV.

Structure determination by electron diffraction (ED). For the ED analysis, the samples were activated under vacuum at 423 K for 24 h and dispersed in a selection of adsorbates (PX, EB, MX, or OX) by ultrasonication. The resultant microcrystalline powder was then carefully deposited onto a carbon-coated copper grid. This grid was immediately submerged in liquid nitrogen to preserve the structural integrity of the sample, followed by a transfer into a 2550 cryo-holder (Fishchone, USA) at 77 K. Data acquisition was performed on a 2100-plus TEM (JEOL, Japan), equipped with a MerlinEM direct electron detector. The TEM was operated under a 200 kV accelerating voltage. The tilting range for data collection varied depending on the location of the crystals on the grid, with ED patterns collected at tilting angles ranging from -53.62° to 66°. Each frame was captured with an exposure time of 1 s, resulting in a 1° wedge per frame. The collected data were visualized with the REDp program (*52*) and processed using XDS (*53*), with the assistance of Coeus software suite (ReadCrystal Tech, China). Crystallographic structure models were constructed directly using the SHELXT package (*50*), and the fidelity of these models was corroborated by the congruence of their simulated PXRD patterns with those obtained experimentally.

Convergence tests for cutoff energy and k-point sampling. The convergence behavior of $Ni(BIC)_2$ (**1**) was evaluated as a representative example. For the cutoff-energy test, single-point calculations were performed at a fixed 2 × 2 × 2 k-point mesh with cutoff energies ranging from 400 to 571 eV. For the k-point convergence test, calculations were carried out at a fixed cutoff energy of 544 eV using 1 × 1 × 1, 2 × 2 × 2, and 3 × 3 × 3 k-point meshes. The convergence results are summarized in fig. S40, table S15 and S16. The results indicate that the total energy is effectively converged at a cutoff energy of 544 eV, while the energy difference between the 2 × 2 × 2 and 3 × 3 × 3 k-point meshes is negligible.

Measurements of liquid-phase adsorption isotherms. Liquid-phase adsorption isotherms were measured by following the procedures in (*57, 58*). A stainless-steel chromatographic column (15 cm × 0.46 cm I.D.) was packed with $Ni(BIC)_2$ particles (2.1 g, 300 mesh) and maintained at 393 K, with 1,3,5-trimethylbenzene (TMB) as the mobile phase. The column was initially equilibrated with TMB, and then feed solutions of xylene isomers (3-100 g L^{-1} in TMB) were continuously introduced until adsorption equilibrium was achieved. The column was regenerated by flushing with pure TMB to fully desorb the retained adsorbates. Each adsorption/desorption cycle was maintained for more than 12 h to ensure complete equilibrium and desorption. The desorption eluents were collected and analyzed by gas chromatography (GC). The equilibrium adsorbed

amount of component i was determined as:

$$q_i = \frac{V_{\text{des},i} c_{\text{des},i} - \varepsilon_{\text{t}} V_{\text{col}} c_{\text{feed},i}}{(1-\varepsilon_{\text{t}}) V_{\text{col}}} \quad \text{(Eq. S21)}$$

where q_i is the equilibrium adsorbed concentration of component i on the stationary phase (g L^{-1} adsorbent), $V_{\text{des},i}$ and $c_{\text{des},i}$ are the volume (L) and concentration (g L^{-1}) of the desorption eluent, respectively, $c_{\text{feed},i}$ is the feed concentration (g L^{-1}), V_{col} is the geometric volume (L) of the packed column, and ε_{t} is the total porosity of the column. The adsorption equilibrium data were fitted using the Langmuir isotherm model. For a single-component i:

$$q_i = \frac{q_{m,i} b_i c_i}{1 + b_i c_i} \quad \text{(Eq. S22)}$$

For a multicomponent mixture (extended Langmuir model):

$$q_i = \frac{q_{m,i} b_i c_i}{1 + \sum_j b_j c_j} \quad \text{(Eq. S23)}$$

where q_{i} is the equilibrium adsorbed concentration of component i (g L^{-1} adsorbent), q_{m} is the saturation capacity of component i (g L^{-1} adsorbent), b_i is Langmuir equilibrium constant of component i (L g^{-1}), c_i is the equilibrium liquid-phase concentration of component i (g L^{-1}).

Measurement of mass-transfer kinetics by pulse chromatography. The pulse chromatography was performed on a $Ni(BIC)_2$-packed column (25 cm × 0.46 cm I.D., 300 mesh) using a Quik-sep system (H&E Co., Ltd., China) equipped with a refractive index detector. The column was flushed with chromatographic-grade 1,3,5-trimethylbenzene (TMB) at 1 mL min^{-1} under 393 K until the detector baseline stabilized. Subsequently, 10 µL aliquots of xylene isomers (PX, MX, OX, EB) were injected sequentially via a manual injector, with the flow-rate of the mobile phase maintained at 1 mL min^{-1} throughout the process. Triisopropylbenzene was used as the dead-time marker. The bed porosity (external porosity, ε_b) of the chromatographic column was obtained from the retention time (t_0) of a non-retained tracer (triisopropylbenzene), using the following equation,

$$t_0 = \varepsilon_{\text{b}} \frac{L}{u} \quad \text{(Eq. S24)}$$

where u is the velocity of mobile phase (cm min^{-1}), t_0 is the retention time of triisopropylbenzene (min), ε_{b} is the bed porosity of the packed column, and L is the column length (cm). The internal porosity (ε_{p}) was determined from CO_2 adsorption at 195 K using a Micromeritics ASAP 2020 volumetric adsorption analyzer. The corresponding internal pore volume (V_{p}) was measured to be 0.152 cm^3 g^{-1}.

$$\varepsilon_{\text{p}} = \frac{V_{\text{p}} m_{\text{ads}}}{(1-\varepsilon_{\text{b}}) V_{\text{col}}} \quad \text{(Eq. S25)}$$

The bed porosity (ε_{b}) and total porosity (ε_{t}) are related by Eq. S26,

$$\varepsilon_{\mathrm{t}} = \varepsilon_{\mathrm{b}} + \left(1-\varepsilon_{\mathrm{b}}\right)\varepsilon_{\mathrm{p}} \quad \text{(Eq. S26)}$$

where ε_p is the internal porosity, ε_b is the external porosity, V_p is the internal pore volume determined from the CO_2 isotherm ($cm^3\ g^{-1}$), m_{ads} is the weight of adsorbent (g), and V_{col} is the geometric column volume (cm^3). Kinetic parameters were extracted by fitting experimental pulse-response data to a 1D axial-dispersion model coupled with linear driving force (LDF) mass transfer kinetics (*60, 61*):

$$\varepsilon \frac{\partial c(z,t)}{\partial t} = D_{\mathrm{ax}} \frac{\partial^2 c}{\partial z^2} - u\frac{\partial c}{\partial z} - \left(1-\varepsilon_b\right) MTC\left(q(c) - q_{\mathrm{s}}(z,t)\right) \quad \text{(Eq. S27)}$$

where $c(z,t)$ is the solute concentration in the mobile phase ($g \cdot L^{-1}$), $q(z,t)$ is the adsorbed phase concentration ($g \cdot L^{-1}$ adsorbent), ε_b is the bed porosity, u is the interstitial velocity defined as $u = Q/(A\varepsilon_b)$ ($cm \cdot min^{-1}$), D_{ax} is the axial dispersion coefficient ($cm^2 \cdot min^{-1}$), and MTC is the overall mass-transfer coefficient (min^{-1}). The equilibrium loading $q(c)$ follows the Langmuir isotherm in Eq. S22. The inlet concentration was introduced as a finite square pulse:

$$c_{\mathrm{in}}(t) = \begin{cases} 1, 0 \le t \le \tau \\ 0, t > \tau \end{cases} \quad \text{(Eq. S28)}$$

with pulse duration τ (min) and pulse concentration c_0. Initially, $c(z,0) = 0$ and $q_s(z,0) = 0$; appropriate boundary conditions (inlet concentration history and zero-gradient or convective outlet) were applied during numerical solution. Numerical solutions of the governing partial differential equations were obtained using a finite difference method with N_x spatial node along the column length L. Central differencing was employed for both convection and dispersion terms, while integration in time used an explicit fourth-order Runge-Kutta method with adaptive time-stepping for numerical stability. Computations were performed using Python scripts implementing the numerical procedures described above. Physically meaningful parameter ranges were specified during parameter optimization to avoid nonphysical solutions and improve numerical stability. Kinetic parameters were determined by best fitting of the experimental data.

Mathematical modeling of SMB. A four-zone simulated moving bed (SMB) process was simulated in Aspen Chromatography to evaluate the separation performance, using 24 columns for FAU zeolite and $Ni(BIC)_2$. Aspen solves the coupled set of partial differential equations (mass, momentum, and energy balances) by spatial discretization (*62-64*). The Upwind Differencing Scheme 1 (UDS1) with 20 spatial nodes was selected as a compromise between numerical accuracy and simulation time, and time integration was performed with an implicit Euler method. A lumped-pore model was used to describe intraparticle transport, with axial dispersion and mass-transfer resistance included. The component mass balance in the bulk mobile phase for component i in column k is:

$$\frac{\partial c_{i,k}}{\partial t} + u_{\mathrm{int},k}\frac{\partial c_{i,k}}{\partial x} + \frac{(1-\varepsilon)}{\varepsilon}\left[\varepsilon_{\mathrm{p}}\frac{\partial c_{\mathrm{p},i,k}}{\partial t} + \left(1-\varepsilon_{\mathrm{p}}\right)\frac{\partial q_{\mathrm{i,k}}}{\partial t}\right] = D_{\mathrm{ax},i}\frac{\partial^2 c_{i,k}}{\partial x^2} \quad \text{(Eq. S29)}$$

where i indexes components (e.g., A, B), k is the column number, $c_{i,k}$ (g L^{-1}) is the concentration of component i in the mobile phase in column k, $c_{\mathrm{p},i,k}$ (g L^{-1}) is the average concentration in the particle pore phase, $q_{i,k}$ (g L^{-1} adsorbent) is the average solid-phase loading in local equilibrium with $c_{\mathrm{p},i,k}$, $u_{\mathrm{int},k}$ (cm min^{-1}) is the interstitial velocity, $D_{\mathrm{ax},i}$ (cm^2 min^{-1}) is the axial dispersion coefficient, ε is the bed porosity, ε_p is the internal porosity, x (cm) and t (min) are space and time coordinates, respectively. Mass transfer within the particle was represented by the linear driving-force (LDF) relation in lumped form:

$$\varepsilon_\mathrm{p}\frac{\partial c_{\mathrm{p},i,k}}{\partial_t}+\left(1-\varepsilon_\mathrm{p}\right)\frac{\partial q_{i,k}}{\partial t}=MTC_i\frac{3}{r_\mathrm{p}}\left(c_{i,k}-c_{\mathrm{p},i,k}\right) \quad \text{(Eq. S30)}$$

where r_p (cm) is the average particle radius of the adsorbent, MTC_i (cm min^{-1}) is the mass-transfer coefficient that lumps internal and external resistances. Initial conditions for each column were:

$$t=0,\ c_{i,k}=q_{i,k}=c_{\mathrm{p},i,k}=0 \quad \text{(Eq. S31)}$$

Danckwerts boundary conditions were applied at each column inlet and outlet:

$$x=0, D_{\mathrm{ax},i}\frac{\partial c_{i,k}}{\partial x}=u_{\mathrm{int},k}\left(c_{i,k}-c_{i,k}^{\mathrm{in}}\right) \quad \text{(Eq. S32)}$$

$$x=L,\frac{\partial \mathrm{c}_{i,k}}{\partial_x}=0 \quad \text{(Eq. S33)}$$

where the superscript "in" denotes the inlet of streams, and L (m) is the column length. Node (stream) balances relate section flow rates and concentrations at the interconnections (desorbent, extract, feed, raffinate nodes). For the desorbent node:

$$Q_1=Q_D+Q_4 \quad \text{(Eq. S34)}$$

$$c_{i,k+1}^{\mathrm{in}}=\frac{Q_4c_{i,k}^{\mathrm{out}}}{Q_1} \quad \text{(Eq. S35)}$$

For the extract node:

$$Q_2=Q_1-Q_\mathrm{E} \quad \text{(Eq. S36)}$$

$$c_{i,k+1}^{\mathrm{in}}=c_{i,k}^{\mathrm{out}} \quad \text{(Eq. S37)}$$

For the feed node:

$$Q_3=Q_2+Q_\mathrm{F} \quad \text{(Eq. S38)}$$

$$c_{i,k+1}^{\mathrm{in}}=\frac{Q_\mathrm{F}c_{\mathrm{feed},i}+Q_2c_{i,k}^{\mathrm{out}}}{Q_3} \quad \text{(Eq. S39)}$$

For the raffinate node:

$$Q_4 = Q_3 - Q_R \quad \text{(Eq. S40)}$$

$$c_{i,k+1}^{\text{in}} = c_{i,k}^{\text{out}} \quad \text{(Eq. S41)}$$

Here, $c_{\text{feed},i}$ (g L^{-1}) is the feed concentration, Q_1 (m^3 h^{-1}), Q_2 (m^3 h^{-1}), Q_3 (m^3 h^{-1}), and Q_4 (m^3 h^{-1}) are the section flow rates, and Q_R (m^3 h^{-1}), Q_E (m^3 h^{-1}), Q_D (m^3 h^{-1}), and Q_F (m^3 h^{-1}) are the raffinate, extract, desorbent, and feed flow rates, respectively. Superscript "out" denote the outlet concentration of the respective stream.

Triangle theory. Triangle theory (equilibrium theory) provides a simple, widely used map for selecting SMB operating conditions (sectional flow rates and switching time) by neglecting axial dispersion and mass-transfer resistances (*65*). Under this theory the operating stage is represented in terms of the dimensionless section flow rates m_j (j = 1-4), which are related to the actual sectional flow rates Q_j^{SMB} and switching time t_s by:

$$m_j = \frac{Q_j^{\text{SMB}} t_s - V_{\text{col}} \varepsilon_t}{V_{\text{col}} (1 - \varepsilon_t)} \quad \text{(Eq. S42)}$$

where Q_j^{SMB} (m^3 h^{-1}) is the volumetric flow rate of section j, t_s (min) is the switching time, V_{col} (mL) is the column geometric volume, m_j is dimensionless flow rate in section j, and ε_t is the total column porosity.

In this work, the feed consisted of four xylene isomers (PX, MX, OX, and EB) and the desorbent, and the multicomponent system was simplified into a pseudo-binary form by grouping the components according to their adsorption strength (*66*). PX, which was the most strongly adsorbed species, was treated as the strong-key component (A). Among the remaining isomers, EB exhibited the highest adsorption strength and was therefore selected as the representative weak-key component (B). The overall feed concentration of component B was defined as the sum of the feed concentrations of MX, OX, and EB ($c_B = c_{MX} + c_{OX} + c_{EB}$). The adsorption parameters of component B, including the Langmuir affinity constant (b_B) and the Henry constant (H_B), were assigned to those of EB, while the saturation capacities of A and B were assumed to be equal. This conservative pseudo-binary treatment enables the multicomponent equilibrium behavior to be described within the same analytical framework as the classical binary triangle theory and ensures that the predicted pseudo-binary separation region remains enclosed within the true four-component separation region.

For a pseudo-binary feed (components A and B; A = more strongly retained, B = less retained) and a nonlinear (Langmuir) isotherm, the boundaries delimiting complete binary separation in the (m2, m3) plane are given by the following relations. Define H_i as the Henry-region distribution coefficient of component i (i = A, B); for a Langmuir isotherm $H_i = q_{m,i} \cdot b_i$ (where $q_{m,i}$ and b_i are the Langmuir saturation capacity and affinity constant, respectively). Let c_A and c_B be the feed concentrations of components A and B, respectively, and b_A, b_B be the Langmuir affinity constants: Forward boundary (line *wf*):

$$[H_{\mathrm{A}} - \omega_1(1+b_{\mathrm{A}}c_{\mathrm{A}})]m_2 + b_{\mathrm{A}}c_{\mathrm{A}}\omega_1 m_3 = \omega_1(H_{\mathrm{A}} - \omega_1) \quad \text{(Eq. S43)}$$

Backward boundary (line *wb*):

$$[H_{\mathrm{A}} - H_{\mathrm{B}}(1+b_{\mathrm{A}}c_{\mathrm{A}})]m_2 + b_{\mathrm{A}}c_{\mathrm{A}}H_{\mathrm{A}} m_3 = H_{\mathrm{B}}(H_{\mathrm{A}} - H_{\mathrm{B}}) \quad \text{(Eq. S44)}$$

Radical/curved boundary (curve *ra*):

$$m_3 = m_2 + \frac{\left(\sqrt{H_{\mathrm{A}}} - \sqrt{m_2}\right)^2}{b_{\mathrm{A}}c_{\mathrm{A}}} \quad \text{(Eq. S45)}$$

Diagonal line (*ab*):

$$m_3 = m_2 \quad \text{(Eq. S46)}$$

The intersection points of these curves are as follows:

Point *a*: (H_{A}, H_{A})

Point *b*: (H_{B}, H_{B})

Point f: (ω_1, ω_1)

Point *r*: $\left(\frac{\omega_1^2}{H_{\mathrm{A}}}, \frac{\omega_1\left[\omega_2\left(H_{\mathrm{A}}-\omega_1\right)\left(H_{\mathrm{A}}-H_{\mathrm{B}}\right)+H_{\mathrm{B}}\omega_1\left(H_{\mathrm{A}}-\omega_2\right)\right]}{H_{\mathrm{A}}H_{\mathrm{B}}\left(H_{\mathrm{A}}-\omega_2\right)}\right)$

Point *w*: $\left(\frac{H_{\mathrm{B}}\omega_1^2}{H_{\mathrm{A}}}, \frac{\omega_1\left[\omega_2\left(H_{\mathrm{A}}-H_{\mathrm{B}}\right)+H_{\mathrm{B}}\left(H_{\mathrm{B}}-\omega_2\right)\right]}{H_{\mathrm{B}}\left(H_{\mathrm{A}}-\omega_2\right)}\right)$

with $\omega_1 > \omega_2 > 0$, which are parameters determined as the two roots of the quadratic equation:

$$\left(1+b_{\mathrm{A}}c_A+b_{\mathrm{B}}c_B\right)\omega^2 - \left[H_{\mathrm{A}}\left(1+b_{\mathrm{B}}c_B\right)+H_{\mathrm{B}}\left(1+b_{\mathrm{A}}c_A\right)\right]\omega + H_{\mathrm{A}}H_{\mathrm{B}} = 0 \quad \text{(Eq. S47)}$$

The subscripts "A" and "B" represent the more retained component (A, here PX) and the less retained component (B, here MX, OX, EB), respectively.

SMB performance metrics. The performance of SMB process was evaluated using five key indicators at steady state including relative purity, recovery, productivity, solvent consumption, and processing capacity (*PC*, m^3 xylene $h^{-1}\cdot m^{-3}$). All metrics are evaluated using steady-state (periodic) averages over one switching interval (i.e., the mean concentrations/flows during one switch period after the SMB reaches cyclic steady state).

1) Relative purity (*P*, %) of PX in the extract is the fraction of PX mass concentration in the extract stream:

$$P_{\mathrm{PX}} = \frac{C_{\mathrm{E,PX}}}{C_{\mathrm{E,PX}} + C_{\mathrm{E,MX}} + C_{\mathrm{E,OX}} + C_{\mathrm{E,EB}}} \times 100\% \quad \text{(Eq. S48)}$$

where $C_{\mathrm{E},i}$ is the average concentration of component *i* in the extract stream (g L^{-1}).

2) Recovery (*Y*, %) of PX is the fraction of the total PX fed that is recovered in the extract

over one switching interval:

$$Y_{\mathrm{PX}} = \frac{Q_{\mathrm{E}} C_{\mathrm{E,B}}}{Q_{\mathrm{F}} C_{\mathrm{F,B}}} \times 100\% \tag{Eq. S49}$$

where Q_E and Q_F are the volumetric flow rates of the extract and feed (m^3 h^{-1}), respectively, and $C_{E,PX}$ and $C_{F,PX}$ are the concentrations of PX in the extract and feed (g L^{-1}).

3) Productivity (PR, g PX L^{-1} adsorbent h^{-1}) is product mass per unit adsorbent volume per hour:

$$PR = \frac{1000 \times \left(C_{\mathrm{E,PX}} Q_{\mathrm{E}} \right)}{n \times V_{\mathrm{col}} \left(1 - \varepsilon_{\mathrm{t}} \right)} \tag{Eq. S50}$$

where $C_{E,PX}$ is the PX concentration in the extract (g L^{-1}), Q_E is the extract flow rate (m^3 h^{-1}), n is the number of columns, V_{col} is the single-column volume (L), and ε_t is the total porosity.

4) Solvent consumption (SC, L g^{-1}) is the volume of desorbent required to produce one unit mass of product.

$$SC = \frac{Q_{\mathrm{D}}}{C_{\mathrm{E,PX}} Q_{\mathrm{E}}} \tag{Eq. S51}$$

where Q_D is the desorbent flow rate (m^3 h^{-1}), Q_E is the extract flow rate (m^3 h^{-1}), and $C_{E,PX}$ is the PX concentration in the extract stream (g L^{-1}).

5) Processing capacity (PC, m^3 feed h^{-1} m^{-3}) is feed volume processed per unit adsorbent volume per hour:

$$PC = \frac{1000 Q_{\mathrm{F}}}{n \times V_{\mathrm{col}} \left(1 - \varepsilon_{\mathrm{t}} \right)} \tag{Eq. S52}$$

where Q_F is the feed flow rate (m^3 h^{-1}).

Distillation process simulation for PX/PDEB in Aspen Plus. Steady-state distillation simulations were carried out in Aspen Plus V11 using the RadFrac rigorous column model to separate a binary mixture of para-xylene (PX) and para-diethylbenzene (PDEB). The model simultaneously solves the material, equilibrium, summation, and energy (MESH) equations for each theoretical stage to obtain vapor-liquid equilibrium compositions, temperatures, and phase flow rates. The NRTL thermodynamic model (*67*) was selected to describe non-ideal vapor-liquid equilibrium behavior, and binary interaction parameters were adopted from the Aspen physical property database. Feed composition, temperature, and pressure were specified according to the extract stream obtained from the upstream SMB process. The feed was introduced at an appropriate stage within the column, and the total condenser and kettle reboiler were used to ensure complete condensation and vaporization at the respective ends. Initial estimates for the number of theoretical stages, reflux ratio, and feed stage location were obtained from shortcut (DSTWU) column calculations, and these values were subsequently refined in the RadFrac model. A design specification (Design Spec) was defined to achieve a target PX purity (99.9%) in the distillate

stream, with either the reflux ratio or distillate flow rate assigned as the manipulated variable. The column pressure profile was adjusted to match realistic industrial operating conditions. Convergence was achieved using the Newton-Raphson algorithm with simultaneous heat and mass balance iterations until the material and energy residuals were below 10^{-6}. Key output parameters, including distillate and bottoms flow rates, product purities, reboiler and condenser duties, internal temperature profiles, and vapor/liquid flow distributions, were extracted for analysis. The specific energy consumption (GJ t^{-1} PX) was calculated by normalizing the total heat duty with respect to the annual PX production rate. Sensitivity analyses were performed by varying the feed stage location, reflux ratio, and column pressure to identify the optimal separation conditions that minimized energy consumption while maintaining product purity above 99.9%.

Stability tests. The as-synthesized $Ni(BIC)_2$ powder samples were activated under vacuum at 423 K for 24 h to ensure the remove of all water molecules. To evaluate the stability during PX adsorption, approximately 0.3 g of this activated $Ni(BIC)_2$ powder was immersed in 10 mL of pure PX or quaternary mixture of PX/MX/OX/EB (22/50/22/6). This setup was maintained at 393 K over two separate durations: 24 h and one week, to assess the short-term and extended stability, respectively. In parallel, the robustness of $Ni(BIC)_2$ against atmospheric moisture was scrutinized. Samples weighing 0.3 g were exposed to ambient air with a relative humidity of approximately 30% over an extended period of three months. After these treatments, the structural integrity of the samples was examined through powder X-ray diffraction (PXRD) to detect any degradation or structural changes.

Recyclability tests. To ascertain the reusability of $Ni(BIC)_2$ in the adsorption of PX, a series of adsorption-desorption cycles was conducted using an SDT 650 simultaneous thermal analyzer (TA Instruments, USA). In these tests, a sample of approximately 5 mg of activated $Ni(BIC)_2$ was placed in the sample chamber of the analyzer under a nitrogen atmosphere at 393 K. A nitrogen flow, saturated with PX vapor at a rate of 50 mL min^{-1}, was introduced to facilitate the adsorption process. The mass uptake was monitored until the adsorption equilibrium was reached. Subsequently, the pure nitrogen flow was used to remove PX, and the temperature was elevated to 453 K and maintained for 2 h to ensure complete desorption. The system was then returned to 393 K before commencing the next cycle. Parallel evaluations were performed in the liquid phase to further investigate $Ni(BIC)_2$ recyclability. Freshly activated samples, weighing 500 mg, were soaked in 10 mL of liquid PX at 393 K for a 3-hour adsorption period. Following adsorption, the samples underwent filtration at the same temperature and were left to dry on filter paper for 3 to 4 h to eliminate any surface-bound PX. The PX-loaded material was then transferred to a tube furnace under nitrogen, heated gradually to 423 K at a rate of 10 K min^{-1}, and held at this temperature for 4 h to desorb PX completely. This procedure was repeated for 10 cycles, with the mass fraction of adsorbed PX measured via TG analysis using a small aliquot from each cycle. Long-term stability and the effect of impurities on the performance of $Ni(BIC)_2$ were also scrutinized. In these tests, 2 g of the activated sample was immersed in a hexanary mixture with a weight ratio of PX/MX/OX/EB/Tol/pDEB = 11/25/11/3/0.75/49.25 at 393 K for 3 h. After the

designated adsorption time, the material was filtered, reactivated under a nitrogen stream at 423 K for 4 h, and then redeployed in subsequent cycles. The adsorption capacity of the samples was determined by TG analysis for each cycle. In contrast, the adsorption selectivity was periodically evaluated by GC analysis of the digested sample after the 1st, 10th, 20th, and 30th cycles.

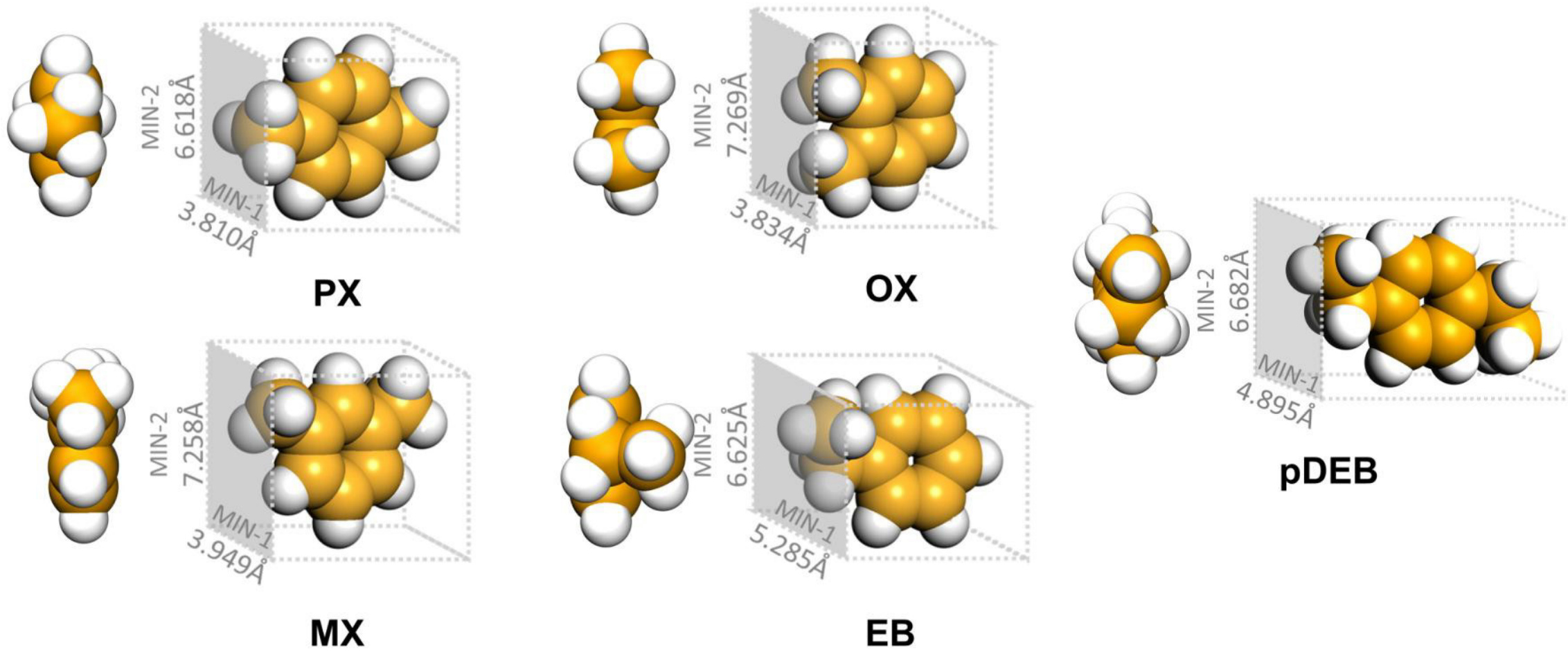

Fig. S1. Molecular projections and structural comparison of xylene isomers and pDEB (MIN-1 and MIN-2).

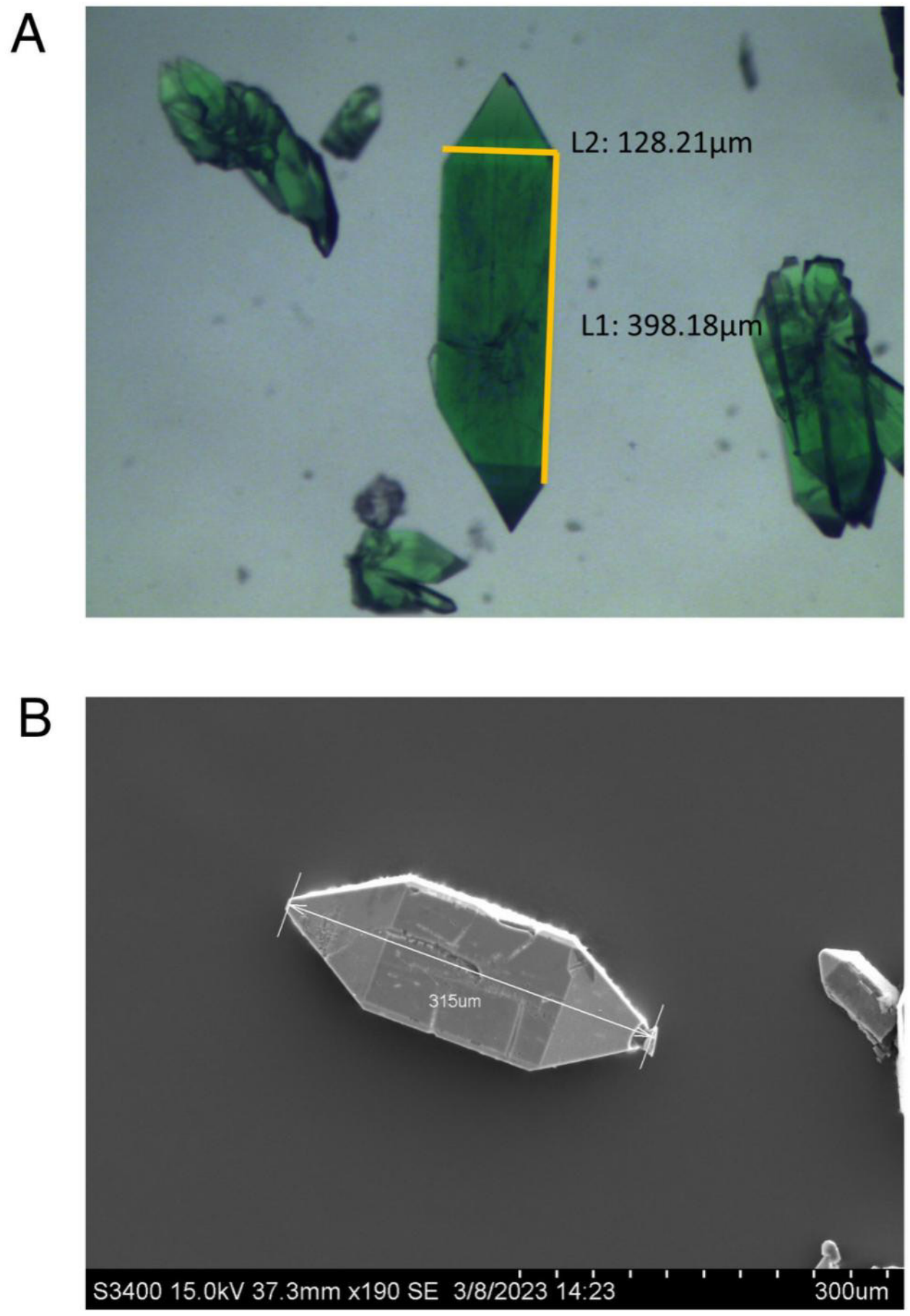

Fig. S2. Image of Ni(BIC)$_2$. (A) Optical microscope image and (B) SEM image of Ni(BIC)$_2$.

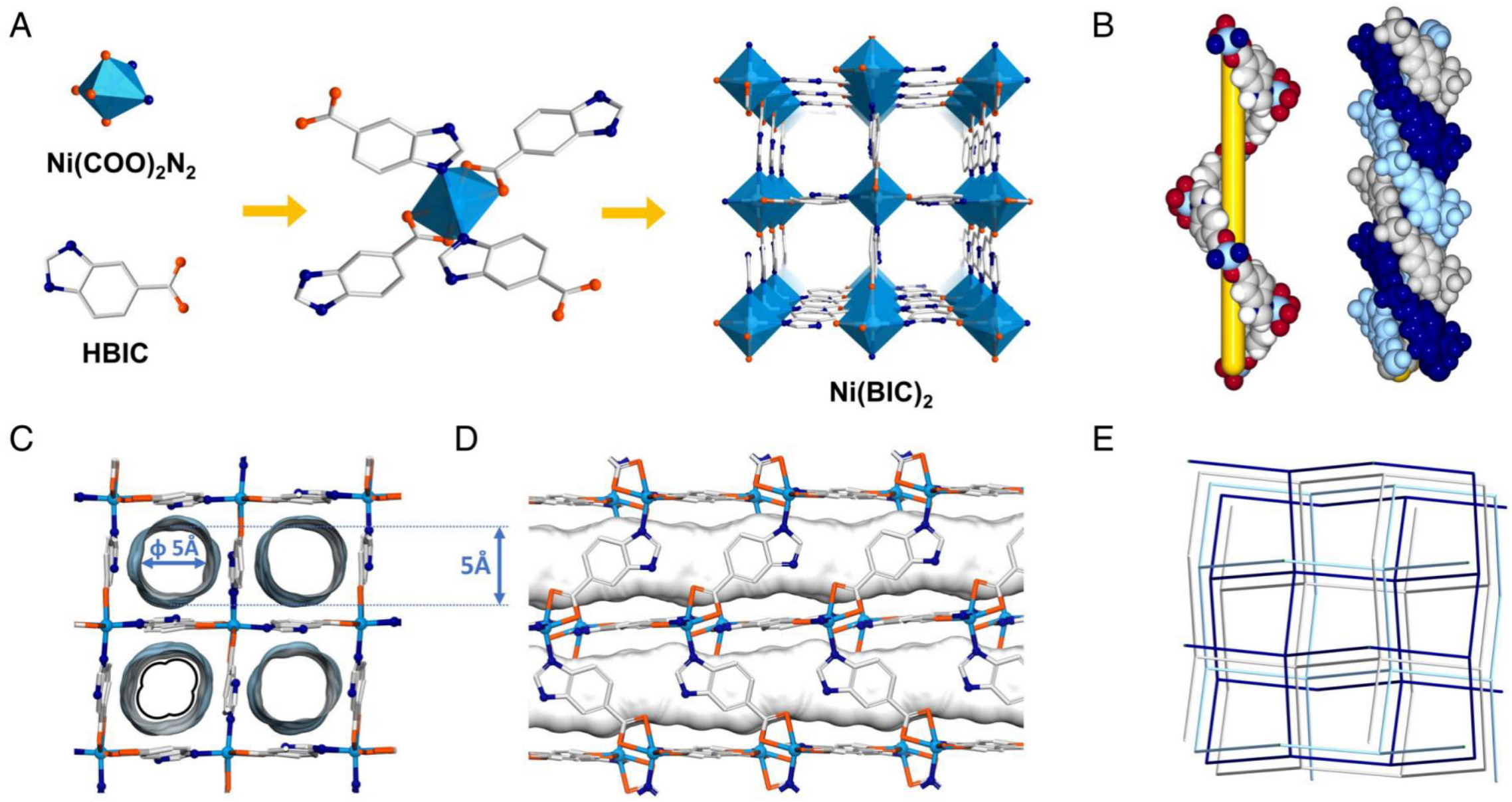

Fig. S3. Detailed depiction of the crystallographic structure of Ni(BIC)$_2$. (A) A view of the crystal structure of Ni(BIC)$_2$ (Color code: Ni, blue; C, grey; O, orange; N, dark blue; H, white). (B) Visualization of a helical chain and triple helices in the structure of Ni(BIC)$_2$. (C) Connolly surface of Ni(BIC)$_2$ on the *ab* plane with a probe radius of 1 Å. (D) Connolly surface of Ni(BIC)$_2$ on the side plane, also with a probe radius of 1 Å. (E) Simplifications of the ***dia*** nets for Ni(BIC)$_2$.

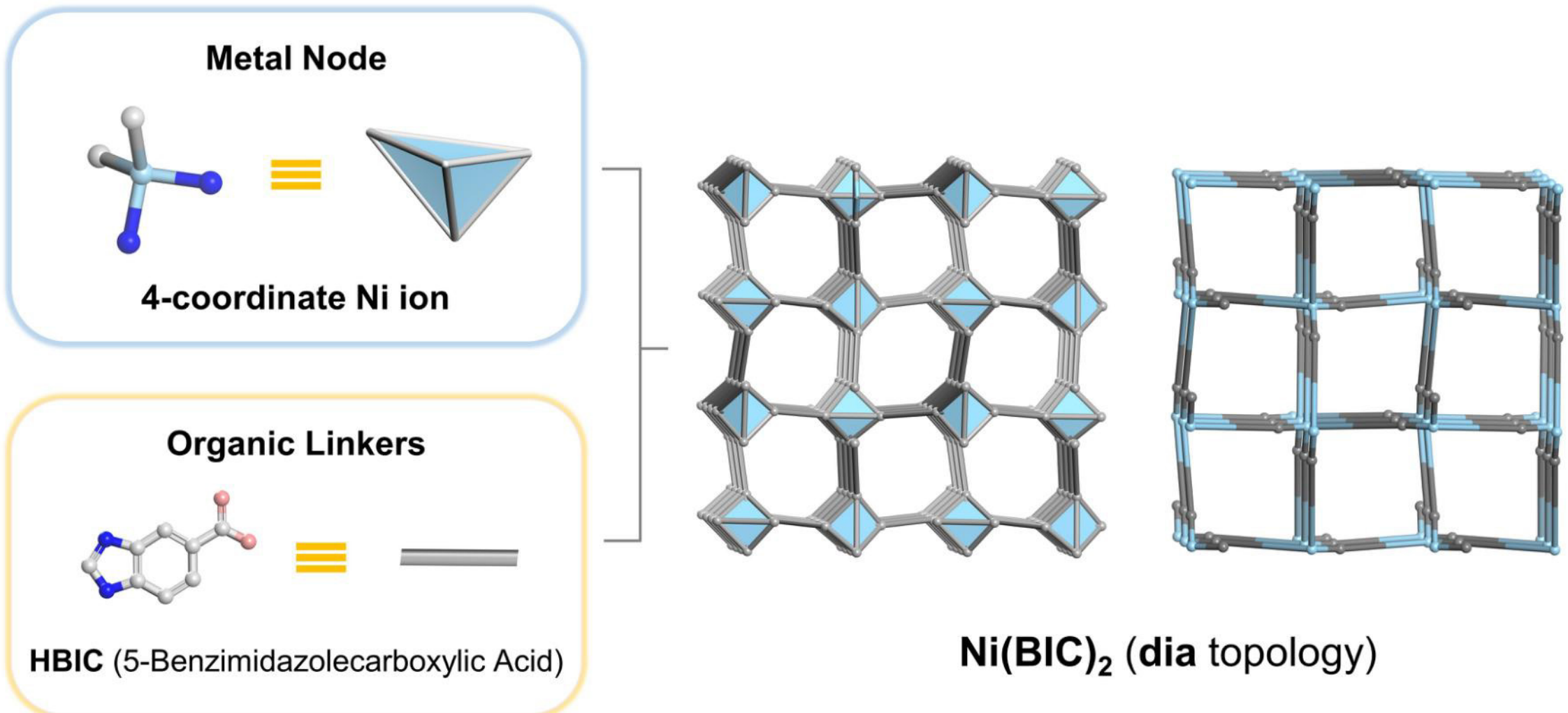

Fig. S4. Representation of the topological structure of $Ni(BIC)_2$.

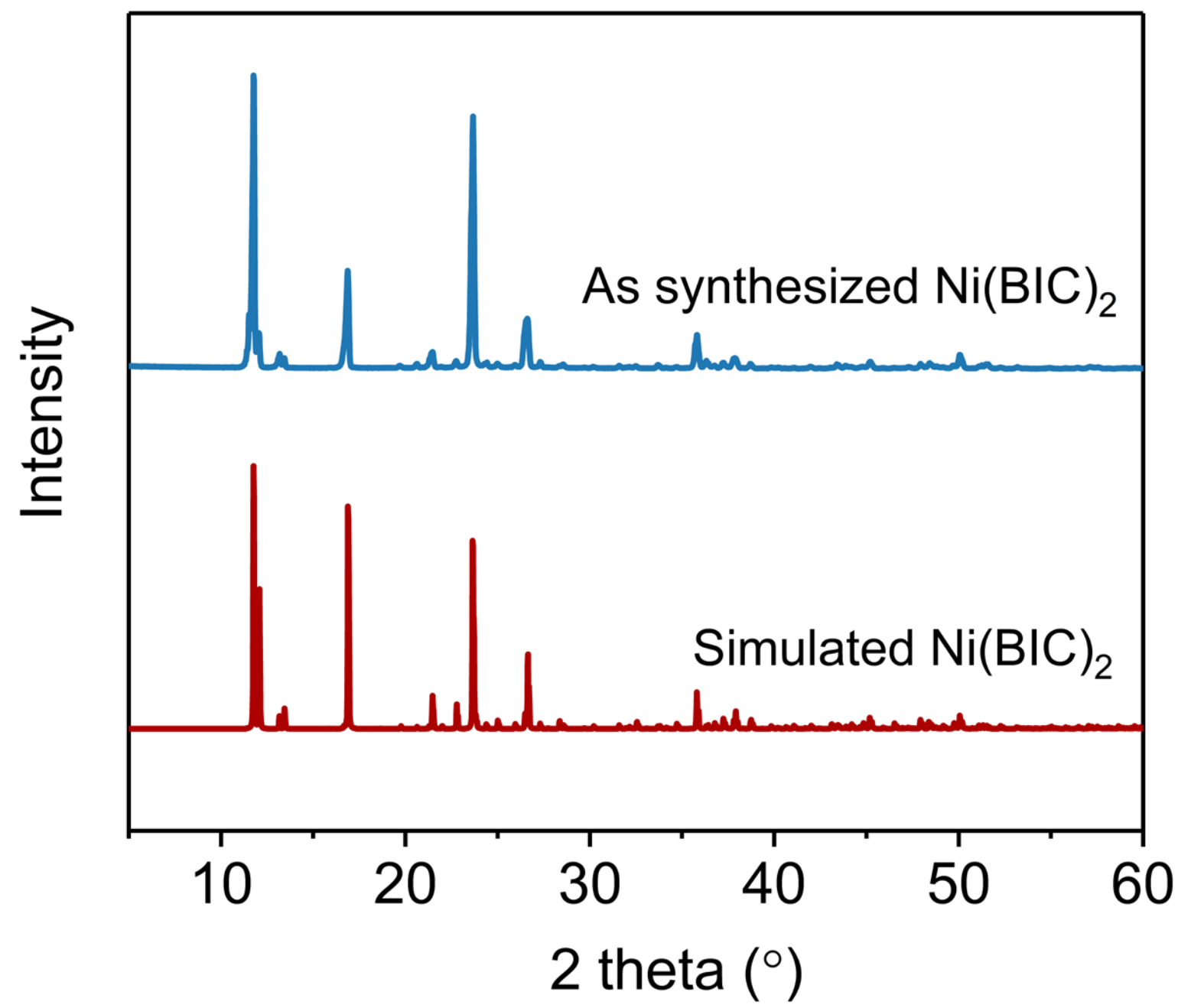

Fig. S5. Comparative PXRD patterns of the simulated $Ni(BIC)_2$ (red) and the as-synthesized $Ni(BIC)_2$ (blue).

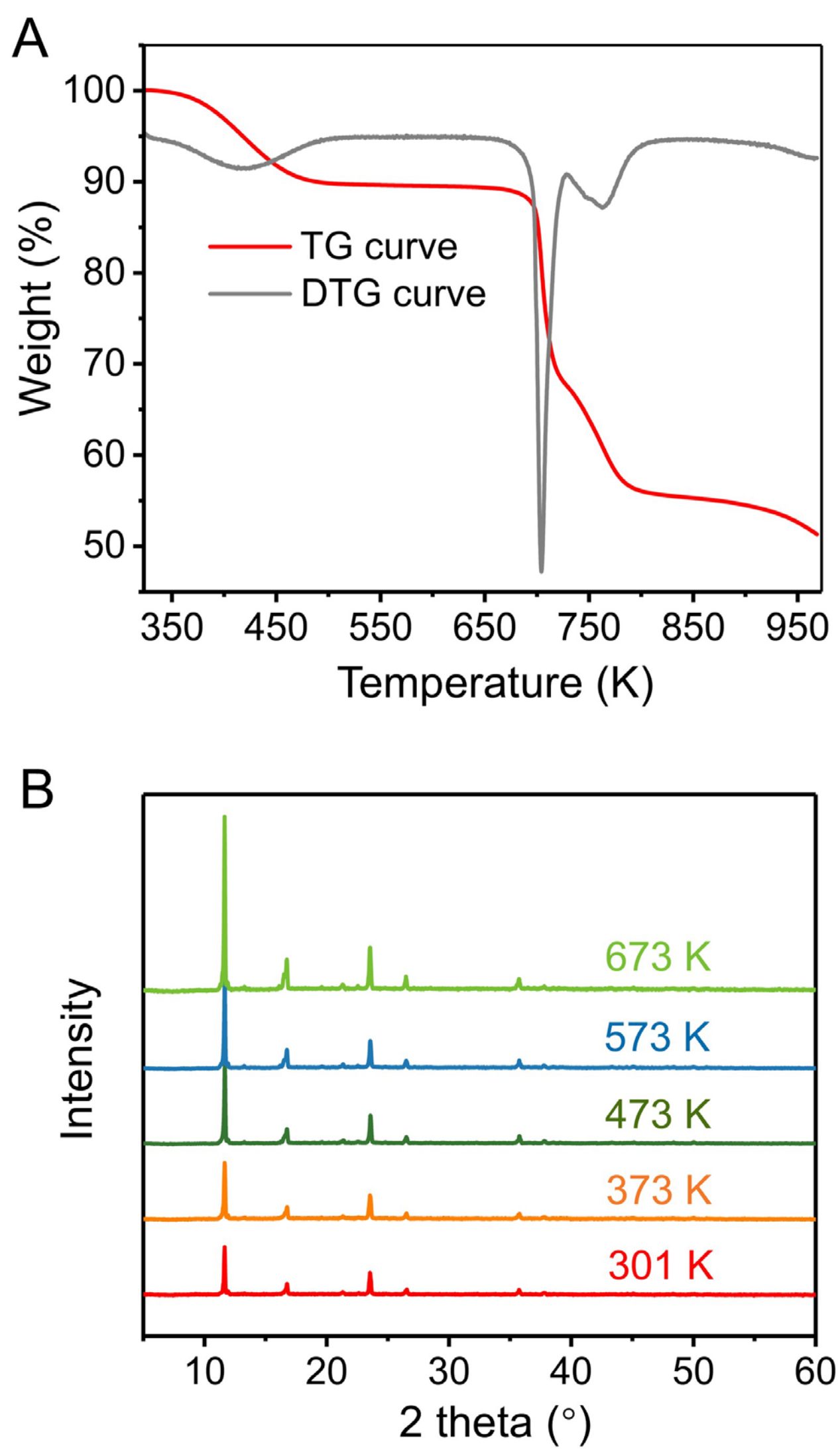

Fig. S6. Thermostability of Ni(BIC)$_2$. (A) TG and DTG curves for Ni(BIC)$_2$. (B) In-situ variable-temperature PXRD patterns of Ni(BIC)$_2$ under a N_2 atmosphere.

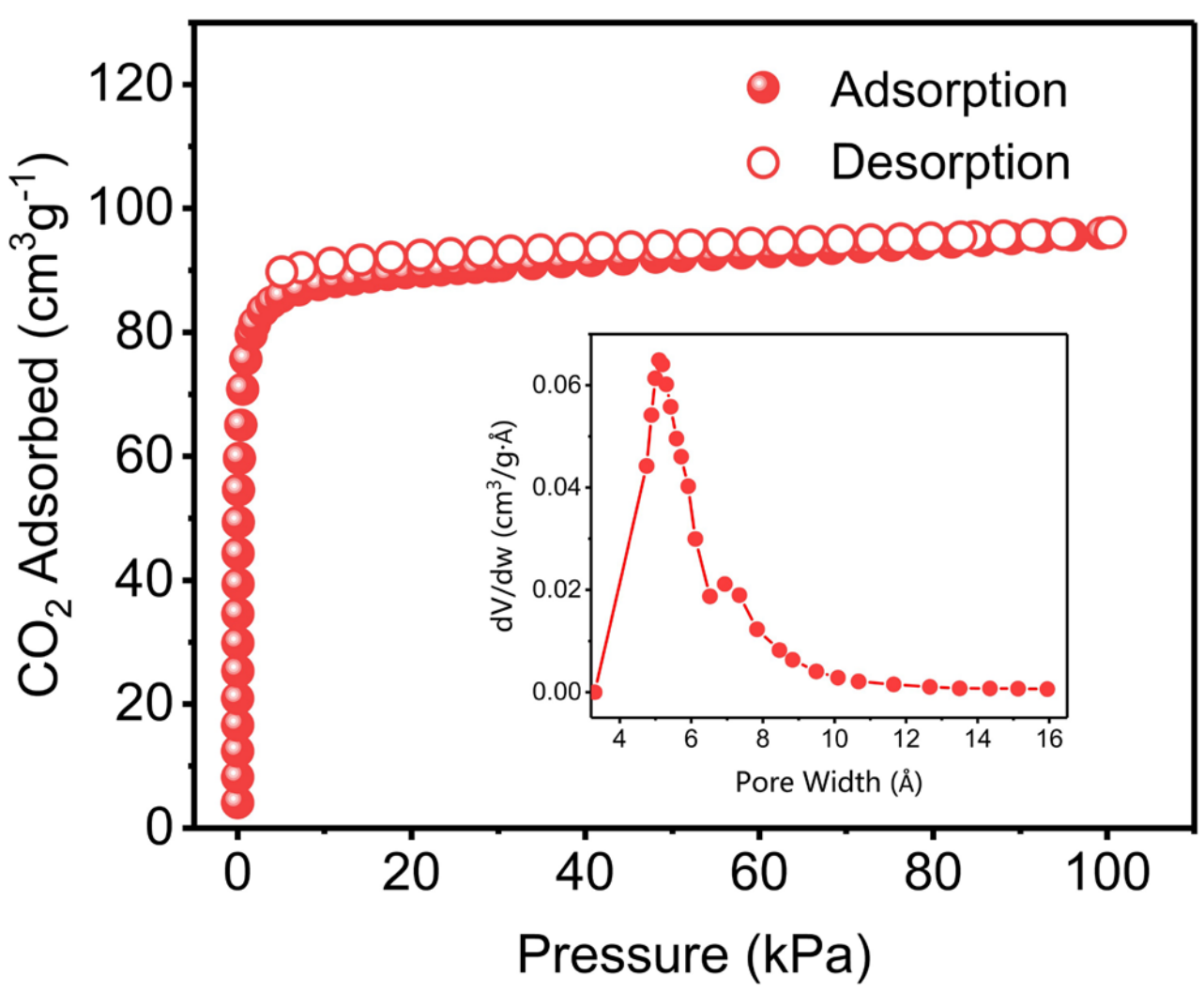

Fig. S7. CO_2 adsorption-desorption isotherms at 195 K on $Ni(BIC)_2$ (Inset graph shows the pore size distribution).

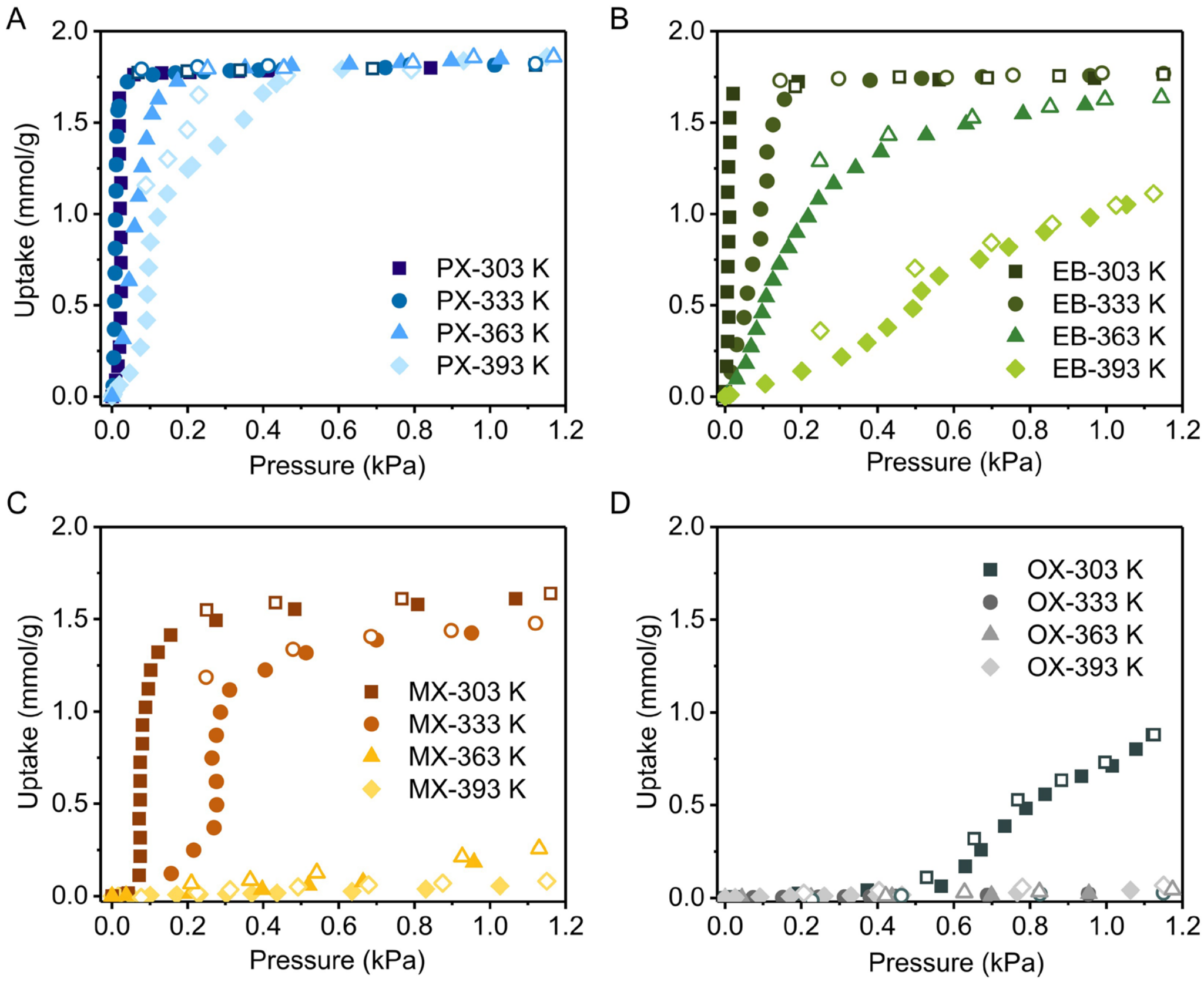

Fig. S8. Single-component adsorption isotherms of (A) PX, (B) EB, (C) MX, and (D) OX vapors on $Ni(BIC)_2$ at 303, 333, 363, and 393 K.

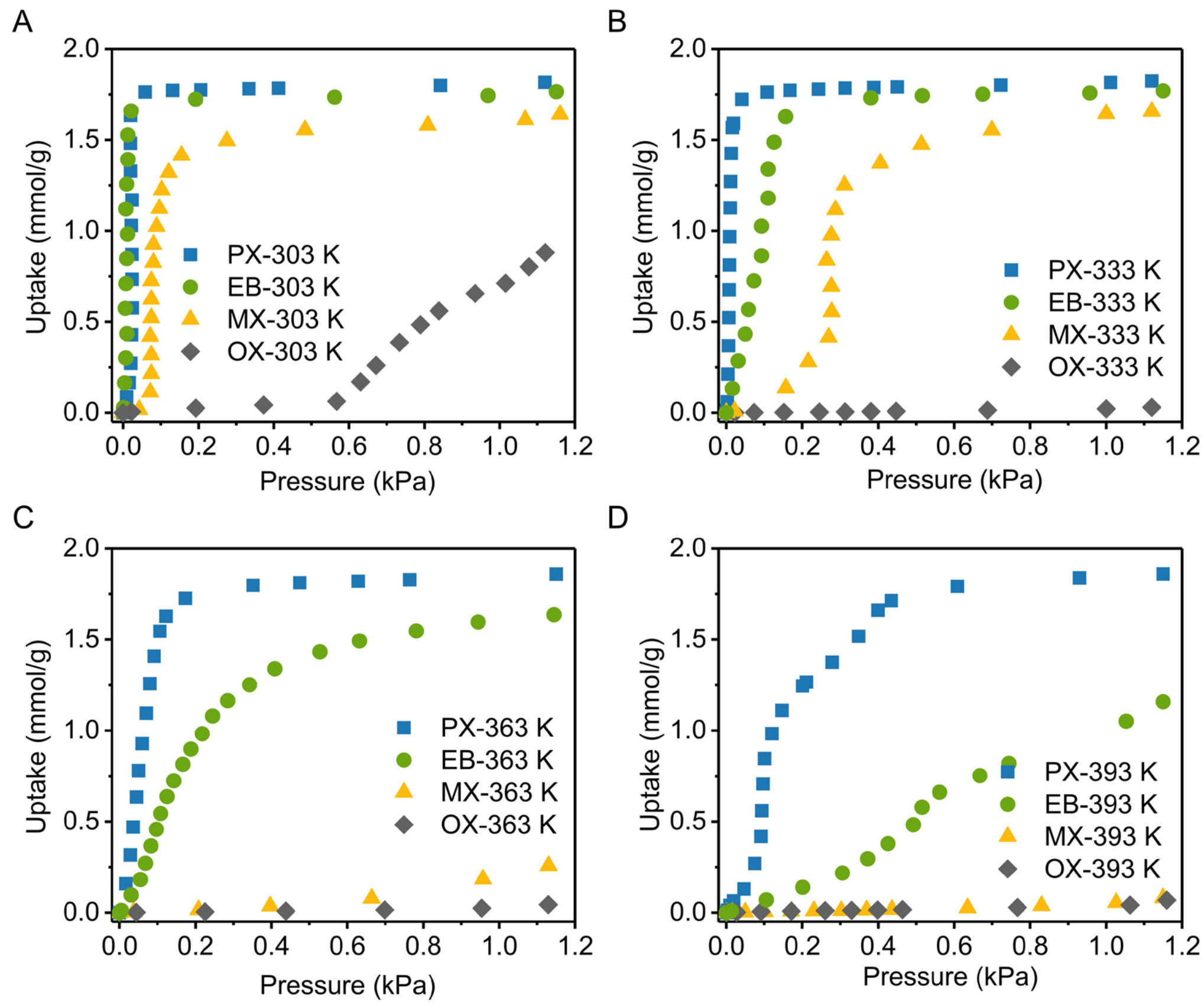

Fig. S9. Combined presentation of single-component adsorption isotherms for PX, MX, OX, and EB vapors on $Ni(BIC)_2$ at (A) 303, (B) 333, (C) 363, and (D) 393 K.

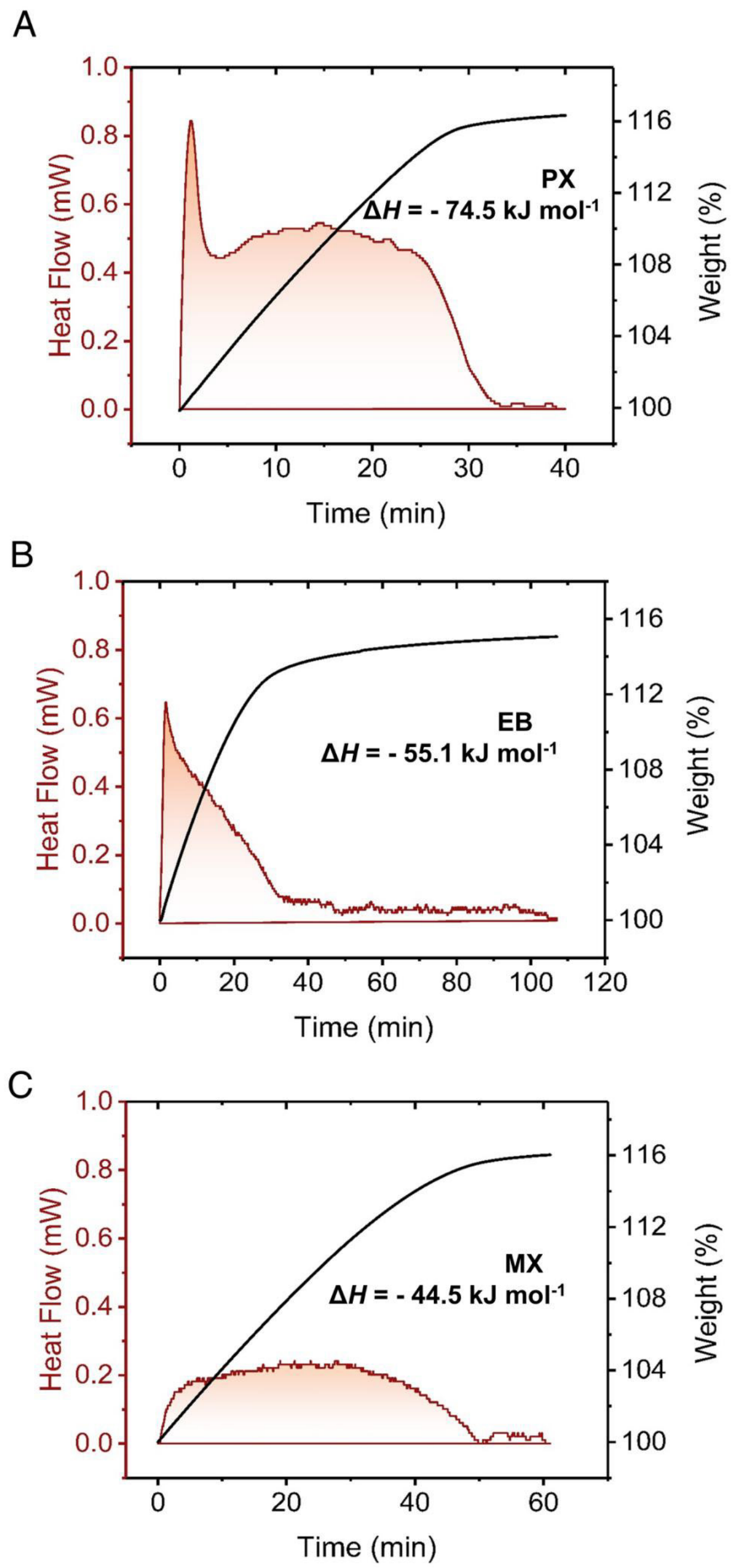

Fig. S10. Heat flow changes during the adsorption of (A) PX, (B) EB, and (C) MX in $Ni(BIC)_2$, as simultaneously measured by DSC and TG. Note that the weight change for OX was negligible due to the exclusion adsorption behavior of OX in $Ni(BIC)_2$, hence the adsorption heat for OX was not calculated.

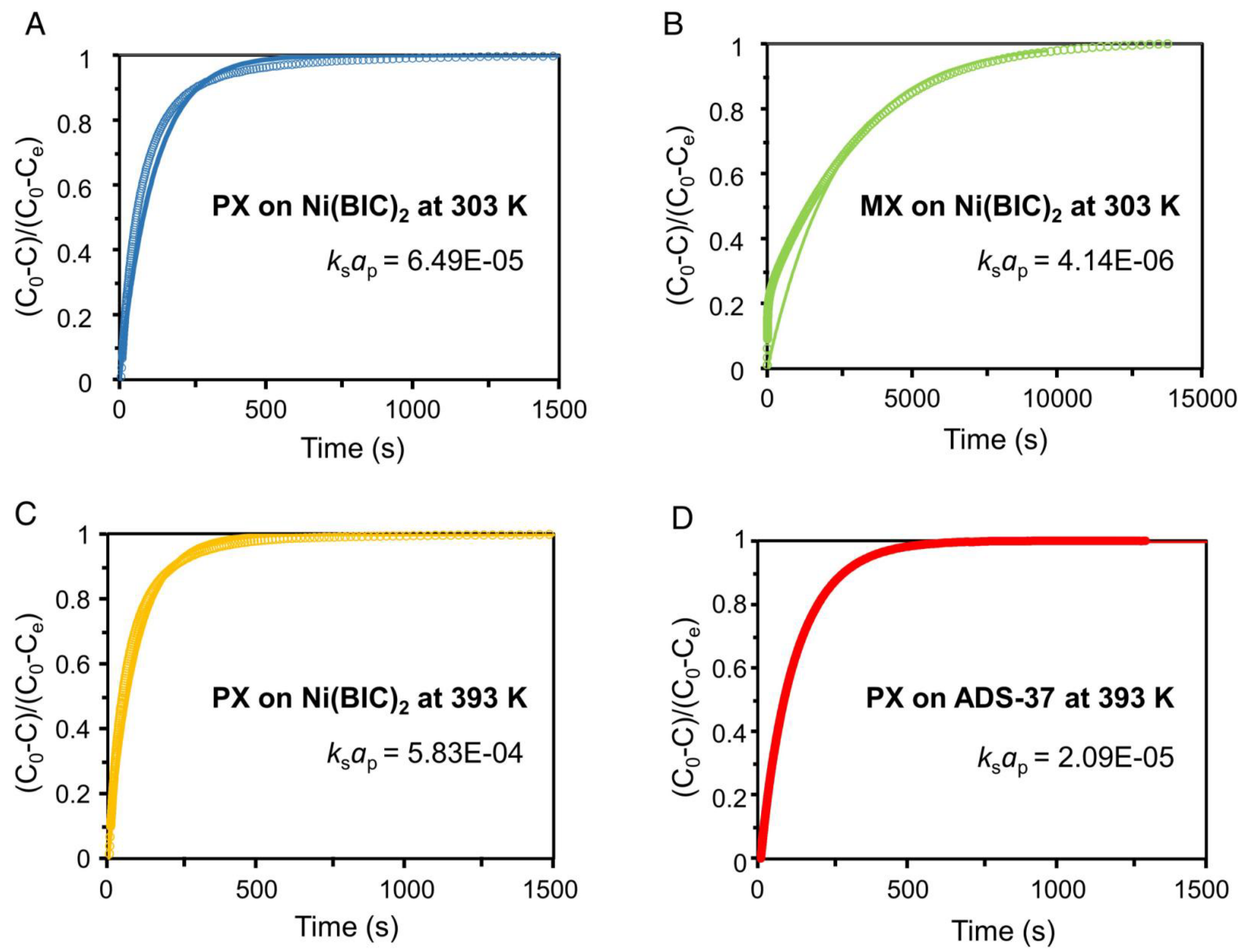

Fig. S11. Calculated mass-transfer coefficient ($k_s a_p$, s^{-1}) for (A) PX at 303 K, (B) MX at 303 K, and (C) PX at 393 K on $Ni(BIC)_2$, as well as (D) PX on ADS-37 (the commercial zeolite used in UOP Parex process) at 393 K, fitted with the linear driving force (LDF) model. C, C_0, and C_e refer to the transient concentration, initial concentration, and concentration at equilibrium, respectively.

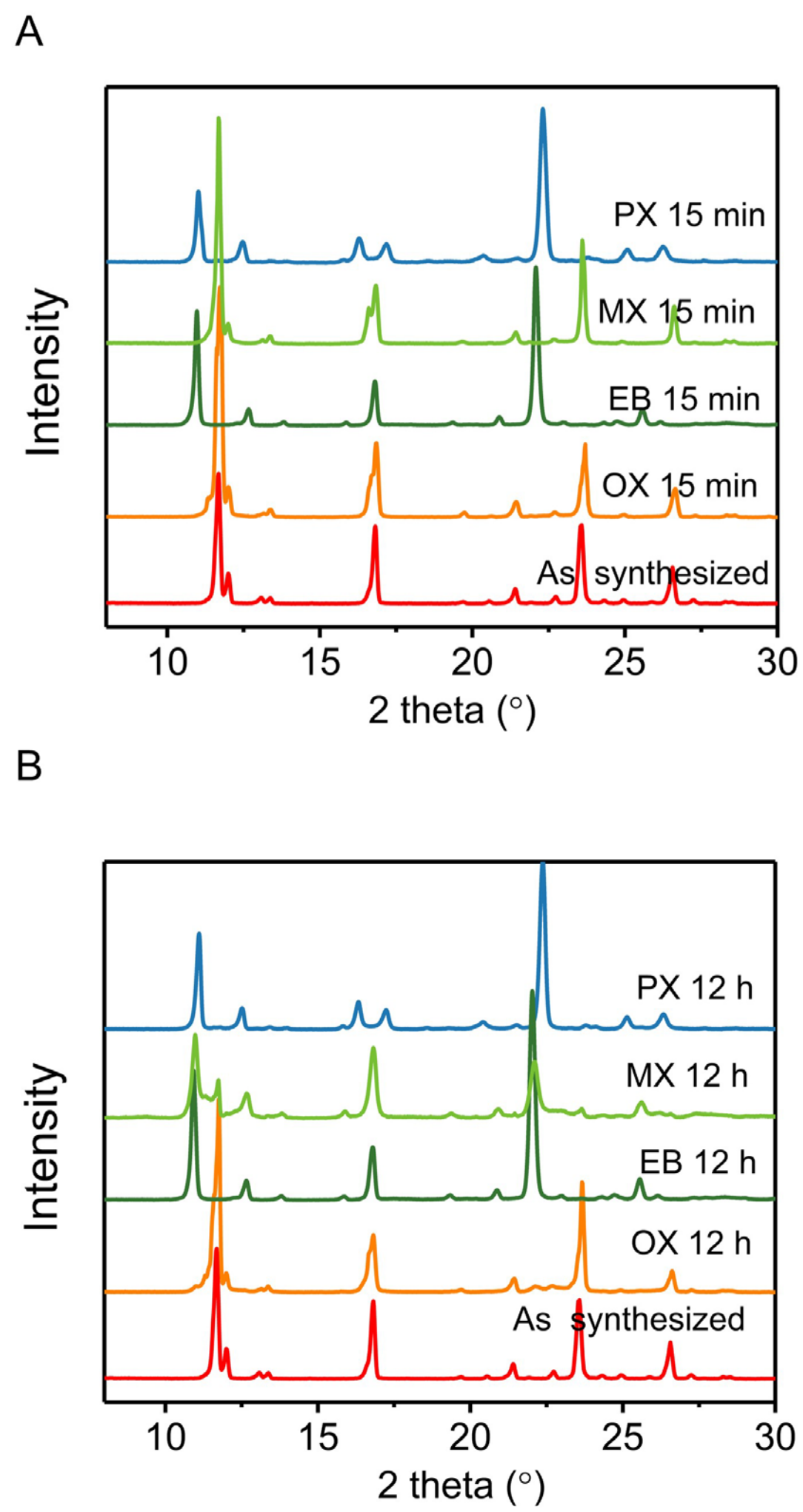

Fig. S12. Comparison of PXRD patterns for as-synthesized $Ni(BIC)_2$ and $Ni(BIC)_2$ loaded with PX, MX, OX, and EB at 303 K after a soaking time of (A) 15 min and (B) 12 h. The slight peak shifts and intensity variations observed among different xylene-loaded samples are mainly attributed to differences in guest packing and partial occupancies within the channels, rather than the formation of guest-specific framework phases.

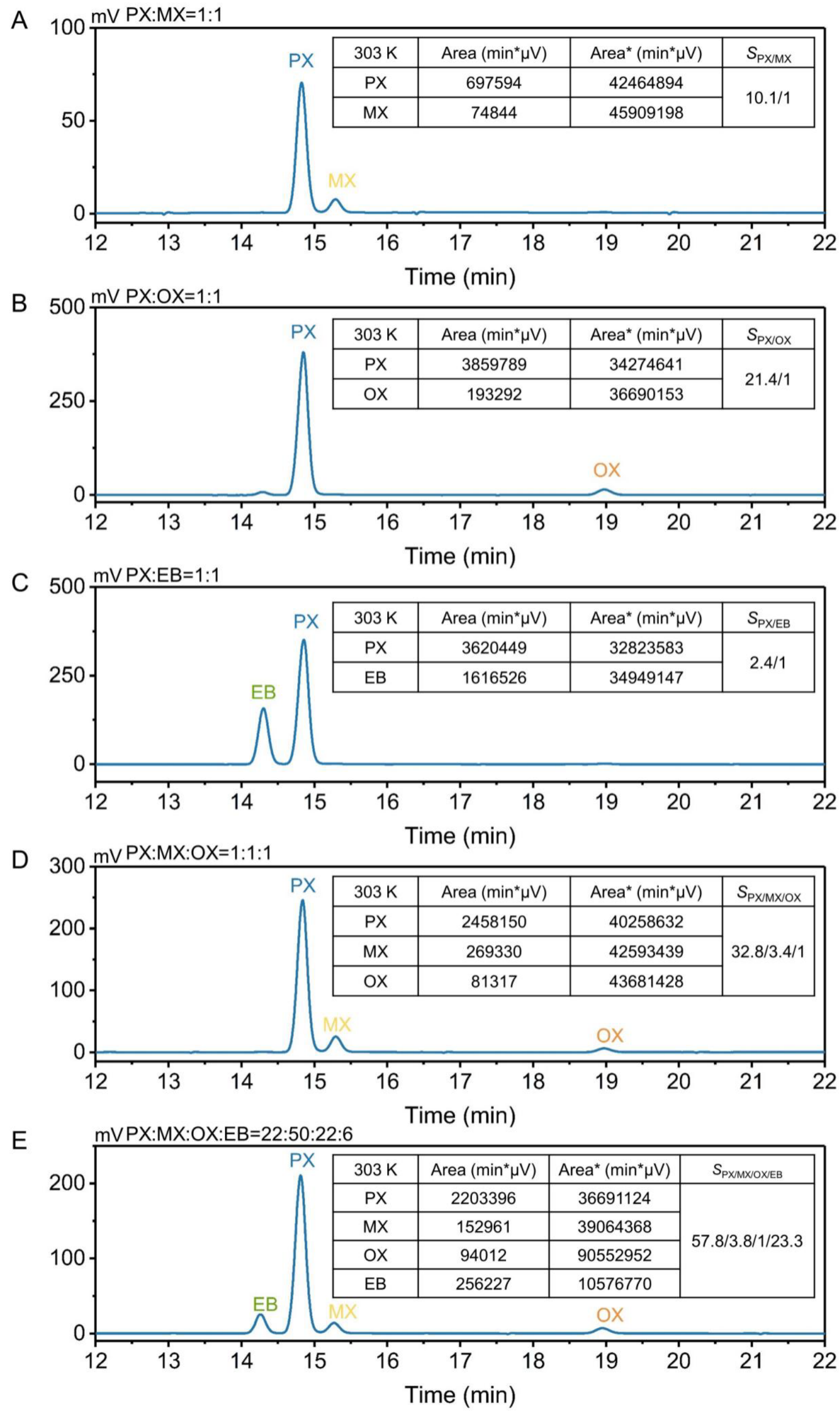

Fig. S13. Gas chromatograms for the solutions of xylene isomers, obtained by using CH_2Cl_2 to extract from $Ni(BIC)_2$ decomposed by concentrated HCl solution. The $Ni(BIC)_2$ was previously exposed to equimolar liquid mixtures of (A) PX/MX, (B) PX/OX, (C) PX/EB, (D) PX/MX/OX, and (E) a quaternary mixture of PX/MX/OX/EB with a molar ratio of 22:50:22:6 at 303 K. Note that Area* refers to the peak area of individual xylene components in the equilibrium solution.

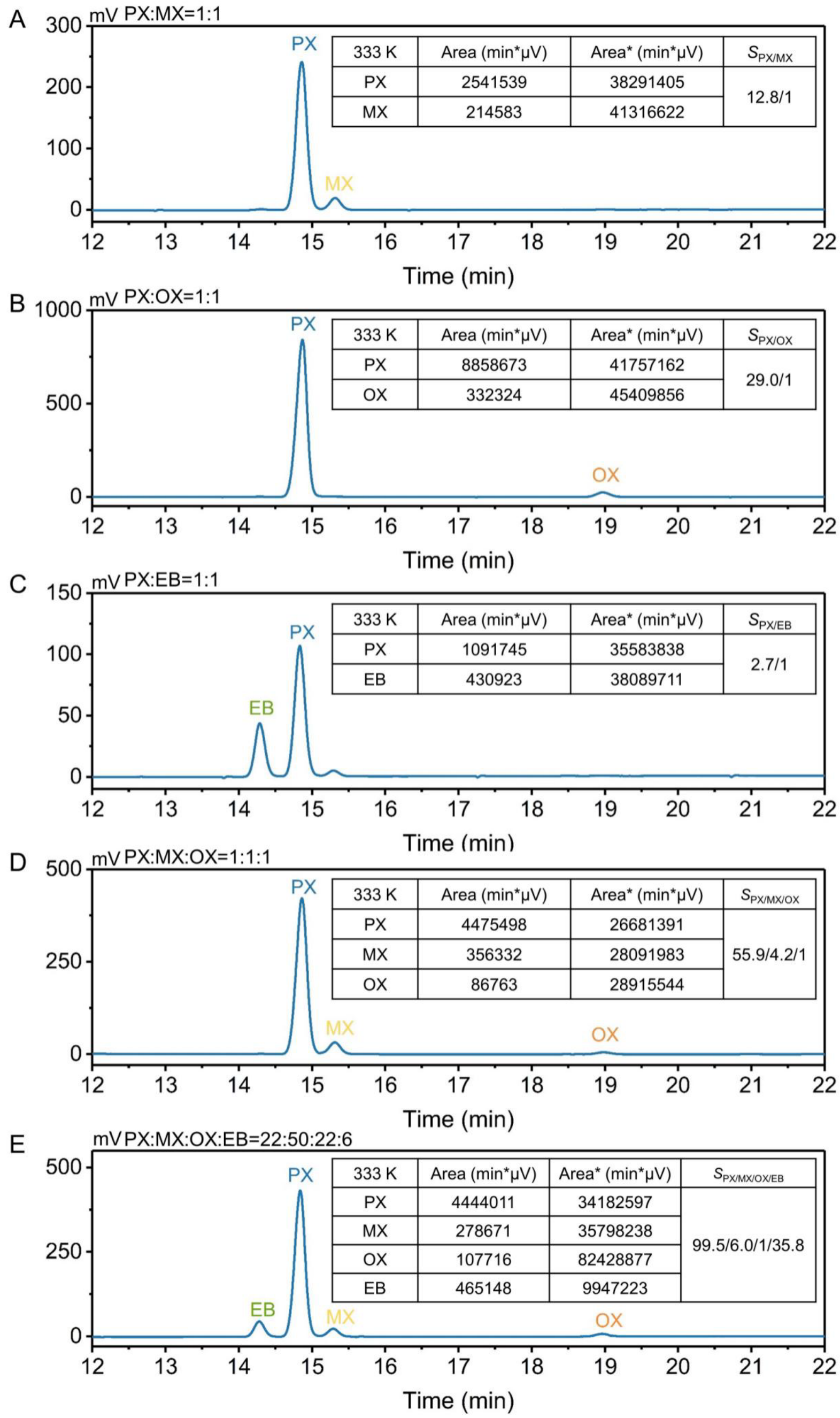

Fig. S14. Gas chromatograms for the solutions of xylene isomers, obtained by using CH_2Cl_2 to extract from $Ni(BIC)_2$ decomposed by concentrated HCl solution. The $Ni(BIC)_2$ was previously exposed to equimolar liquid mixtures of (A) PX/MX, (B) PX/OX, (C) PX/EB, (D) PX/MX/OX, and (E) a quaternary mixture of PX/MX/OX/EB with a molar ratio of 22:50:22:6 at 333 K. Note that Area* refers to the peak area of individual xylene components in the equilibrium solution.

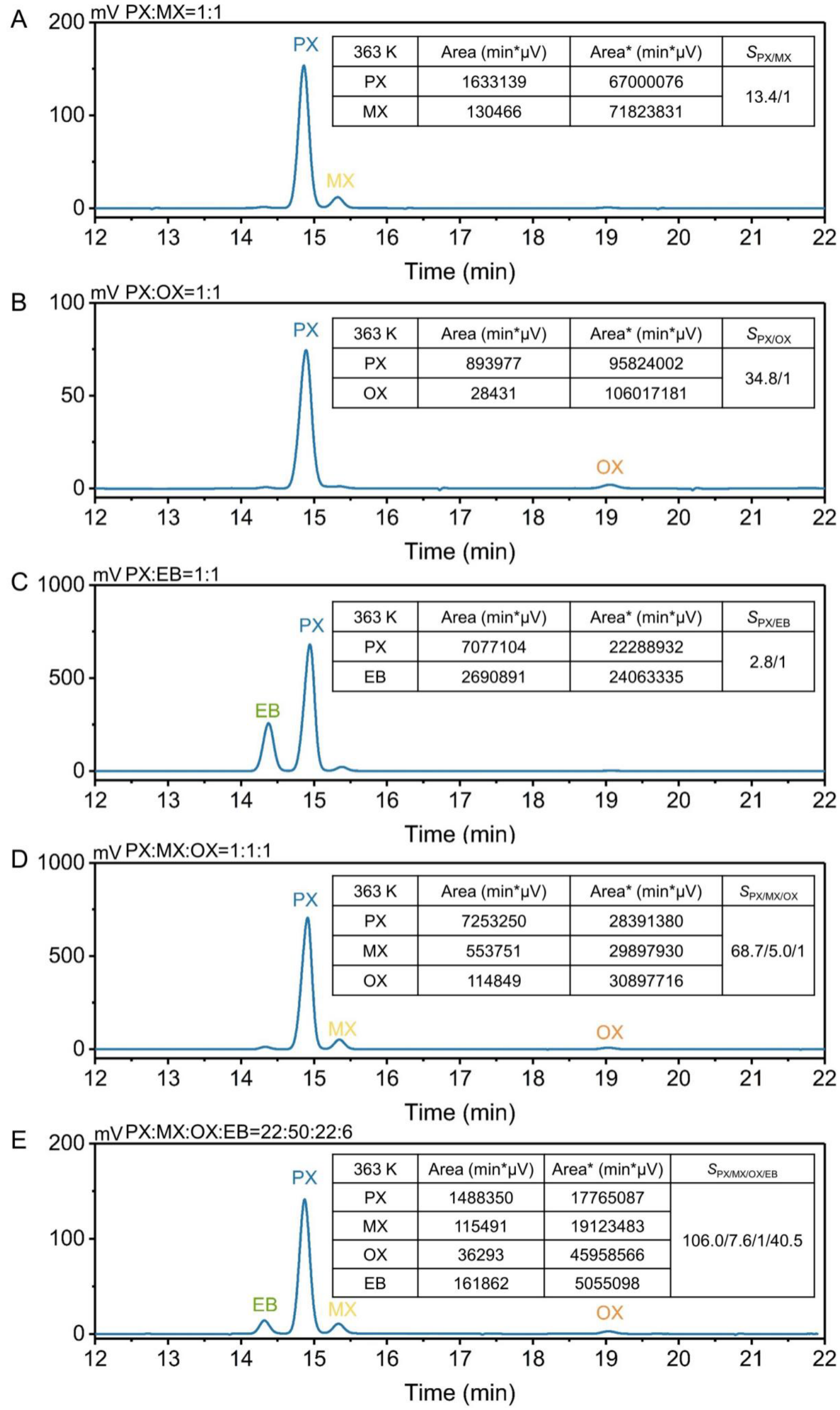

363 K	Area (min*μV)	Area* (min*μV)	$S_{PX/MX}$
PX	1633139	67000076	13.4/1
MX	130466	71823831	

363 K	Area (min*μV)	Area* (min*μV)	$S_{PX/OX}$
PX	893977	95824002	34.8/1
OX	28431	106017181	

363 K	Area (min*μV)	Area* (min*μV)	$S_{PX/EB}$
PX	7077104	22288932	2.8/1
EB	2690891	24063335	

363 K	Area (min*μV)	Area* (min*μV)	$S_{PX/MX/OX}$
PX	7253250	28391380	68.7/5.0/1
MX	553751	29897930	
OX	114849	30897716	

363 K	Area (min*μV)	Area* (min*μV)	$S_{PX/MX/OX/EB}$
PX	1488350	17765087	106.0/7.6/1/40.5
MX	115491	19123483	
OX	36293	45958566	
EB	161862	5055098	

Fig. S15. Gas chromatograms for the solutions of xylene isomers, obtained by using CH_2Cl_2 to extract from $Ni(BIC)_2$ decomposed by concentrated HCl solution. The $Ni(BIC)_2$ was previously exposed to equimolar liquid mixtures of (A) PX/MX, (B) PX/OX, (C) PX/EB, (D) PX/MX/OX, and (E) a quaternary mixture of PX/MX/OX/EB with a molar ratio of 22:50:22:6 at 363 K. Note that Area* refers to the peak area of individual xylene components in the equilibrium solution.

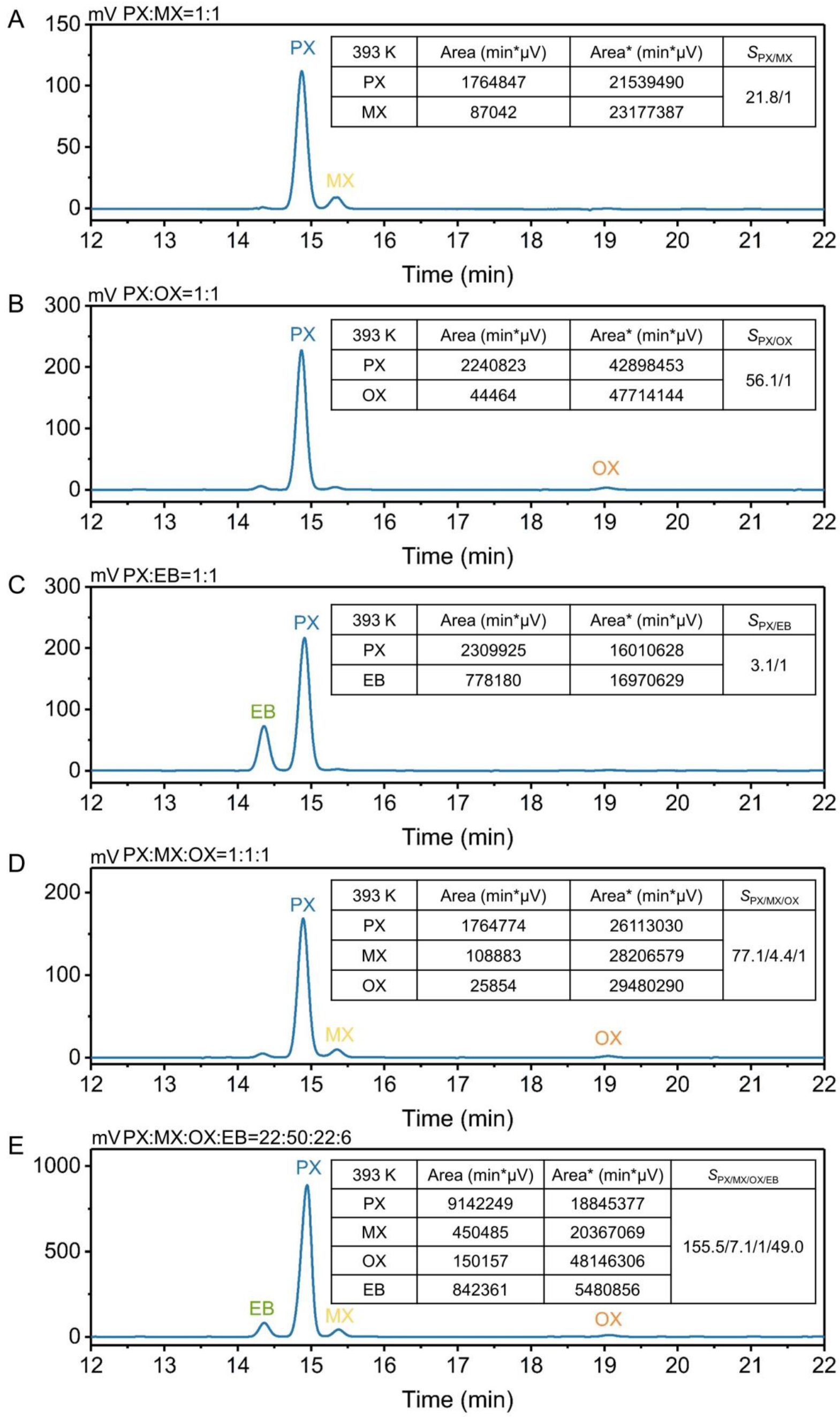

Fig. S16. Gas chromatograms for the solutions of xylene isomers, obtained by using CH_2Cl_2 to extract from Ni(BIC)$_2$ decomposed by concentrated HCl solution. The Ni(BIC)$_2$ was previously exposed to equimolar liquid mixtures of (A) PX/MX, (B) PX/OX, (C) PX/EB, (D) PX/MX/OX, and (E) a quaternary mixture of PX/MX/OX/EB with a molar ratio of 22:50:22:6 at 393 K. Note that Area* refers to the peak area of individual xylene components in the equilibrium solution.

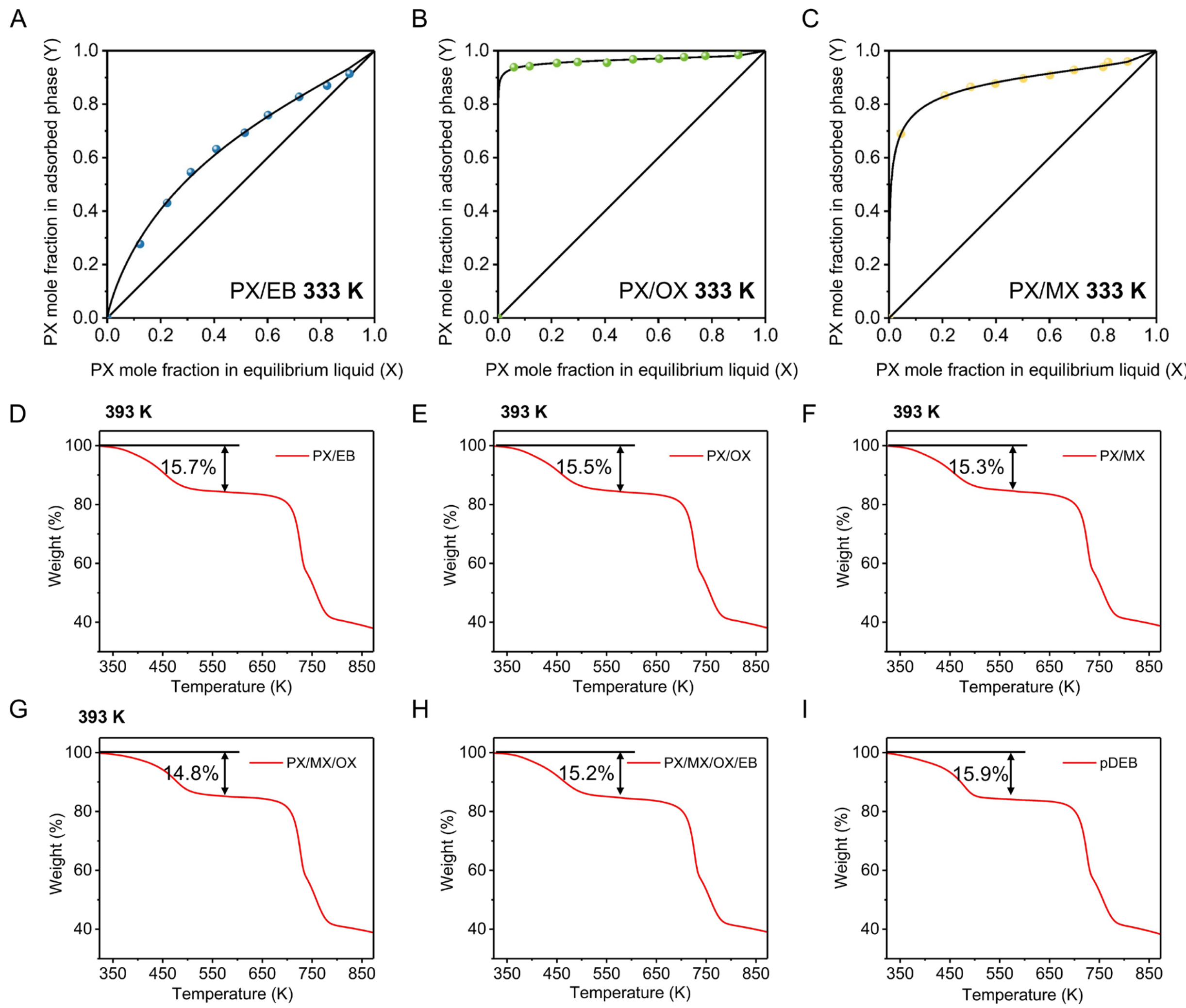

Fig. S17. Adsorption equilibrium at 333 K and the overall uptake amount at different constitutes at 393 K. (A-C) Adsorption equilibrium for binary solutions of (A) PX/EB, (B) PX/OX, and (C) PX/MX on $Ni(BIC)_2$ at 333 K. (D-I) Overall uptake amount of equimolar binary mixtures of (D) PX/EB, (E) PX/OX, and (F) PX/MX, as well as equimolar ternary liquid mixtures of (G) PX/MX/OX, and quaternary mixture of (H) PX/MX/OX/EB with molar ratio of 22:50:22:6 in batch liquid-phase selectivity experiments, and pure pDEB (I) at 393 K. All measurements were performed by TG analysis.

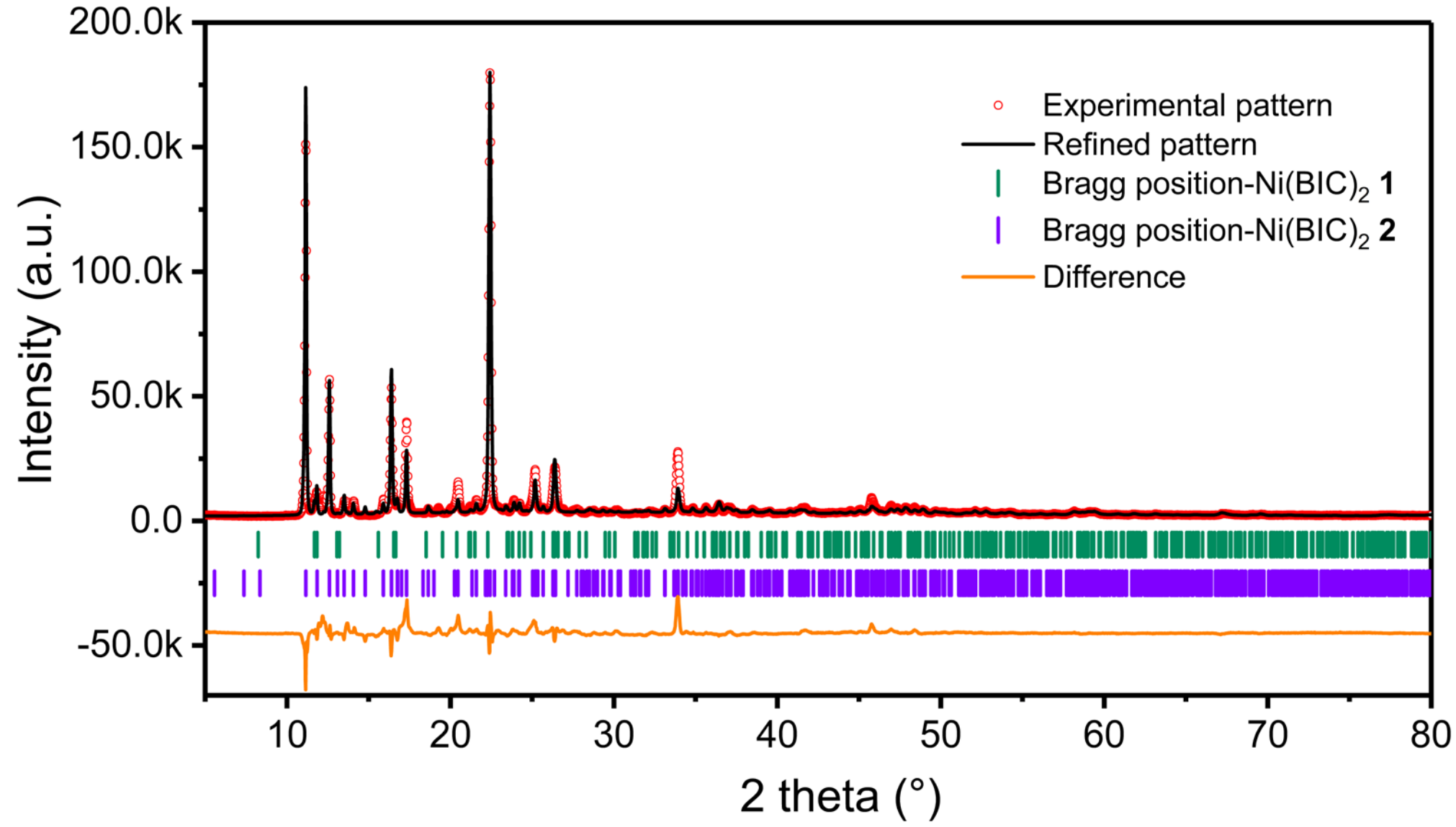

Fig. S18. Rietveld refinement of $Ni(BIC)_2$ after exposure to PX (R_p: 11.720%, R_{wp}: 17.342%).

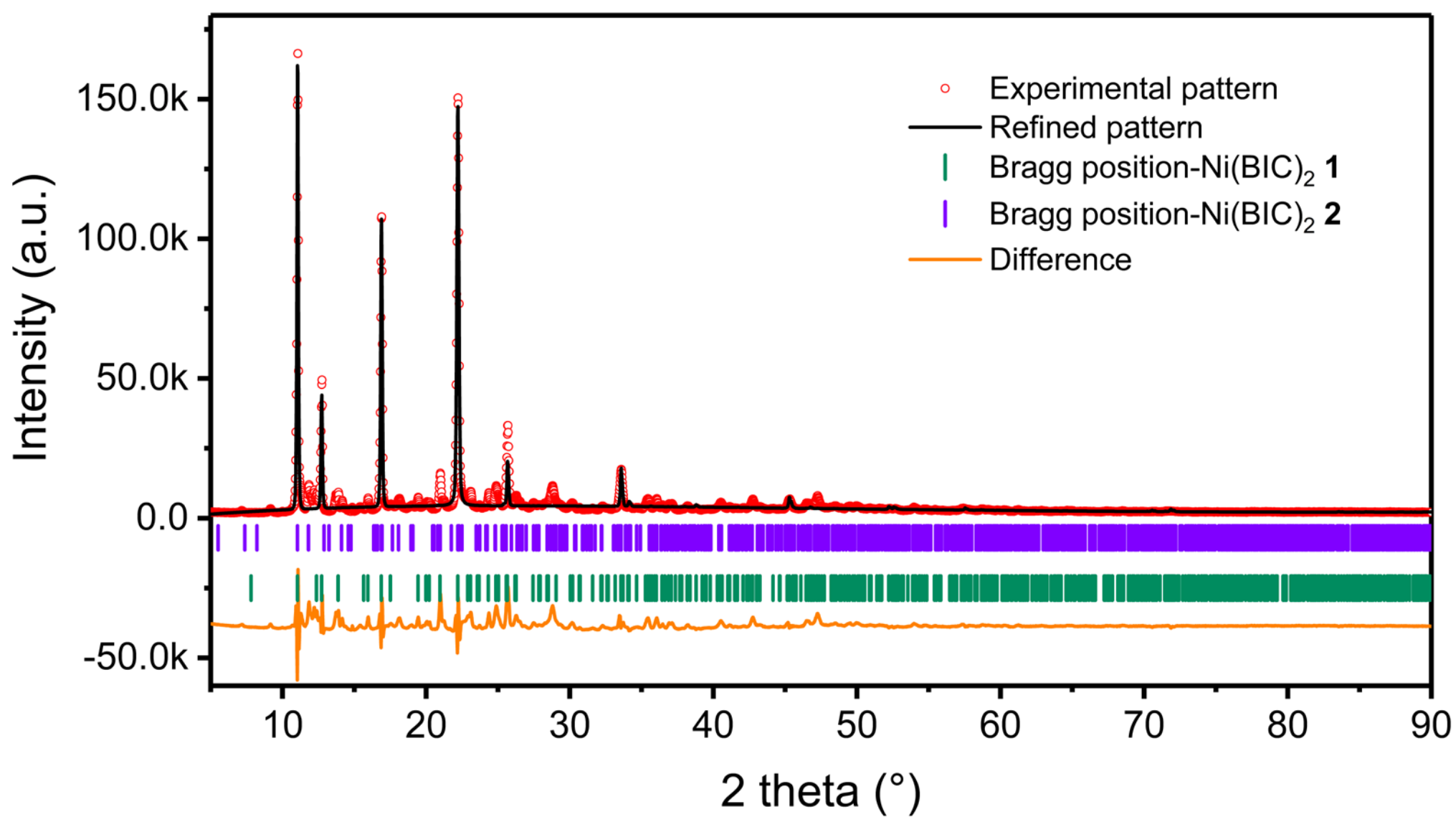

Fig. S19. Rietveld refinement of $Ni(BIC)_2$ after exposure to EB (R_p: 15.250%, R_{wp}: 21.665%).

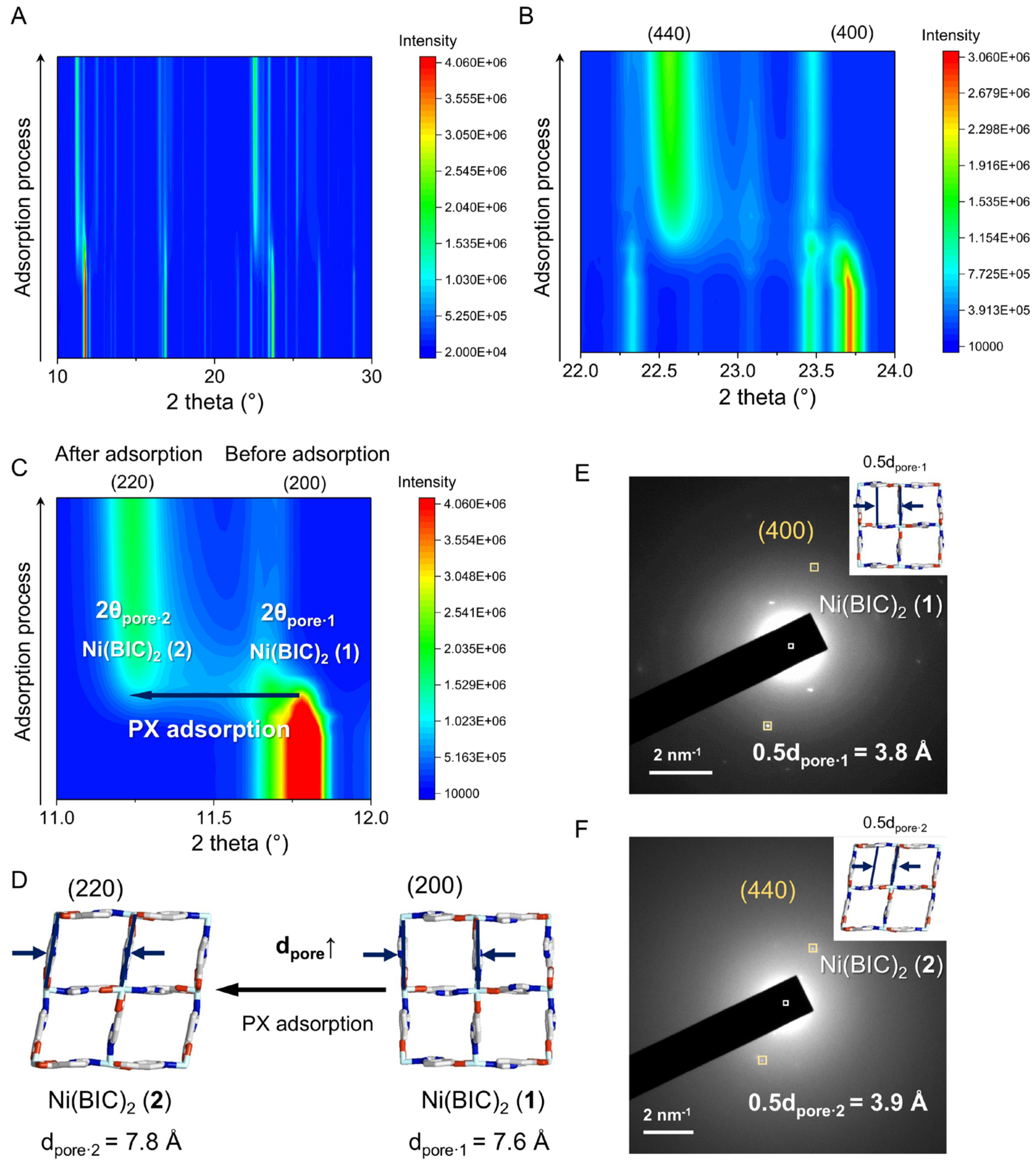

Fig. S20. Structural transformation of Ni(BIC)$_2$ in PX adsorption. In-situ PXRD patterns of Ni(BIC)$_2$ under PX vapor conditions at 303 K in 2θ range of (A) 10°-30°, (B) 22°-24°, and (C) 11°-12°. (D) Single-crystal-to-single-crystal transition of Ni(BIC)$_2$ during PX adsorption. (E-F) FFT pattern along the direction of pore-diameter (E) before and (F) after PX adsorption. HRTEM images in panels E and F are reproduced from Fig. 3C and Fig. 3D, respectively, for comparison with the PX adsorption results.

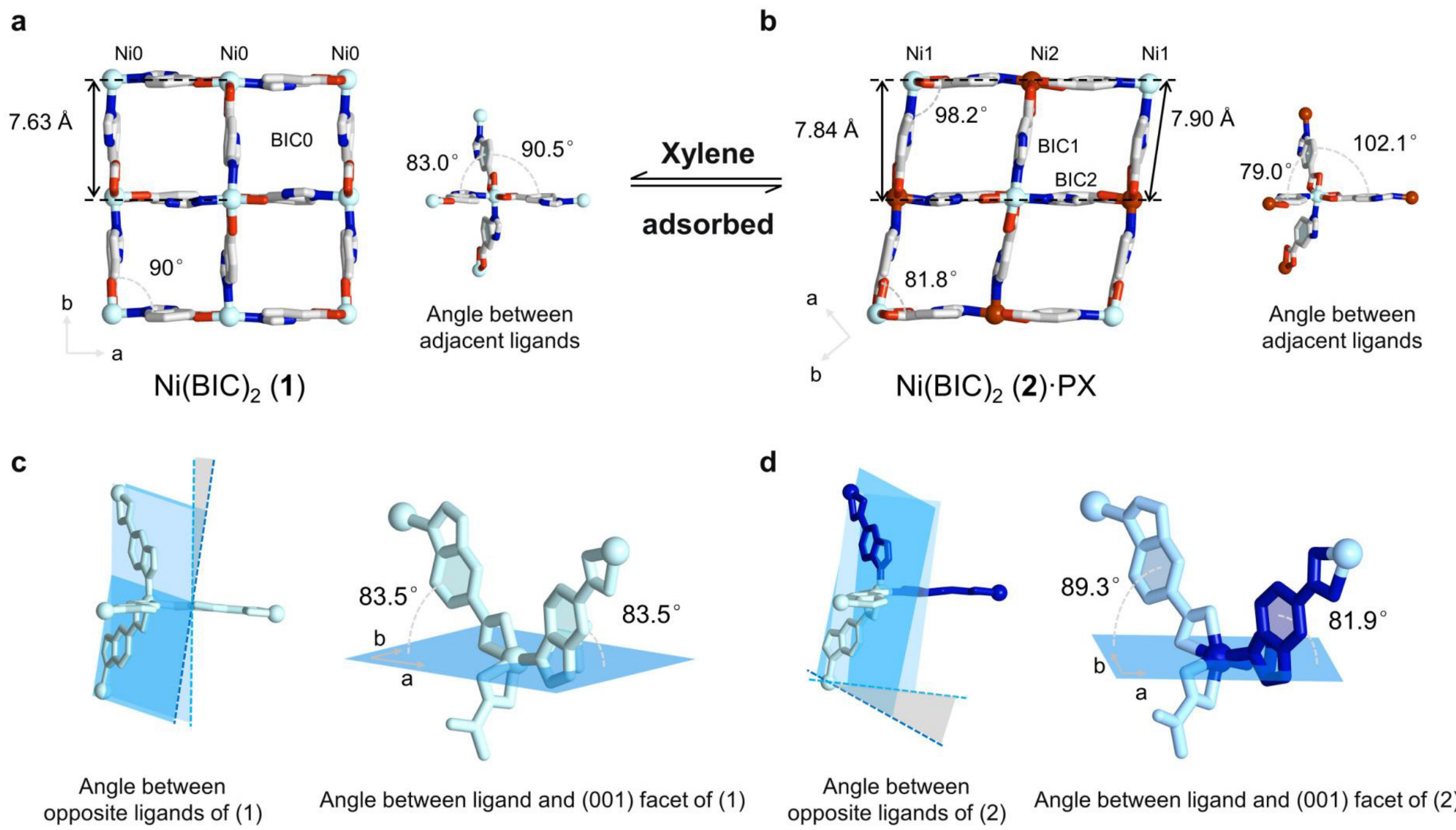

Fig. S21. A more specific scheme showing the structural transformation of $Ni(BIC)_2$ in PX adsorption. (A-B) Structural transformation from $Ni(BIC)_2$ (**1**) to $Ni(BIC)_2$ (**2**). The symmetry of the activated adsorbent (termed as $Ni(BIC)_2$ (**1**)) undergoes a significant decrease upon the adsorption of PX (termed as $Ni(BIC)_2$ (**2**)), as the space group number shifts from 96 to 20. All equivalent Ni_0 center and BIC0 ligands in $Ni(BIC)_2$ (**1**) transforms into two non-equivalent Ni1/Ni2 centers and BIC1/BIC2 ligands in $Ni(BIC)_2$ (**2**). The angle change of (C) $Ni(BIC)_2$ (**1**) and (D) $Ni(BIC)_2$ (**2**). The channel angles shift from 90°/90° to 81.8°/98.2°. Alterations occur in pore dimensions, with the edge length shifting from 7.63 Å to 7.90 Å and the height of the quadrilateral pores changing from 7.63 Å to 7.84 Å. Induced deformation by adsorbate causes a change in adjacent ligand dihedral angles from 83.0°/89.5° to 77.9°/79.0°, a change in dihedral angle facing between opposite ligands, and the angle change between ligand and (001) facet from 83.5°/83.5° to 89.3°/81.9°. The structure revealed a slight distortion of the adjacent and opposite ligands relative to the activated structure, reflected in the reduced symmetry mentioned before. These changes suggest a guest-adaptive lattice transformation from square to rhombic pores upon xylene adsorption, indicating that adaptation to the molecular projection shape channel allowed molecular projection shape discrimination with increasing temperature.

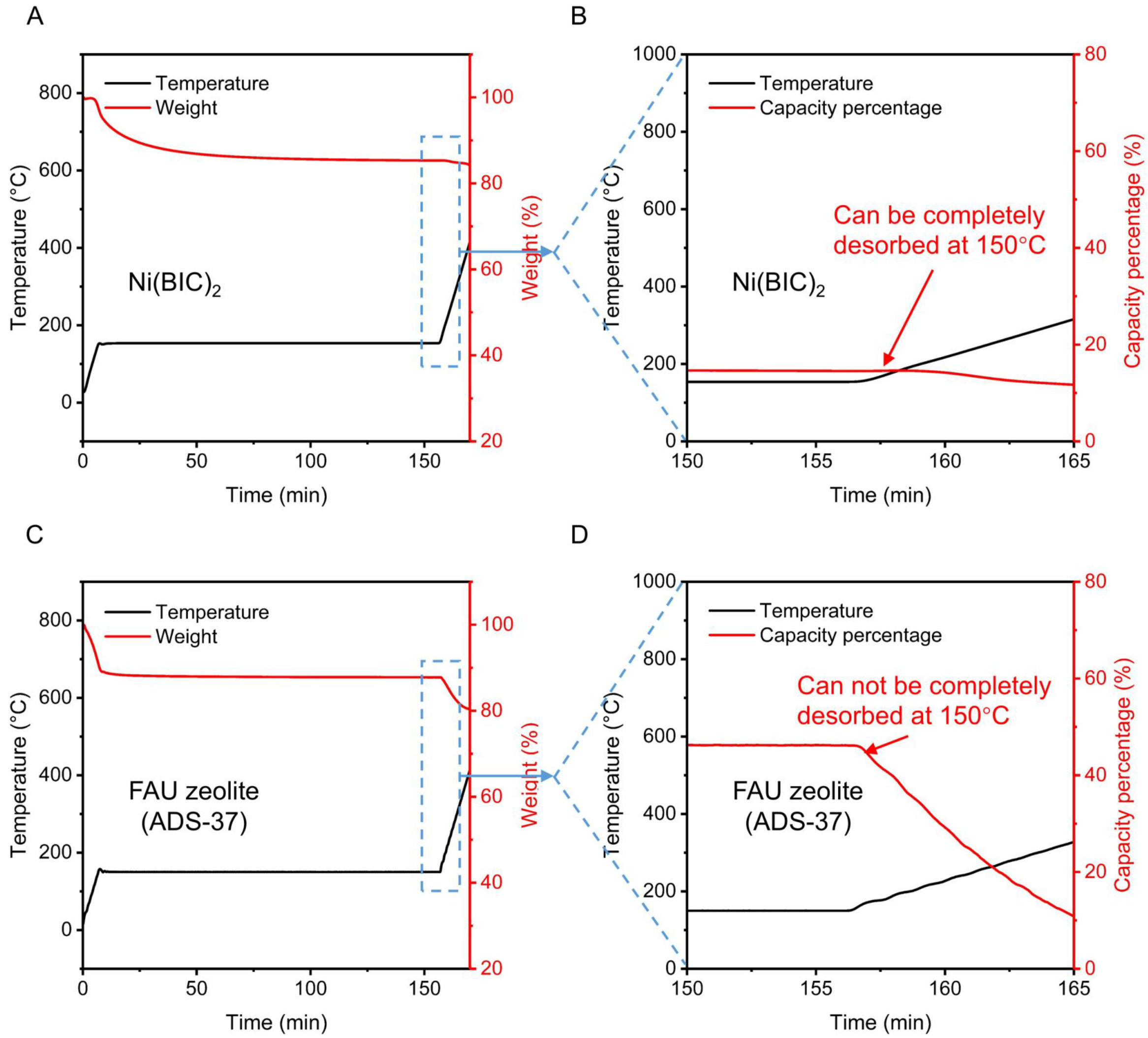

Fig. S22. Thermogravimetric (TG) profiles of the desorption process on (A) $Ni(BIC)_2$ and (C) FAU-type zeolite (ADS-37). (B) and (D) present the corresponding enlarged views of the regions marked in (A) and (C), respectively. In the enlarged views, the y-axis is converted to a percentage of adsorption capacity for clearer visualization. Samples (50 g each) were pre-degassed (MOF: vacuum at 150 °C; zeolite: vacuum at 300 °C), then soaked overnight in pure p-xylene liquid. After filtration and one day of drying, the samples were subjected to TGA. The heating program: ramp at 20 °C min^{-1} to 150 °C, hold for 150 min, and then continue heating at 20 °C min^{-1} to examine whether the samples were essentially activated at 150 °C. The results show that both samples reached mass equilibrium during the 150 °C isothermal step. Upon further heating from 150 °C, the zeolite sample exhibited a pronounced weight loss, whereas the MOF sample showed a gradual decrease. This experiment demonstrates that framework flexibility benefits the desorption process.

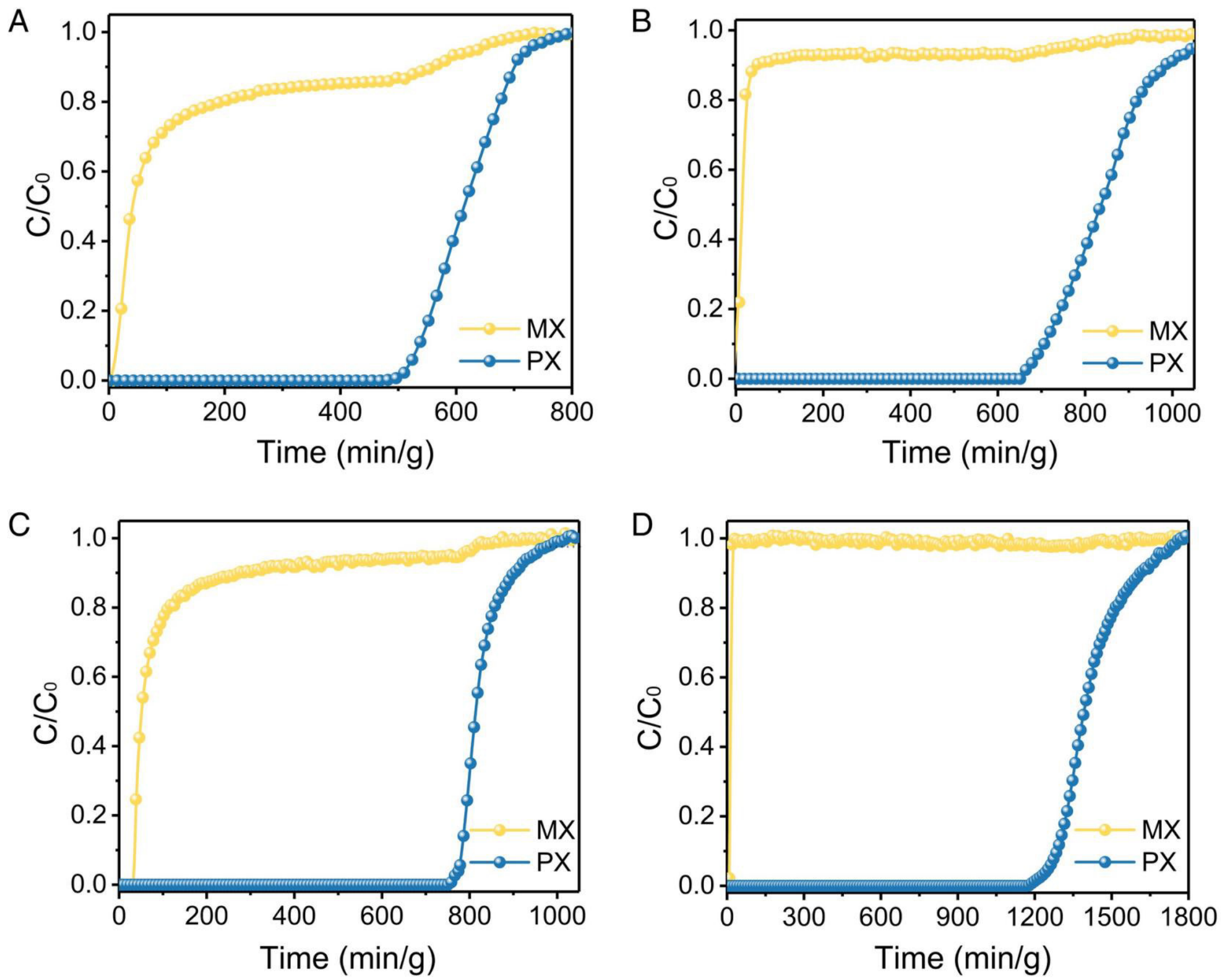

Fig. S23. Binary vapor-phase breakthrough curves for an equimolar mixture of MX (yellow) and PX (blue) on $Ni(BIC)_2$ at temperatures of (A) 303, (B) 333, (C) 363, and (D) 393 K.

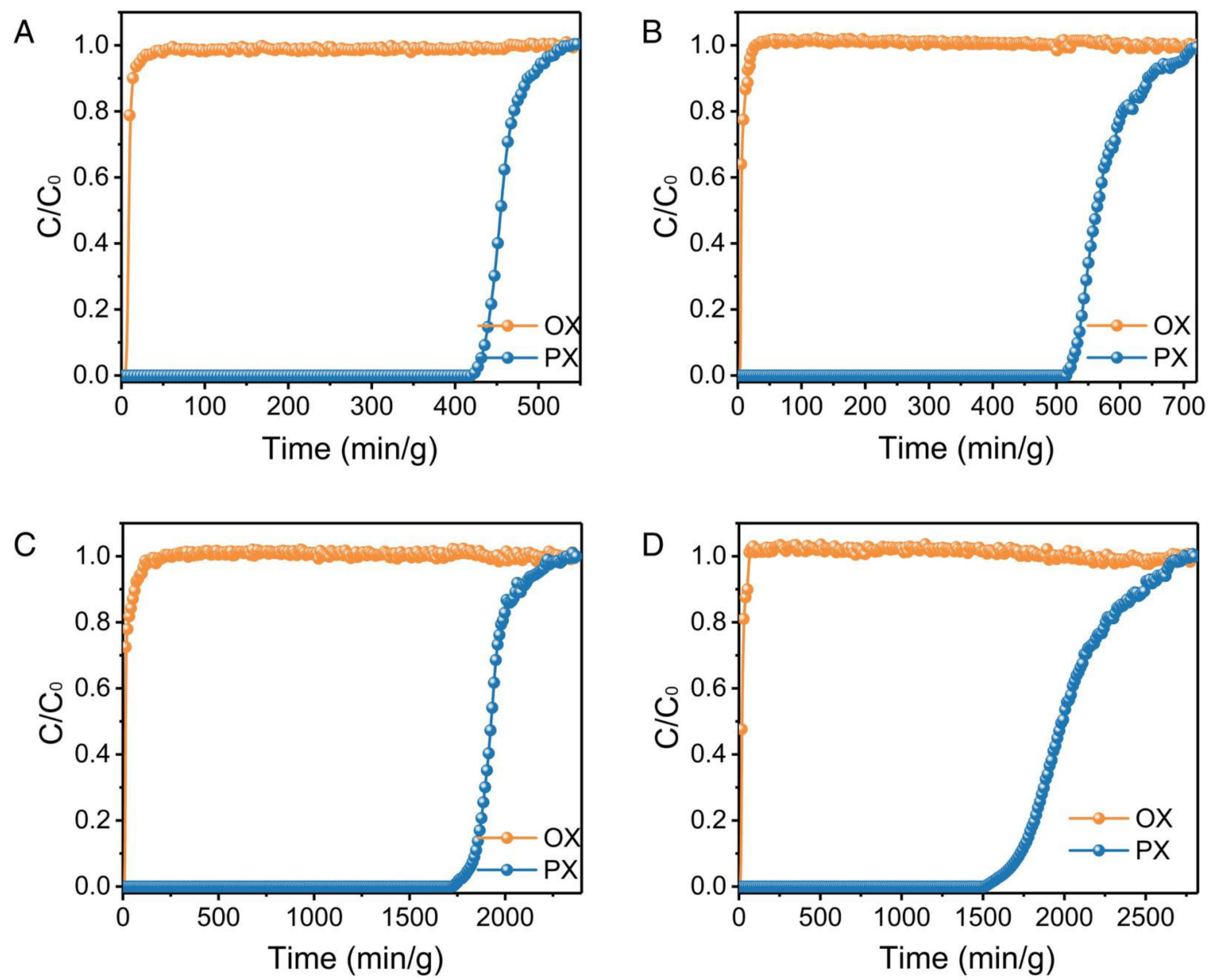

Fig. S24. Binary vapor-phase breakthrough curves for an equimolar mixture of OX (orange) and PX (blue) on $Ni(BIC)_2$ at temperatures of (A) 303, (B) 333, (C) 363, and (D) 393 K.

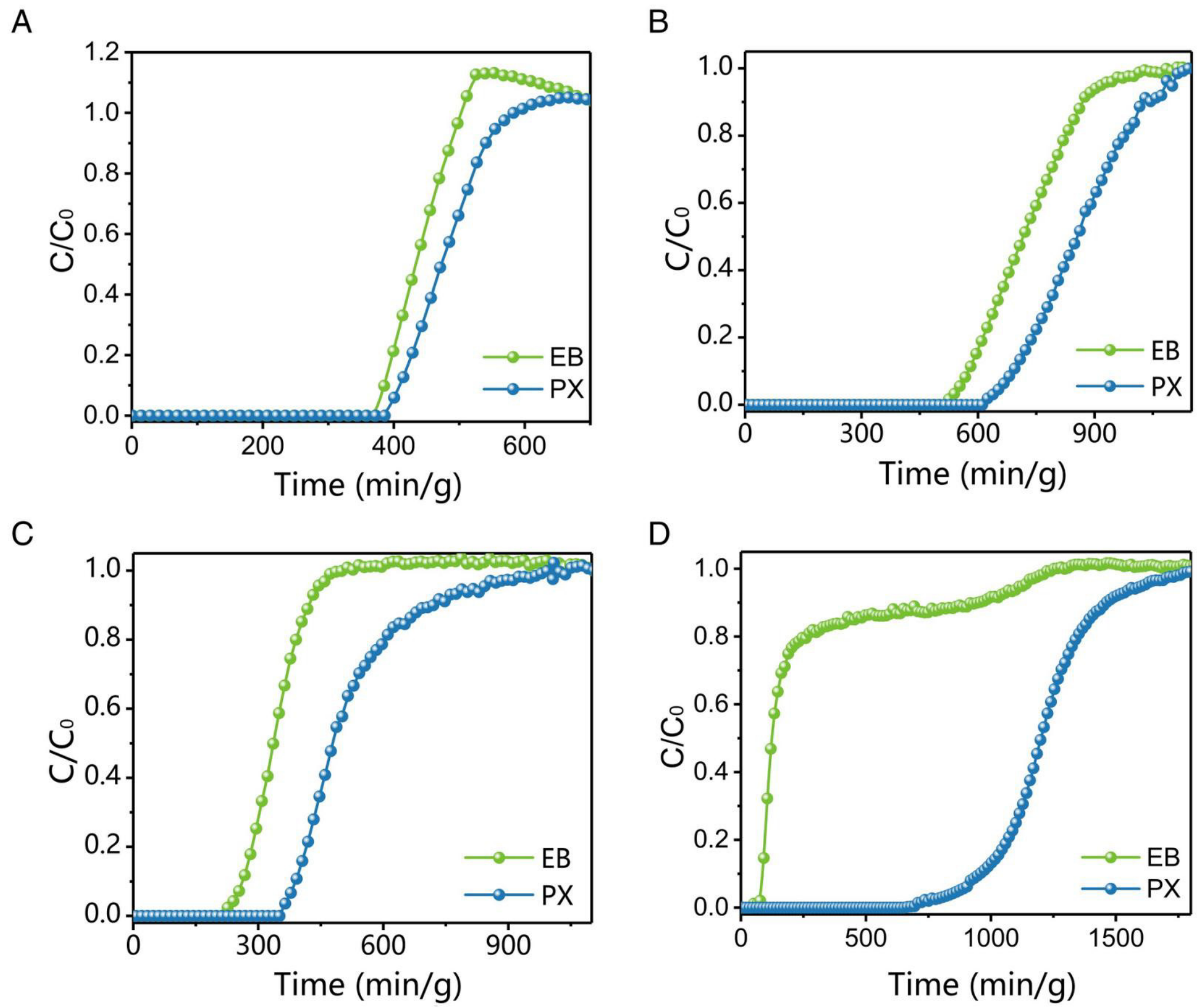

Fig. S25. Binary vapor-phase breakthrough curves for an equimolar mixture of EB (green) and PX (blue) on $Ni(BIC)_2$ at temperatures of (A) 303, (B) 333, (C) 363, and (D) 393 K.

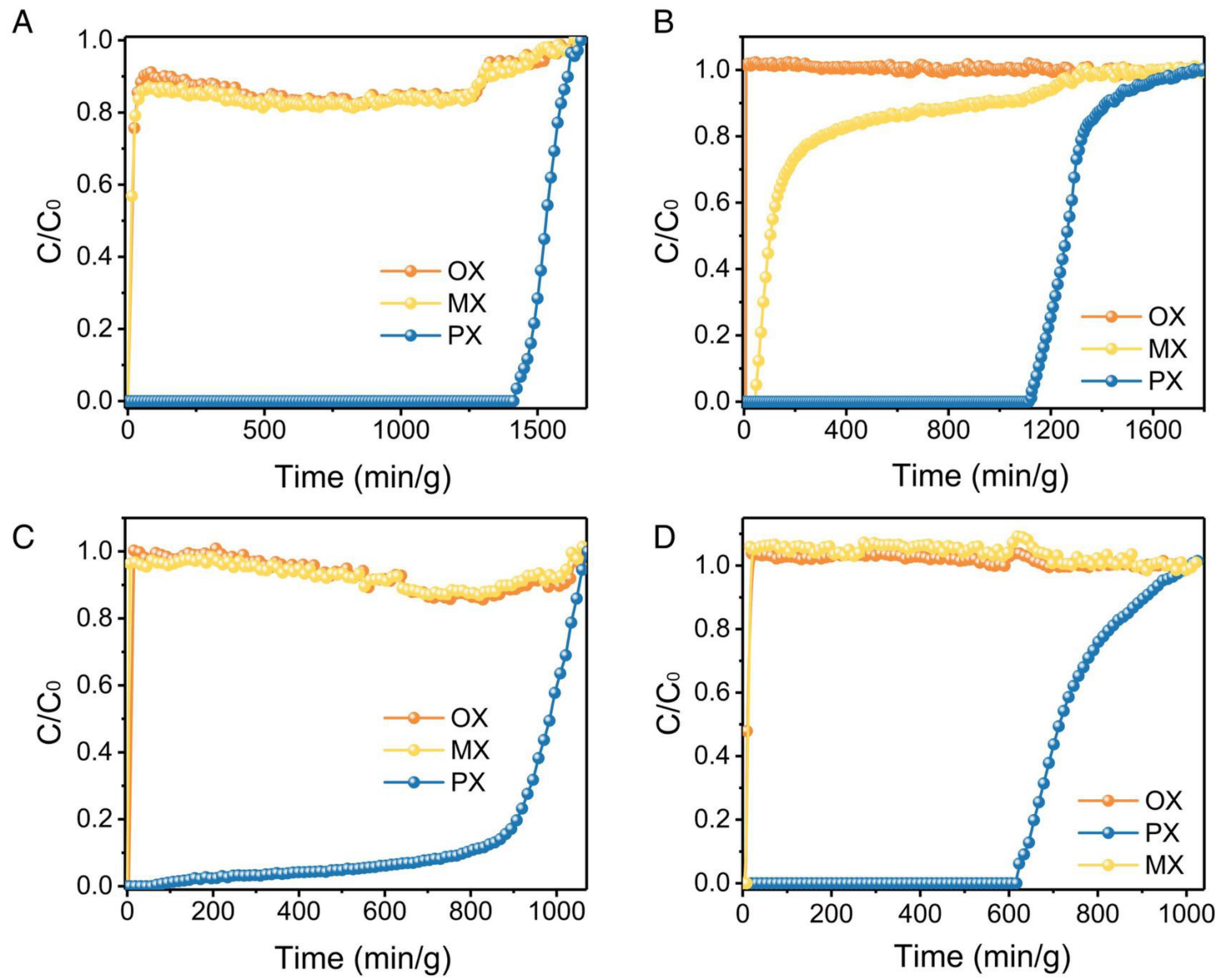

Fig. S26. Ternary vapor-phase breakthrough curves for an equimolar mixture of OX (orange), MX (yellow), and PX (blue) on $Ni(BIC)_2$ at temperatures of (A) 303, (B) 333, (C) 363, and (D) 393 K.

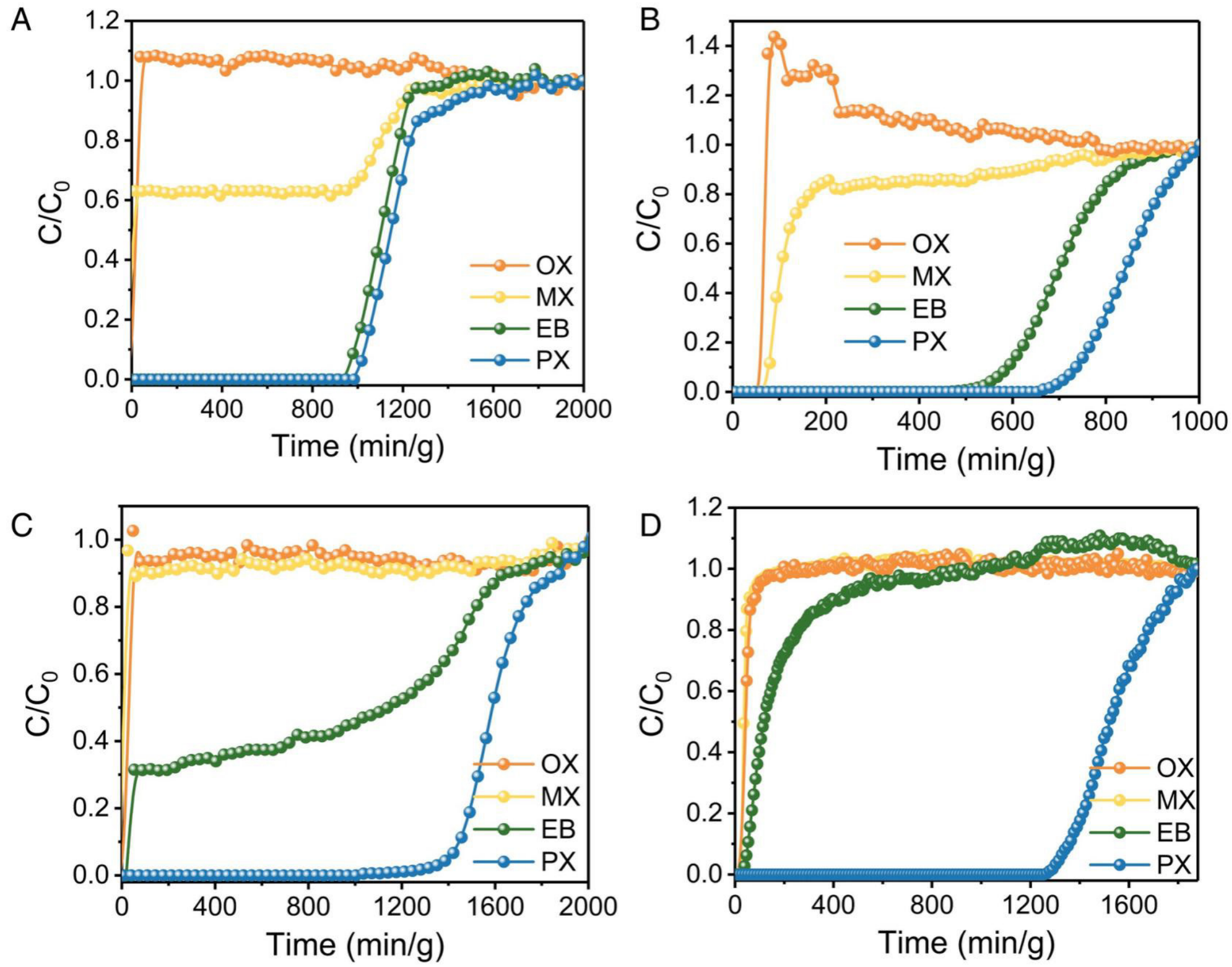

Fig. S27. Vapor-phase breakthrough curves for a quaternary mixture of OX (orange), MX (yellow), EB (green) and PX (blue) with a ratio of OX/MX/PX/EB = 22/50/22/6 on Ni(BIC)$_2$ at temperatures of (A) 303, (B) 333, (C) 363, and (D) 393 K.

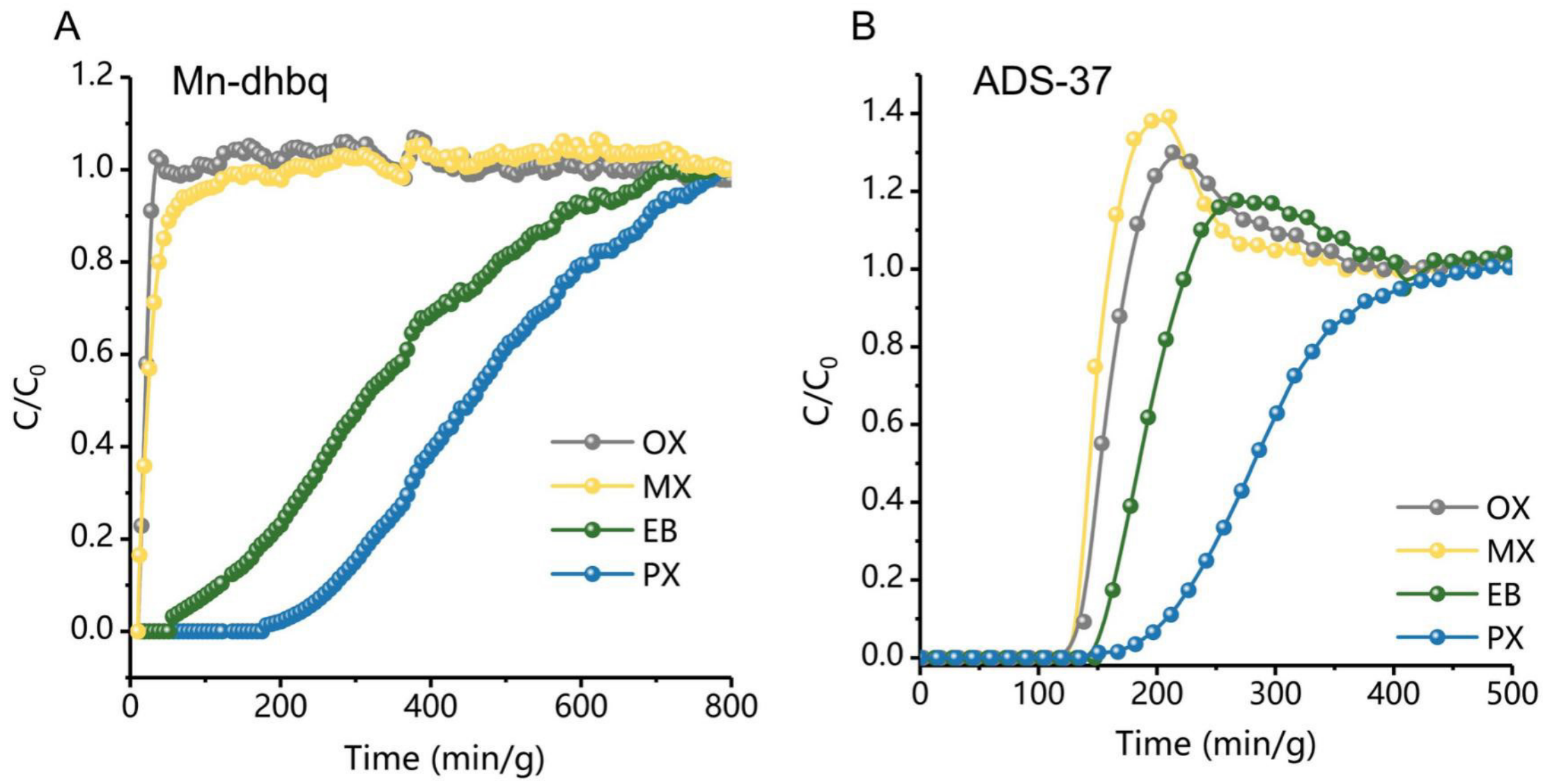

Fig. S28. Vapor-phase breakthrough curves for a quaternary mixture of OX (gray), MX (yellow), EB (green), and PX (blue) with a mass ratio of OX/MX/PX/EB = 22/50/22/6 on (A) Mn-dhbq and (B) ADS-37 at 393 K.

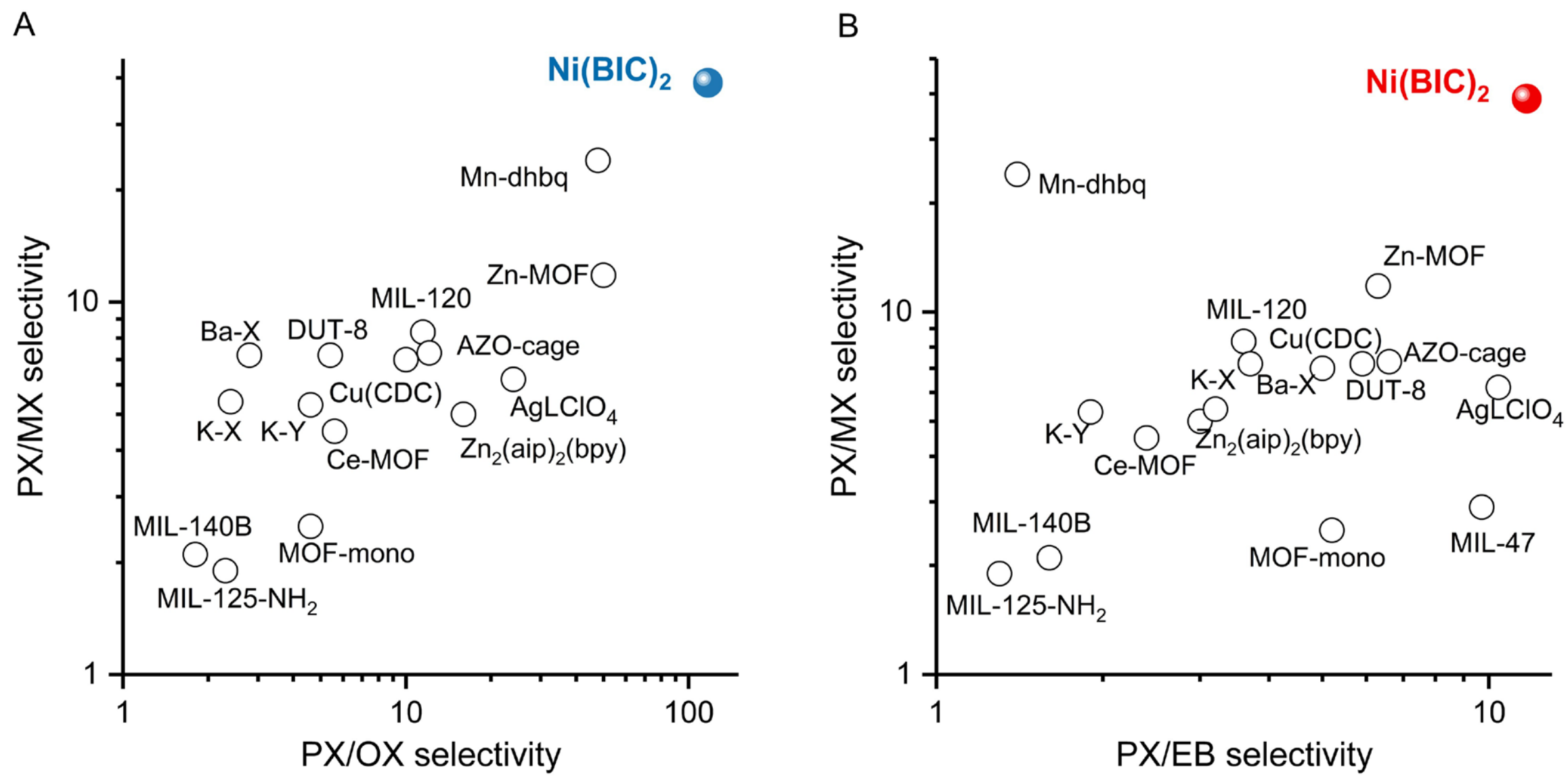

Fig. S29. Comparison of PX/MX, PX/OX, and PX/EB adsorption selectivity. (A) Comparison of adsorption selectivity for PX/MX and PX/OX from breakthrough experiments on Ni(BIC)$_2$ using a mixture of PX/MX/OX/EB at 393 K, along with other materials reported for separating quaternary C8 isomer mixtures. (B) Comparison of adsorption selectivity for PX/MX and PX/EB from breakthrough experiments on Ni(BIC)$_2$ for PX/MX/OX/EB mixtures at 393 K and other materials reported for separating quaternary C8 isomer mixtures. Noting that for each reported material, only the highest and/or best values of selectivity were selected, regardless of the phase (liquid or vapor) of the adsorbate during the experiments.

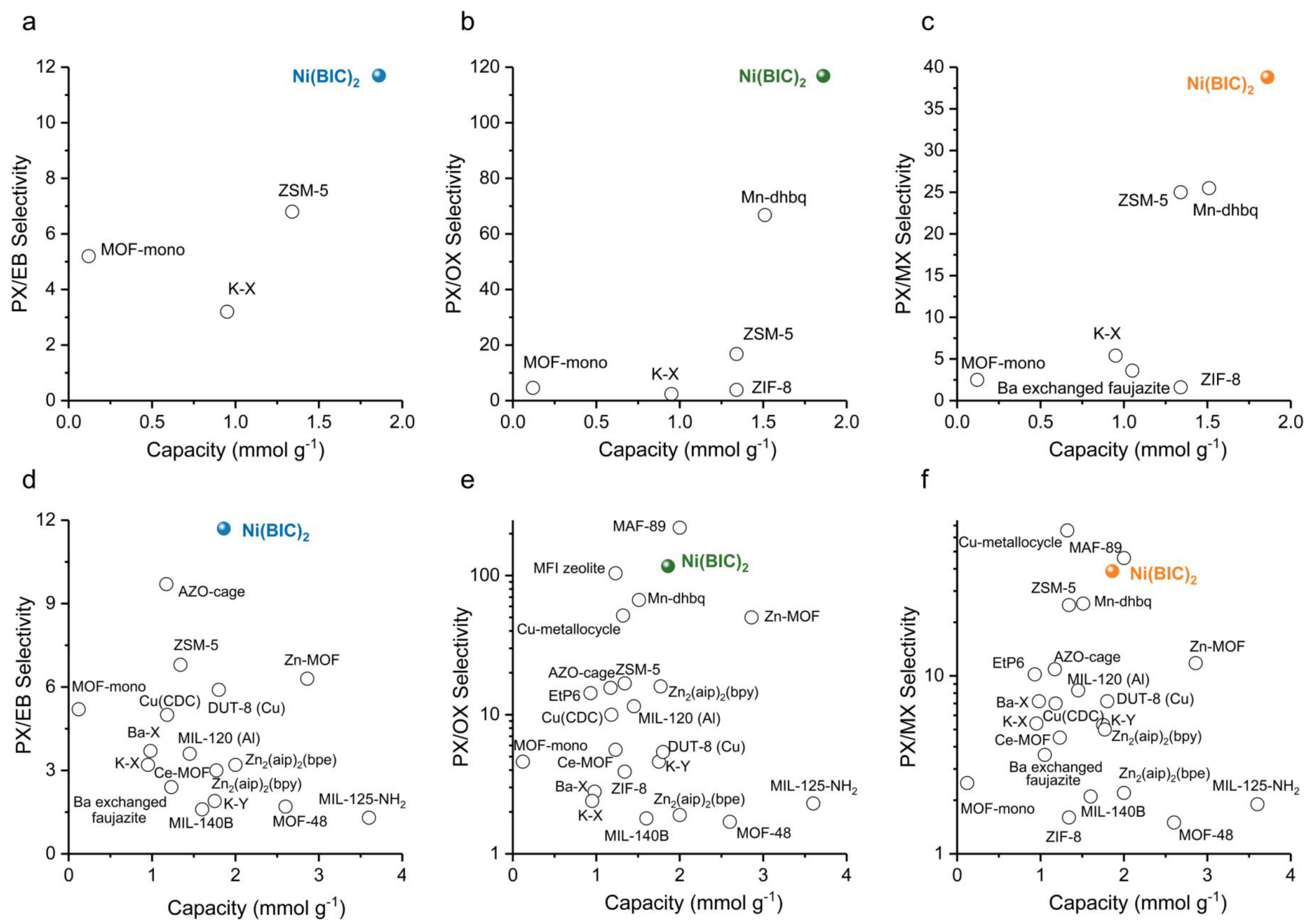

Fig. S30. Performance comparison on all PX-adsorbents. (A)-(C) Comparison of PX capacity with (A) PX/EB, (B) PX/OX, and (C) PX/MX adsorption selectivity at the optimal operating conditions at the same operating condition (at 393 K). (D)-(F) Comparison of PX capacity with (D) PX/EB, (E) PX/OX, and (F) PX/MX adsorption selectivity at the optimal operating conditions on each adsorbent. Noting that for each reported material, only the highest and/or best values of selectivity were selected, regardless of the phase (liquid or vapor) of the adsorbate during the experiments.

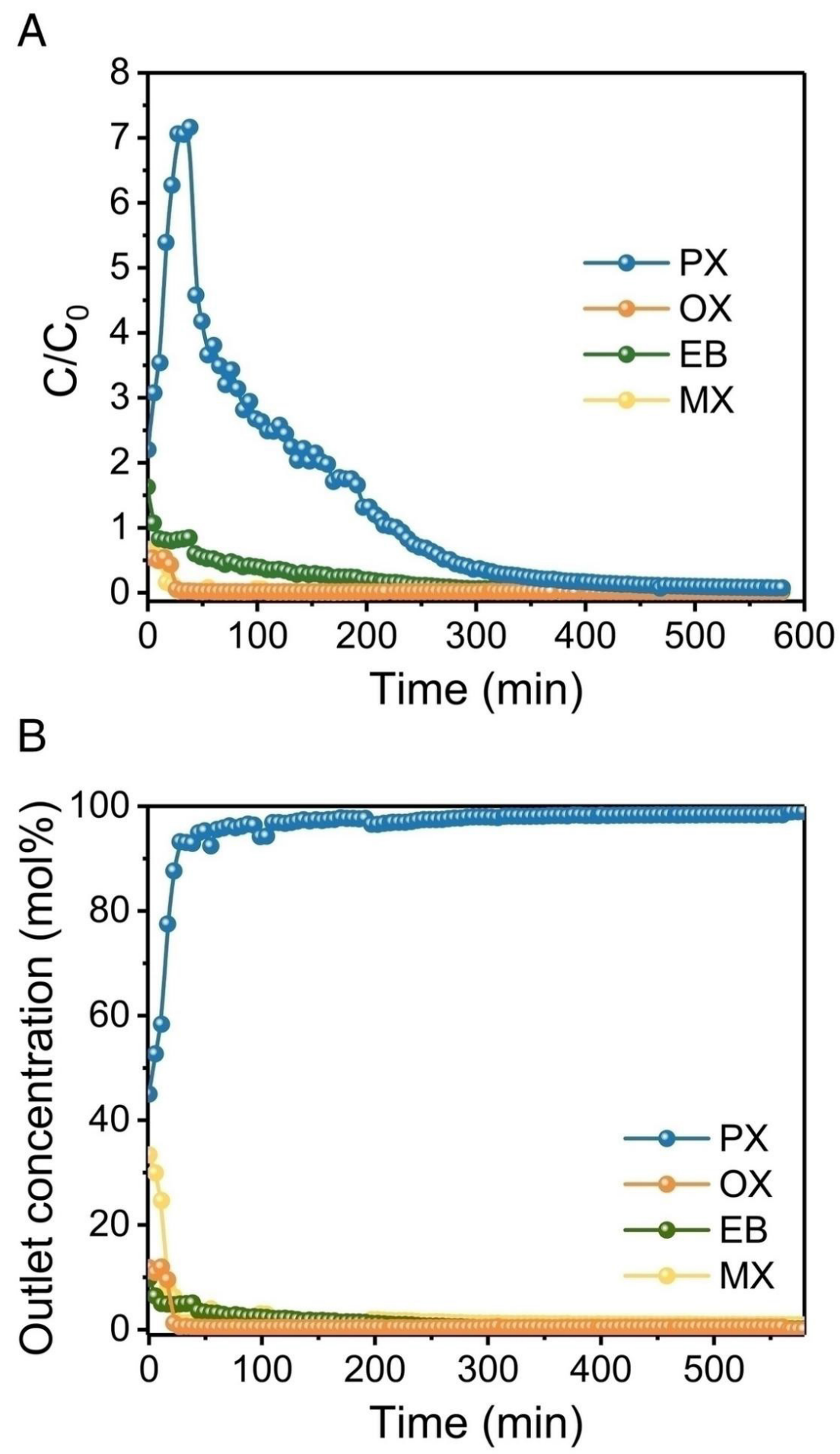

Fig. S31. Desorption curve from a breakthrough experiment using a quaternary mixture of C8 aromatics at 393 K with (A) normalized concentration and (B) outlet concentration as the y-axes. The desorption experiment was conducted at 423 K under a nitrogen flow.

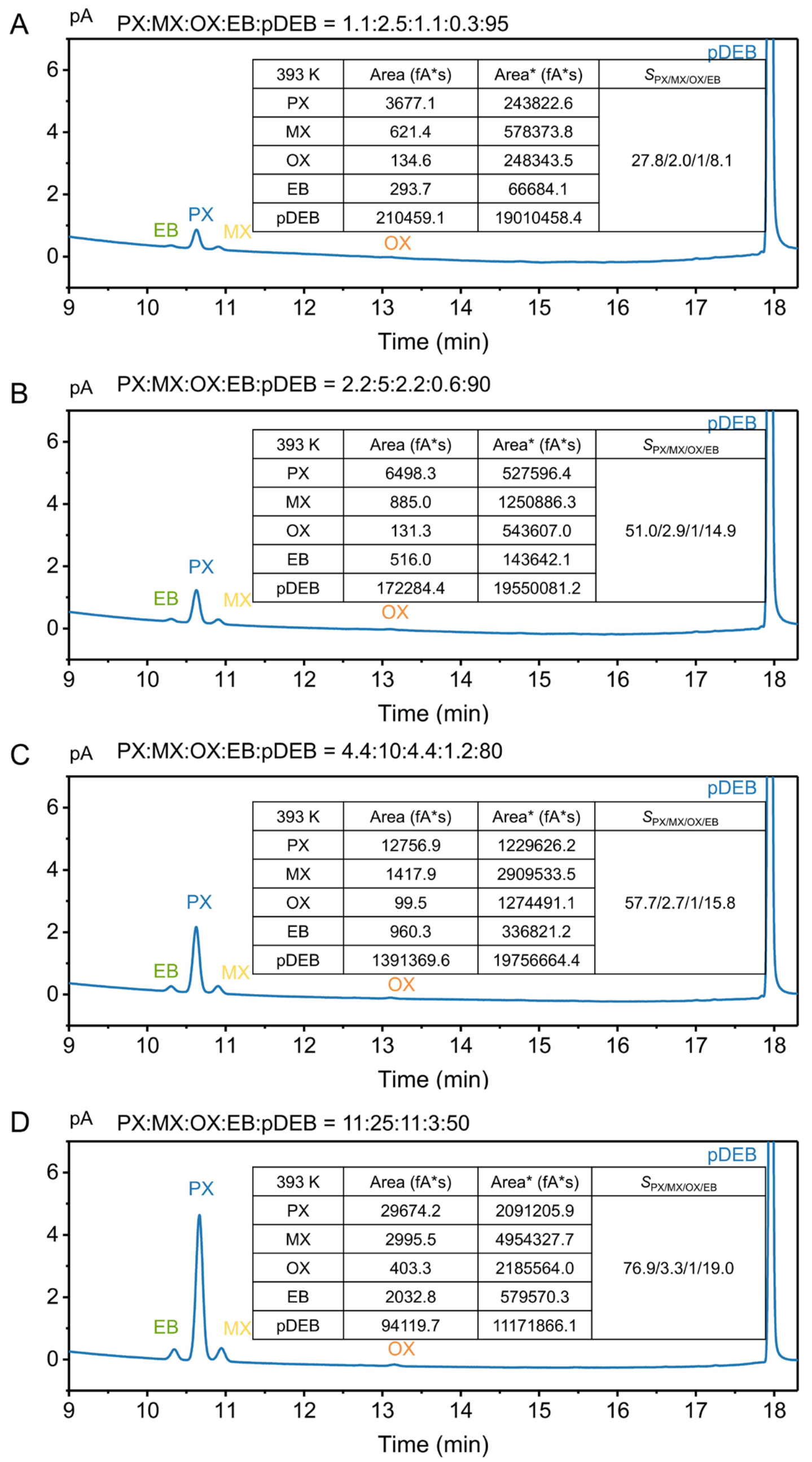

Fig. S32. Gas chromatograms for the solutions containing xylene isomers and pDEB, obtained with CH_2Cl_2 to extract from $Ni(BIC)_2$ decomposed by concentrated HCl solution. The $Ni(BIC)_2$ was previously exposed to a five-component mixture (PX/MX/OX/EB/pDEB) with mass composition ratios of (A) 1.1/2.5/1.1/0.3/95, (B) 2.2/5/2.2/0.6/90, (C) 4.4/10/4.4/1.2/80, and (D) 11/25/11/3/50 at 393 K. Note that Area* refers to the peak area of individual xylene or eluent components in the equilibrium solution.

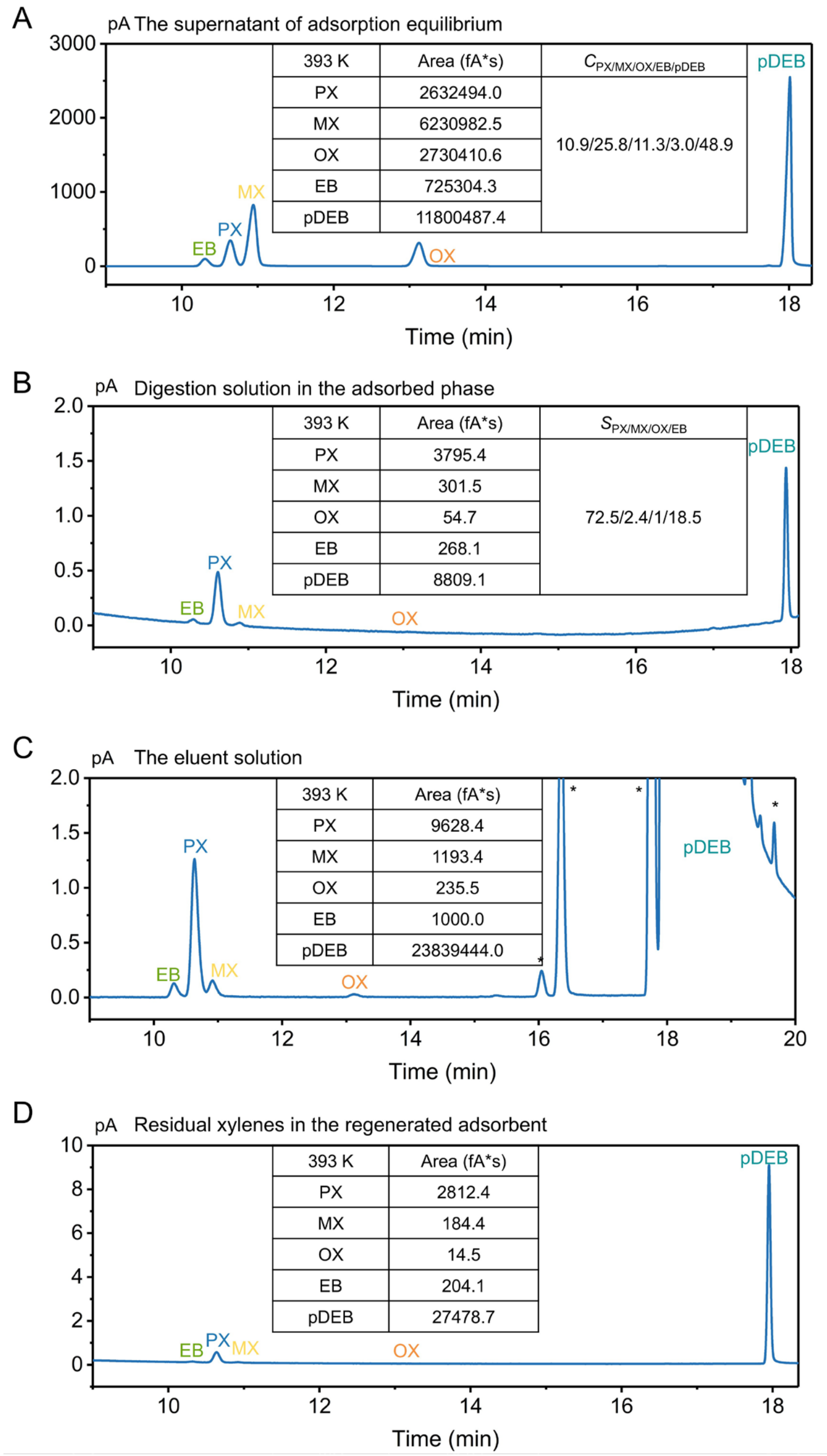

Fig. S33. Gas chromatograms for the xylene isomers and pDEB solution of adsorption-desorption experiments conducted at 393 K. Gas chromatograms for (A) the supernatant of adsorption equilibrium, (B) digestion solution in the adsorbed phase, (C) the eluent solution, and (D) residual xylenes in the regenerated adsorbent. Note that pDEB can also penetrate the pores of $Ni(BIC)_2$ and effectively elute the adsorbed xylenes, yielding a PX purity of 80%, with a recovery of 78% following a single-batch elution process.

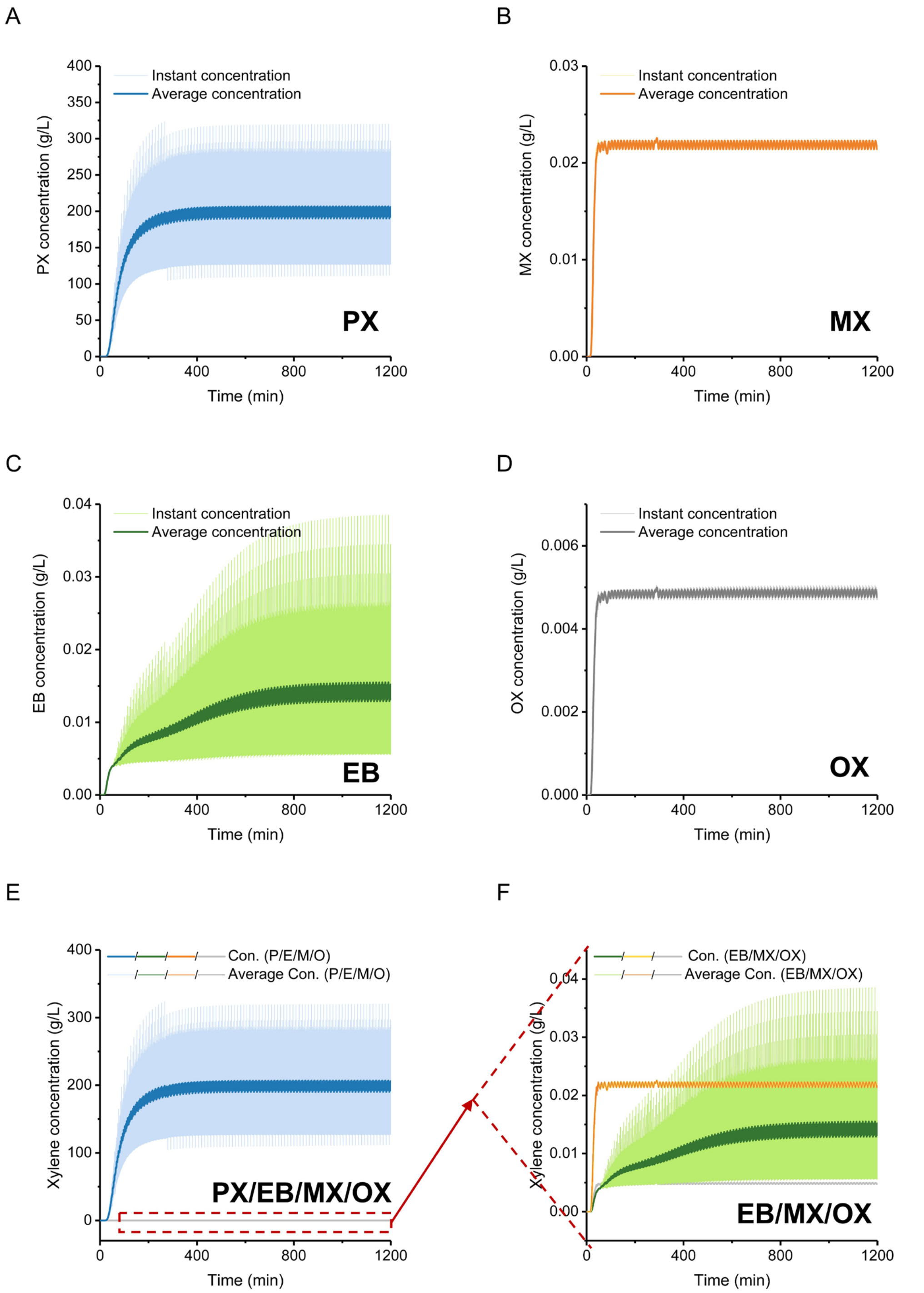

Fig. S34. Concentration-time curves of (A) PX, (B) MX, (C) EB, (D) OX, (E) PX/MX/EB/OX, and (F) MX/EB/OX in the extract outlet from the 24-column SMB simulation process on Ni(BIC)$_2$.

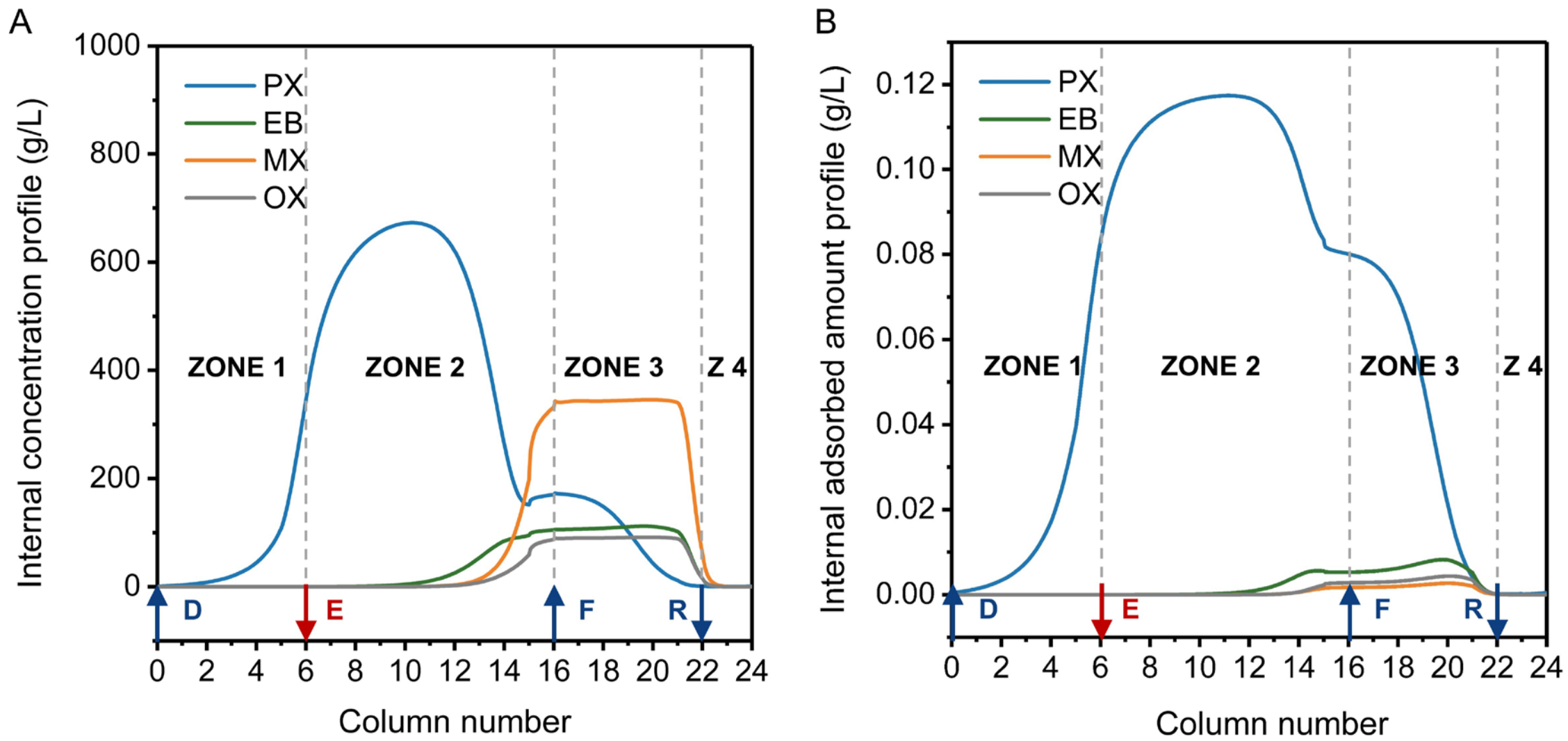

Fig. S35. Internal (A) concentration and (B) adsorbed amount profiles across the 24 columns at cyclic steady state of SMB simulation process on $Ni(BIC)_2$.

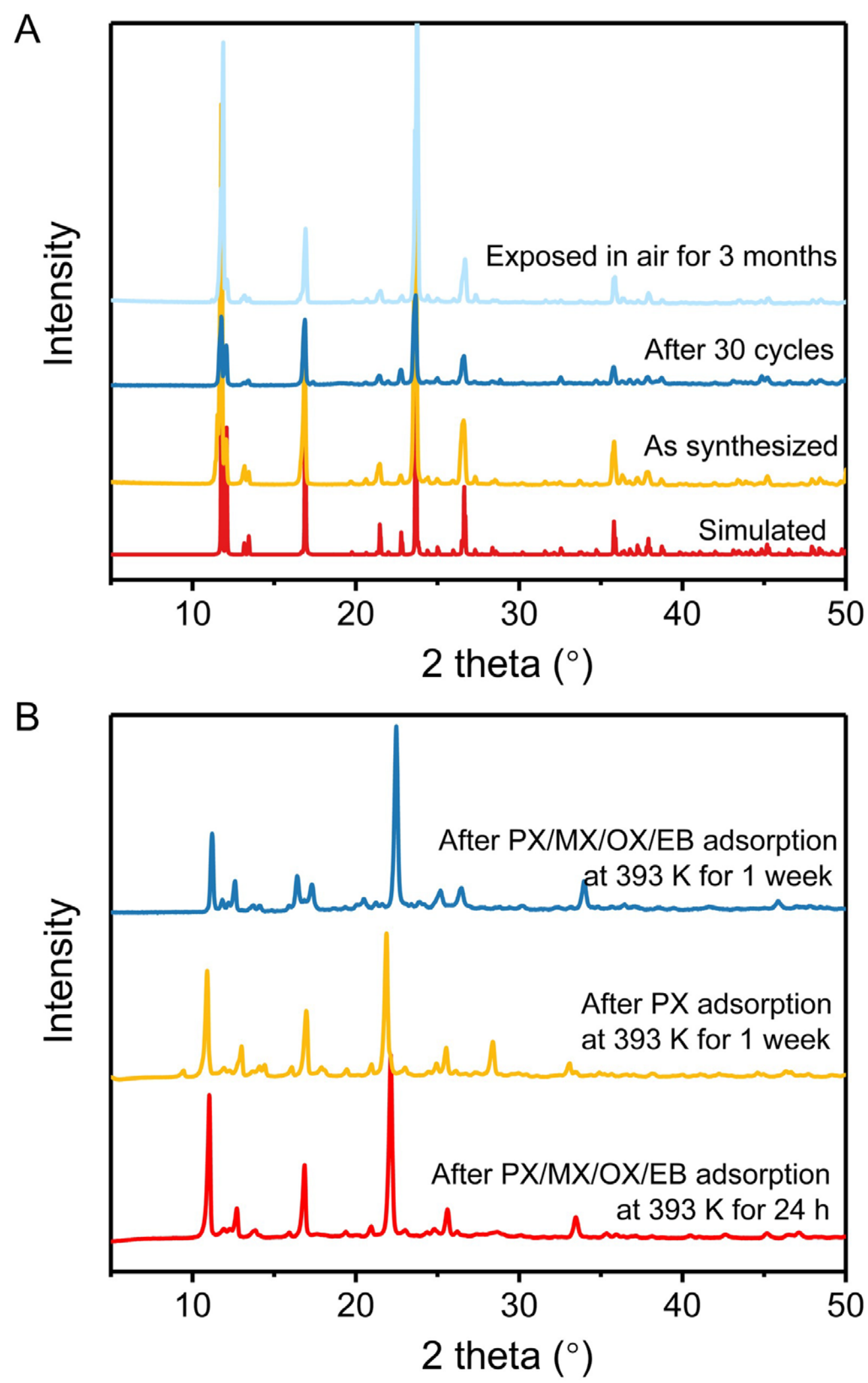

Fig. S36. Comparison of PXRD patterns for $Ni(BIC)_2$ treated under different conditions (A) PXRD patterns for $Ni(BIC)_2$ after exposure to air for 3 months, after 30 cycles, as synthesized, and as simulated. (B) PXRD patterns for $Ni(BIC)_2$ after immersion in PX and PX/MX/OX/EB mixture for one week and 24 h at 393 K.

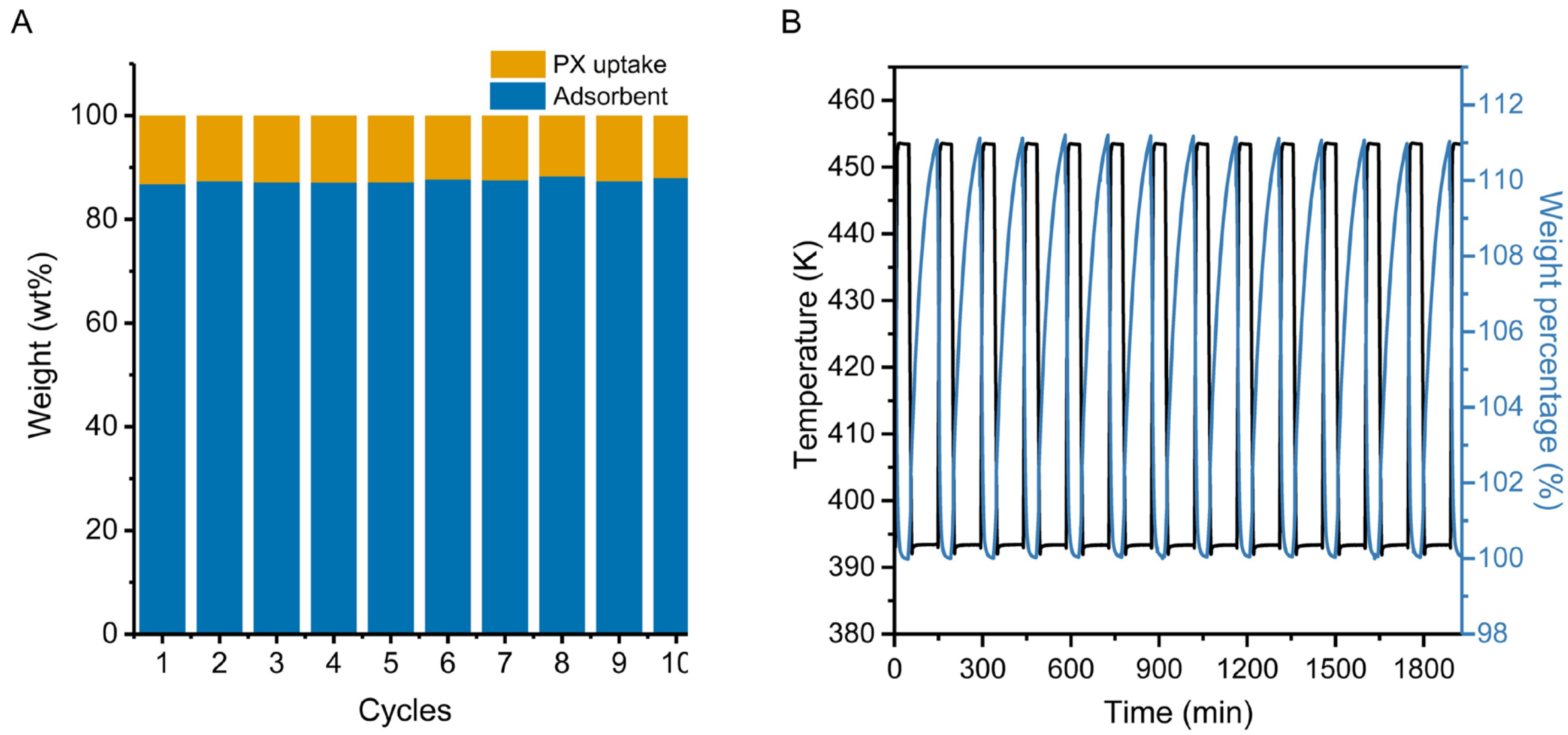

Fig. S37. (A) Recyclability tests of $Ni(BIC)_2$ in pure liquid-phase PX at 393 K. (B) Recyclability tests of $Ni(BIC)_2$ through vapor-phase adsorption at 393 K and desorption at 453 K.

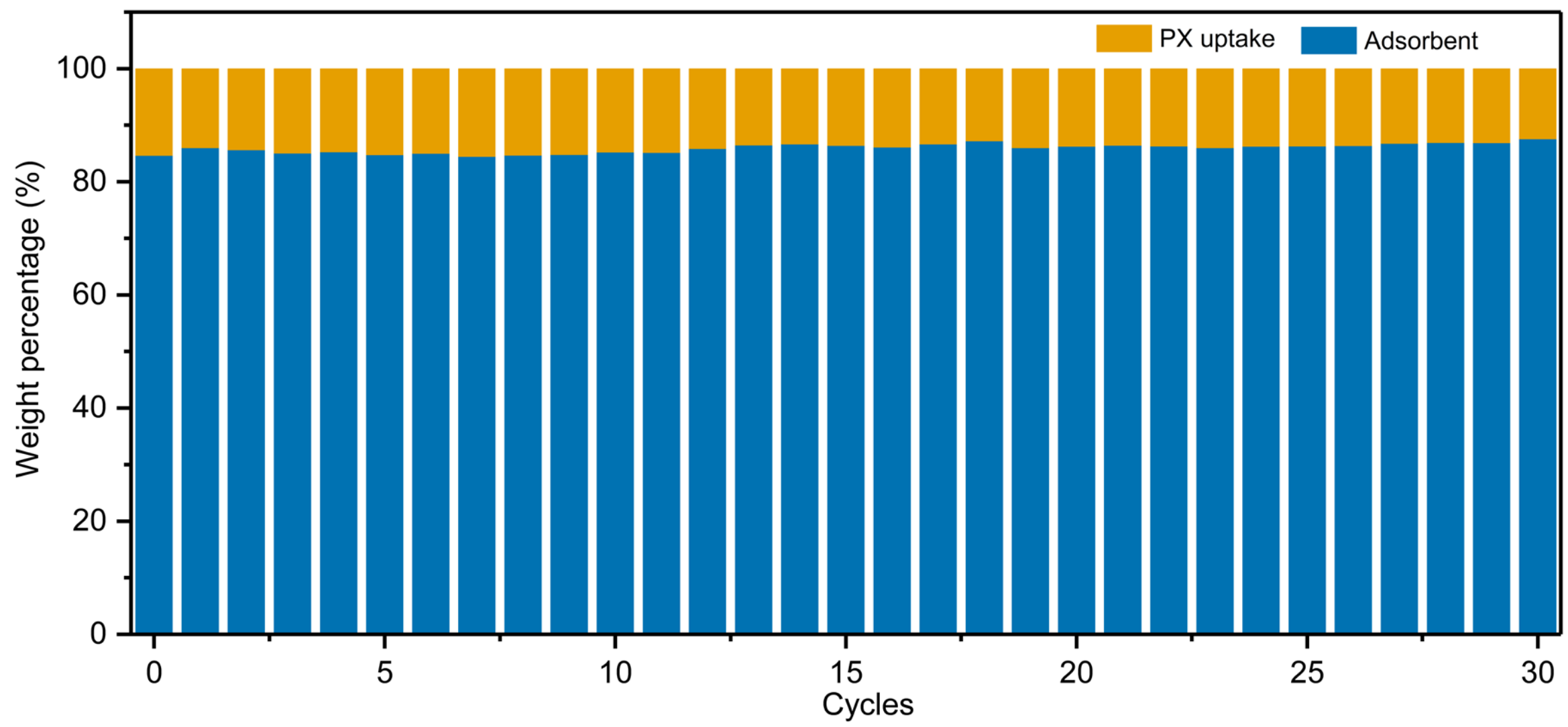

Fig. S38. Recyclability tests of $Ni(BIC)_2$ through liquid-phase adsorption at 393 K and desorption at 423 K.

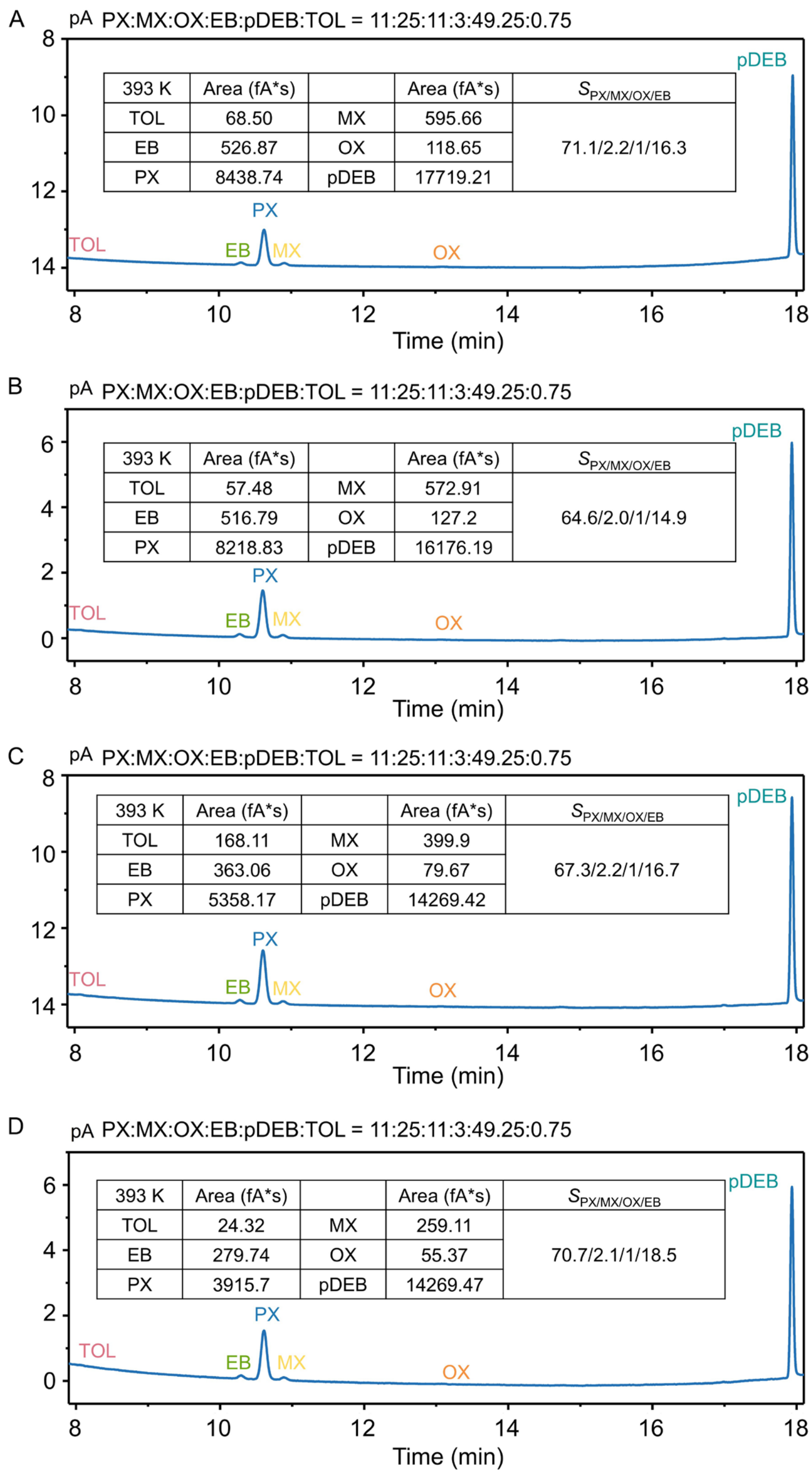

Fig. S39. Gas chromatograms for solutions containing xylene isomers, pDEB, and toluene, which were sampled in the long-term stability tests on $Ni(BIC)_2$. The adsorption selectivity of xylene isomers was calculated after the (A) 1st, (B) 10th, (C) 20th, and (D) 30th cycle.

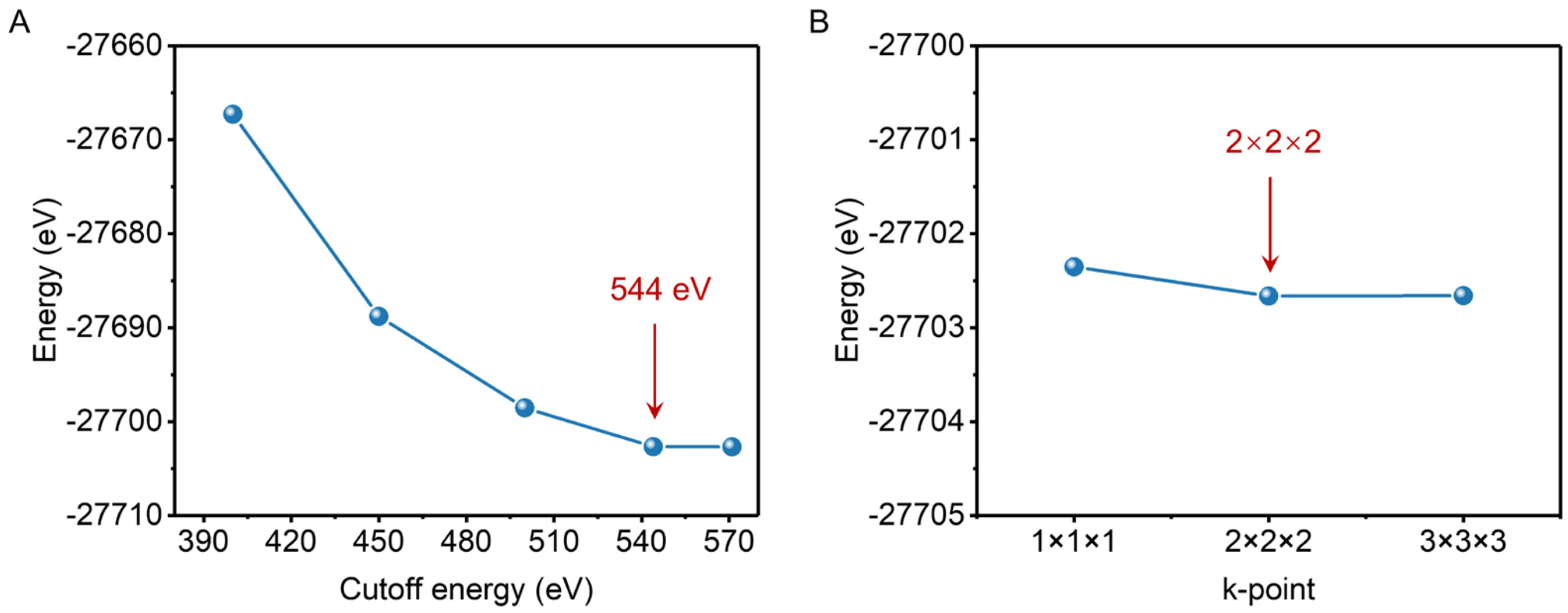

Fig. S40. Convergence tests for (A) cutoff energy and (B) k-point sampling.

Table S1. Physicochemical properties of C8 aromatics (*4*).

	**Boiling Point (K)**	**Kinetic Diameter (Å)**	**Polarizability ($\times 10^{-25}$ cm^3)**
para-xylene	411.5	5.8	137
meta-xylene	412.0	6.8	142
ortho-xylene	417.5	6.8	141
ethylbenzene	409.3	5.8	142

Table S2. Crystal data for as-synthesized **$Ni(BIC)_2$**, degassed **$Ni(BIC)_2$ (1)**, and post-adsorption test **$Ni(BIC)_2$ (2)**.

**Crystal**	**$Ni(BIC)_2$**	**$Ni(BIC)_2$ (1)**	**$Ni(BIC)_2$ (2)**
Formula	$C_{16}H_{10}N_4NiO_4$	$C_{16}H_{10}N_4NiO_4$	$C_{16}N_4NiO_4H_{10}$
Molecular weight (g mol^{-1})	380.97	380.97	380.97
Crystal system	tetragonal	tetragonal	orthorhombic
Space group	$P4_32_12$	$P4_32_12$	$C222_1$
a (Å)	14.9659(7)	15.260(2)	20.690(4)
b (Å)	14.9659(7)	15.260(2)	23.880(5)
c (Å)	8.3925(4)	8.2100(16)	7.8400(16)
α (°)	90	90	90
β (°)	90	90	90
γ (°)	90	90	90
Cell volume (Å^3)	1879.7(2)	1911.8(7)	3873.6(13)
Z	4	4	8
ρ_{calc} (g cm^{-3})	1.346	1.324	1.307
Index ranges	$-17 \leq h \leq 17$, $-17 \leq k \leq 17$ $-9 \leq l \leq 9$	$-18 \leq h \leq 18$ $-17 \leq k \leq 17$ $-9 \leq l \leq 9$	$-25 \leq h \leq 25$, $-29 \leq k \leq 29$ $-9 \leq l \leq 9$
μ/mm^{-1}	1.057	\	\
GooF	1.087	1.019	1.207
Final R [I>=2σ (I)]	$R_1 = 0.0208$ $wR_2 = 0.0597$	$R_1 = 0.0944$ $wR_2 = 0.2504$	$R_1 = 0.1871$ $wR_2 = 0.4155$
Testing method	SCXRD	MicroED	MicroED

Table S3. Summary of reported PX-selective adsorbents for the adsorptive separation of a mixture of xylene isomers.

Adsorbents		No. in Figure	Selectivity					Temp. (K)	PX uptake (mmol/g)	Ref.
			$S_{PX/EB}$	$S_{PX/OX}$	$S_{PX/MX}$	$S_{PX/(MX+OX)}$	$S_{PX/(MX+OX+EB)}$			
Faujazite-type zeolite	BaX	1	3.7	2.8	7.2	4.0	3.9	423	0.98^{L}	*12*[b]
Faujazite-type zeolite	Ba exchanged faujazite	-	NA	NA	NA	NA	NA	303-353	1.12^{L}	*57*[b], *68*[e], *69*[d]
Faujazite-type zeolite	Ba exchanged faujazite	-	NA	NA	3.6	NA	NA	393	1.05^{L}	
Faujazite-type zeolite	Ba exchanged faujazite	2	2.3 (450 K)	2.8 (450 K)	3.0 (450 K)	2.9	2.7	453	1.02^{L}	
Faujazite-type zeolite	KY	3	1.9	4.6	5.3	4.9	3.2	293	1.75^{L}	*61*[e]
Faujazite-type zeolite	KX	4	3.2	2.4	5.4	3.3	3.3	423	0.95^{L}	*70*[b]
ZSM-5		5	6.8	16.8	25.0	20.1	12.2	403	1.34^{L}	13[b]
MOF-monoclinic		6	5.2	4.6	2.5	3.2	3.7	393	0.12^{V} (423 K)	*71*[b]
Ce(HTCPB)$(EtOH)_{0.28}(H_2O)_{2.75}$*		7	2.4	5.6	4.5	5.0	3.7	298	1.23^{L}	*72*[d]
Cu(CDC)		8	5	10	7	8.2	6.8	298	1.18^{L}	*73*[d]
MIL-125(Ti)-NH_2		9	1.3 (343 K)	2.3 (343 K)	1.9 (343 K)	2.1	1.7	299-343	$5.80\text{-}3.60^{L}$	*74*[b]
DUT-8(Cu)		10	5.9	5.4	7.2	6.2	6.1	298	1.80^{L}	*75*[e]
AZO-Cage		11	9.7	15.6	10.9	12.8	11.6	298	1.17^{L}	*14*[a]
$Zn_2(aip)_2(bpe)\cdot 2DMF$		12	3.2	1.9	2.2	2.0	2.3	298	2.00^{V}	*76*[d]
$Zn_2(aip)_2(bpy)\cdot 2DMF$		13	3	16	5	7.6	5.0	298	1.77^{V}	
MIL-120(Al) (0.5 M)		14	3.6	11.5	8.3	9.6	6.2	298	1.45^{L}	*77*[e]
MIL-140B		15	1.6	1.8	2.1	1.9	1.8	323	1.60^{L}	*78*[d]
MOF-48		16	1.7	1.7	1.5	1.6	1.6	323	2.60^{L}	
$AgLClO_4$		17	10.4	24	6.2	9.9	10.0	383	NA	*17*[a]
$\{[Zn_4O(L)_3(DMF)_2].xG\}_n$**		18	6.3	50	11.8	19.1	11.4	298	2.86^{V}	*24*[a]
$Ni(ina)_2$		19	2	153	25	33.6	15.2	393	1.51^{V}	*79*[a]
HIAM-203		20	NA	378.8	860.5	526.0	NA	393	1.42^{V}	*80*[a]
ZIF-8		21	NA	3.9	1.6	2.3	NA	398	1.34^{L}	*81*[b]
MCF-50		22	NA	1.6	1.3	1.4	NA	453	NA	*82*[c]
EtP_6		23	NA	14.3	10.2	11.9	NA	298	0.93^{L}	19[d]
MFI zeolite		-	NA	104	NA	NA	NA	298	1.23^{V}	*83*[d]
Cu-metallocycle		24	NA	51.6	65.7	57.8	NA	293	1.32^{L}	*16*[d]
Mn-dhbq		25	NA	66.8 (393 K)	25.5 (393 K)	36.9	NA	303-393	$1.96\text{-}1.51^{V}$	*25*[d]
MAF-89		26	NA	221	46	76.1	NA	308	2.00^{L}	*10*[d]
MAF-X8		-	NA	NA	NA	NA	NA	433	2.10	*84*[a]

Co-CUK-1	-	NA	NA	NA	NA	NA	323	2.10	*40*[a]
Mg-CUK-1	-	NA	NA	NA	NA	NA	323	1.50	*85*[a]
Mn-dhbq (Breakthrough)	27	1.4$_{(393\ K)}$	47.8$_{(393\ K)}$	24.0$_{(393\ K)}$	28.3	11.4	303-393	$1.96\text{-}1.51^{V}$	This work[b]
RIGWIP	28	NA	73.4	36.0	42.8	NA	453	0.64^{S}	*86*[f]
NEKREB	29	NA	39.2	16.9	20.5	NA	453	0.96^{S}	*86*[f]
ja308995t_si_002	30	NA	6.4	16.4	11.0	NA	453	1.25^{S}	*86*[f]
RACZEC	31	NA	2.8	16.4	6.5	NA	453	0.82^{S}	*86*[f]
AGARUW	32	NA	4.0	9.1	6.5	NA	453	0.94^{S}	*86*[f]
BAXSIE	33	NA	1014.4	4.5	6.5	NA	453	0.84^{S}	*86*[f]
UWODAN	34	NA	3.0	7.7	5.2	NA	453	0.85^{S}	*86*[f]
YORNIE	35	NA	4.1	4.5	4.5	NA	453	1.97^{S}	*86*[f]
UTULUR	36	NA	2.5	5.5	4.0	NA	453	0.87^{S}	*86*[f]
MAGBON	37	NA	3.6	3.9	3.8	NA	453	1.52^{S}	*86*[f]
JIVFUQ	38	NA	NA	NA	46.9	NA	373	2.78^{S}	*87*[f]
GAYFOD	39	NA	NA	NA	5.4	NA	373	1.55^{S}	*87*[f]
RACZEC	40	NA	NA	NA	16.2	NA	373	1.17^{S}	*87*[f]
hMOF-24691	-	NA	NA	NA	NA	2.71	373	1.35^{S}	*88*[f]
Ni(BIC)$_2$ (Liquid phase)	-	2.4	21.4	10.1	12.0	9.2	303	$1.78^{L}$$_{(393\ K)}$	This work[d]
	-	2.7	29.0	12.8	15.4	11.3	333		
	-	2.8	34.8	13.4	16.5	12.0	363		
	-	3.1	56.1	21.8	26.8	16.9	393		
Ni(BIC)$_2$ (Breakthrough)	-	11.7$_{(393\ K)}$	116.9$_{(393\ K)}$	38.8$_{(393\ K)}$	48.8	39.2	303-393	$1.82\text{-}1.86^{V}$	This work[b]

[*]Abbreviated as Ce-MOF.

[**]Abbreviated as Zn-MOF.

Superscripts L, V, and S denote PX uptake capacities obtained from liquid-phase experiments, vapor-phase experiments, and simulations, respectively.

For each material, only the highest/best values of selectivity were selected, regardless of whether the experiments were conducted with adsorbate in liquid or vapor phase.

[a]Calculated from vapor-phase sorption experiments. [b]Calculated from experimental breakthrough curves. [c]Calculated from chromatographic separation data. [d]Calculated from batch solid-liquid sorption experiments. [e]Calculated from liquid-phase adsorption isotherms. [f]Calculated from multicomponent GCMC simulations.

Table S4. Extended Langmuir parameters (q_m and b values) used in the Δq calculations for both $Ni(BIC)_2$ and Mn-dhbq.

Adsorbents	Compound (i)	$q_{sat,i}$ (mmol/g)	b (kPa^{-1})	R^2
$Ni(BIC)_2$	PX	2.44E+00	4.31E+04	0.9263
	MX	1.98E+13	2.77E-11	0.9095
	OX	2.37E+14	1.94E-12	0.8964
	EB	6.47E+13	1.58E-10	0.9737
Mn-dhbq	PX	1.35E+00	2.15E+01	0.9803
	MX	2.47E-01	1.23E+00	0.9787
	OX	8.03E-01	3.03E-01	0.9733
	EB	2.55E+00	8.25E-01	0.9654

Table S5. Diffusion mass transfer coefficient ($k_s a_p$, s^{-1}) obtained from experiments on vapor-phase adsorption kinetics.

Adsorbent	Adsorbate	Temperature (K)	$k_s a_p$ (s^{-1})
Ni(BIC)$_2$	PX	303	6.49×10^{-5}
	MX	303	4.14×10^{-6}
	PX	393	5.83×10^{-4}
ADS-37	PX	393	2.09×10^{-5}

Table S6. Liquid-phase adsorption selectivity for binary, ternary, and quaternary liquid mixtures on $Ni(BIC)_2$ at temperatures of 303, 333, 363, and 393 K, and corresponding overall xylene capacity at 393 K.

Constituent	303 K	333 K	363 K	393 K	
				Selectivity	Xylene capacity (mmol g^{-1})
PX/MX	10.1/1	12.8/1	13.4/1	21.8/1	1.70
PX/OX	21.4/1	29.0/1	34.8/1	56.1/1	1.73
PX/EB	2.4/1	2.7/1	2.8/1	3.1/1	1.76
P/M/O	32.8/3.4/1	55.9/4.2/1	68.7/5.0/1	77.1/4.4/1	1.64
P/M/O/E	57.8/3.8/1/23.3	99.5/6.0/1/35.8	106.0/7.6/1/40.5	155.5/7.1/1/49.0	1.69

Table S7. Stuart model parameters correlated from liquid-phase adsorption equilibrium data for binary mixtures of PX/MX, PX/OX, and PX/EB on $Ni(BIC)_2$ at 333 and 393 K.

333 K	**PX/EB**	**PX/MX**	**PX/OX**
M	0.83569	0.45476	0.27015
N	0.33825	0.95183	1.47179
ϕ	54.71523	181.56492	243.04005
K	112.63725	316.95939	490.10607
R^2	0.99743	0.99968	0.9998
Mean error	2.6%	0.44%	0.31%

393 K	**PX/EB**	**PX/MX**	**PX/OX**
M	0.82391	0.95467	0.31238
N	0.52893	1.77275	1.90146
ϕ	69.20337	17.81469	270.23466
K	207.86949	696.69075	747.27378
R^2	0.99723	0.99727	0.768
Mean error	3.3%	1.8%	0.61%

Table S8. DFT-calculated thermodynamic energies of xylene isomers' adsorption on $Ni(BIC)_2$.

ΔE ($kJ\ mol^{-1}$)	PX	EB	MX	OX
ΔE_{def}	81.1			
ΔE_{int}	- 104.0	- 86.2	- 81.7	- 71.7
ΔE_{tot}	- 22.9	- 5.1	- 0.6	9.4

Table S9. Molecular dimensions (x, y, and z), minimum dimensions (MIN-1, MIN-2), and aspect ratio z/x for xylene isomers and pDEB. (*44*)

	x	***y***	***z***	**MIN-1**	**MIN-2**	**Aspect ratio *z/x***
PX	6.618	3.810	9.146	3.810	6.618	1.38
MX	8.994	3.949	7.315	3.949	7.258	1.23
OX	7.269	3.834	7.826	3.834	7.269	1.08
EB	6.625	5.285	9.361	5.285	6.625	1.41
pDEB	6.682	4.895	11.960	4.895	6.682	1.79

Table S10. Porosity and Langmuir model parameters of $Ni(BIC)_2$ and FAU zeolite for xylene isomers, used in SMB process simulations.

Parameters	$Ni(BIC)_2$	FAU Zeolite
ε_b	0.37	0.39
ε_p	0.2	0.1
ε_t	0.496	0.451
$q_{m,PX}$ (kg/kg)	0.1203	0.1303
$q_{m,MX}$ (kg/kg)	0.02422	0.1303
$q_{m,OX}$ (kg/kg)	0.01157	0.1303
$q_{m,EB}$ (kg/kg)	0.1176	0.1303
$q_{m,PDEB}$ (kg/kg)	0.1163	0.1077
K_{PX} (m^3/kg)	0.16883	1.0658
K_{MX} (m^3/kg)	0.01476	0.2299
K_{OX} (m^3/kg)	0.07179	0.1884
K_{EB} (m^3/kg)	0.01837	0.3067
K_{PDEB} (m^3/kg)	0.09557	1.2935
Data resources	Liquid-phase adsorption isotherms and pulse chromatography experiments in this work	Reported by Rodrigues (*46, 47*).

Note: ε_b = bed porosity; ε_p = particle porosity; ε_t = total porosity; $q_{m,i}$ = saturation capacity of component *i* ($kg\cdot kg^{-1}$ adsorbent); b_i = Langmuir equilibrium constant of component *i* ($L\cdot g^{-1}$)

Table S11. Axial dispersion coefficient (D_{ax}) and mass-transfer coefficient (*MTC*) of xylene isomers on Ni(BIC)$_2$, obtained by fitting pulse chromatography experimental data.

**Parameters**	**Ni(BIC)$_2$**
D_{ax} (cm^2 min^{-1})	0.002
MTC_{PX} (min^{-1})	1.95
MTC_{MX} (min^{-1})	3.95
MTC_{OX} (min^{-1})	3.41
MTC_{EB} (min^{-1})	2.18
MTC_{PDEB} (min^{-1})	7.89

Table S12. SMB unit geometry and operating conditions for process simulations on $Ni(BIC)_2$ and FAU zeolite, along with the corresponding simulation performance.

Parameters		$Ni(BIC)_2$ (12 beds)	$Ni(BIC)_2$ (24 beds)	FAU zeolite (24 beds)
SMB unit geometry	Column length (m)	1.14	1.14	1.14
	Column diameter (m)	4.12	4.12	4.12
	Number of columns	12	24	24
	Number of zones	4	4	4
Operating conditions	Configuration	3/5/3/1	6/10/6/2	6/9/6/3
	Feed composition	PX/MX/OX/EB = 23.6/49.7/12.7/14.0 (wt%)		
	Temperature (°C)	120	120	180
	Pressure (bar)	9	9	9
	Switching time (min)	1.35	1.27	1.15
	Q_D (m^3/h)	309.0	300.0	173.4
	Q_E (m^3/h)	180.0	190.8	99.0
	Q_F (m^3/h)	120.0	214.2	87.0
	Q_R (m^3/h)	249.0	323.4	161.4
	Q_1 (m^3/h)	526.8	517.8	496.8
Performance	Purity (%)	99.21	99.98	98.90
	Recovery (%)	99.99	99.95	99.79
	SC ($m^3 \cdot kg^{-1} \cdot PX$)	0.0133	0.0076	0.0117
	Production (g $PX \cdot h^{-1} \cdot L^{-1}$)	251.98	214.78	66.56
	Processing capacity (m^3 $xylene \cdot h^{-1} \cdot m^{-3}$)	1.3055	1.1652	0.3910
Data resources		This work		Unit geometry and conditions from Rodrigues (*46, 47*). Performances from simulations in this work.

Table S13. Comparison of operating parameters, stream results, and energy performance based on Aspen simulations of PX/PDEB separation, using extract streams from the 24-bed SMB processes based on $Ni(BIC)_2$ and FAU adsorbents as feeds.

	Parameters	$Ni(BIC)_2$	FAU zeolite
Operating parameters	Number of plates	35	35
	Feed stage	17	17
	Feed temperature (°C)	120	180
	Feed pressure (bar)	1.11	1.11
	Feed flow rate (kg h^{-1})	2512.2	1214.7
	Feed concentration (kg m^{-3})	206.9 (PX)<br>589.5 (PDEB)	149.6 (PX)<br>586.6 (PDEB)
	Reflux ratio	1.47	2.46
Stream results	Distillate flow rate (PX) (kg h^{-1})	594.2	221.3
	PX purity in distillate	99.9% (wt%)	99.9% (wt%)
	Bottoms flow rate (PDEB) (kg h^{-1})	1684.8	880.6
	PDEB purity in distillate	99.1% (wt%)	99.8% (wt%)
Energy performance	Condenser heat duty (MJ h^{-1})	500.1	261.0
	Reboiler heat duty (MJ h^{-1})	753.5	299.1
	Energy consumption (MJ kg^{-1} PX)	2.11	2.53

Table S14. The values of A, B, and C for xylene isomers from the Antoine equation.

Compound	A	B	C
PX	6.99052	1453.430	215.307
MX	7.00908	1462.266	215.105
EB	6.95719	1424.255	213.206
OX	6.99891	1474.679	213.686

* The units for temperature (*T*) and pressure (*P*) in Antoine equation $\left(\log(P) = \mathrm{A} - \frac{B}{T+C}\right)$ are ℃ and mmHg, respectively.

Table S15. K-point convergence at 544 eV.

Cutoff (eV)	k-point	Energy (eV)	ΔE vs previous (eV)
544	1×1×1	-27702.35255	-
544	2×2×2	-27702.66302	-0.31047
544	3×3×3	-27702.65946	+0.00356

Table S16. Cutoff convergence at 2×2×2.

Cutoff (eV)	k-point	Energy (eV)	ΔE vs previous (eV)
400	2×2×2	-27667.29546	-
450	2×2×2	-27688.80143	-21.50597
500	2×2×2	-27698.53056	-9.72913
544	2×2×2	-27702.66302	-4.13246
571	2×2×2	-27702.68053	-0.01751

Data S1.

Crystallographic structures of as-synthesized **Ni(BIC)$_2$** (2266103), activated **Ni(BIC)$_2$ (1)** (2266102), and xylene-adsorbed **Ni(BIC)$_2$ (2)** (2266101).

Data S2.

Python scripts for numerical fitting of mass-transfer kinetics.

REFERENCES

1. J. Fabri, U. Graeser, T. A. Simo, *Ullman's Encyclopedia of Industrial Chemistry* (Wiley-VCH, 2000).

2. G. Zhang, Y. Ding, A. Hashem, A. Fakim, N. M. Khashab, Xylene isomer separations by intrinsically porous molecular materials. *Cell Rep. Phys. Sci.* **2**, 100470 (2021).

3. D. S. Sholl, R. P. Lively, Seven chemical separations to change the world. *Nature* **532**, 435–437 (2016).

4. Z. Zhang, S. B. Peh, C. Kang, K. Chai, D. Zhao, Metal-organic frameworks for C6-C8 hydrocarbon separations. *EnergyChem* **3**, 100057 (2021).

5. R. Krishna, Separating mixtures by exploiting molecular packing effects in microporous materials. *Phys. Chem. Chem. Phys.* **17**, 39–59 (2015).

6. M. Minceva, A. E. Rodrigues, Understanding and revamping of industrial scale SMB units for p-xylene separation. *AIChE J.* **53**, 138–149 (2007).

7. Q. Shi, J. C. Gonçalves, A. F. P. Ferreira, A. E. Rodrigues, A review of advances in production and separation of xylene isomers. *Chem. Eng. Process.* **169**, 108603 (2021).

8. Y. Yang, P. Bai, X. Guo, Separation of xylene isomers: A review of recent advances in materials. *Ind. Eng. Chem. Res.* **56**, 14725–14753 (2017).

9. L. S. Cheng, J. A. Johnson, "Adsorbents with improved mass transfer properties and their use in the adsorptive separation of para-xylene," US Patent 8,609,925 (2013).

10. Z.-M. Ye, X.-F. Zhang, D.-X. Liu, Y.-T. Xu, C. Wang, K. Zheng, D.-D. Zhou, C.-T. He, J.-P. Zhang, A gating ultramicroporous metal-organic framework showing high adsorption selectivity, capacity and rate for xylene separation. *Sci. China Chem.* **65**, 1552–1558 (2022).

11. C. Mellot, M.-H. Simonot-Grange, E. Pilverdier, J.-P. Bellat, D. Espinat, Adsorption of gaseous p- or m-xylene in BaX zeolite: Correlation between thermodynamic and crystallographic studies. *Langmuir* **11**, 1726–1730 (1995).

12. M. Rasouli, N. Yaghobi, F. Allahgholipour, H. Atashi, Para-xylene adsorption separation process using nano-zeolite Ba-X. *Chem. Eng. Res. Des.* **92**, 1192–1199 (2014).

13. M. Rasouli, N. Yaghobi, S. Chitsazan, M. H. Sayyar, Influence of monovalent cations ion-exchange on zeolite ZSM-5 in separation of para-xylene from xylene mixture. *Micropor. Mesopor. Mater.* **150**, 47–54 (2012).

14. B. Moosa, L. O. Alimi, A. Shkurenko, A. Fakim, P. M. Bhatt, G. Zhang, M. Eddaoudi, N. M. Khashab, A polymorphic azobenzene cage for energy-efficient and highly selective p-xylene separation. *Angew. Chem. Int. Ed. Engl.* **59**, 21367–21371 (2020).

15. M. Lusi, L. J. Barbour, Solid-vapor sorption of xylenes: Prioritized selectivity as a means of separating all three isomers using a single substrate. *Angew. Chem. Int. Ed. Engl.* **51**, 3928–3931 (2012).

16. M. du Plessis, V. I. Nikolayenko, L. J. Barbour, Record-setting selectivity for p-xylene by an intrinsically porous zero-dimensional metallocycle. *J. Am. Chem. Soc.* **142**, 4529–4533 (2020).

17. N. Sun, S.-Q. Wang, R. Zou, W.-G. Cui, A. Zhang, T. Zhang, Q. Li, Z.-Z. Zhuang, Y.-H. Zhang, J. Xu, M. J. Zaworotko, X.-H. Bu, Benchmark selectivity p-xylene separation by a non-porous molecular solid through liquid or vapor extraction. *Chem. Sci.* **10**, 8850–8854 (2019).

18. G. Zhang, A.-H. Emwas, U. F. S. Hameed, S. T. Arold, P. Yang, A. Chen, J.-F. Xiang, N. M. Khashab, Shape-induced selective separation of ortho-substituted benzene isomers enabled by cucurbit[7]uril host macrocycles. *Chem* **6**, 1082–1096 (2020).

19. K. Jie, M. Liu, Y. Zhou, M. A. Little, A. Pulido, S. Y. Chong, A. Stephenson, A. R. Hughes, F. Sakakibara, T. Ogoshi, F. Blanc, G. M. Day, F. Huang, A. I. Cooper, Near-ideal xylene selectivity in adaptive molecular pillar[*n*]arene crystals. *J. Am. Chem. Soc.* **140**, 6921–6930 (2018).

20. A. Schneemann, V. Bon, I. Schwedler, I. Senkovska, S. Kaskel, R. A. Fischer, Flexible metal-organic frameworks. *Chem. Soc. Rev.* **43**, 6062–6096 (2014).

21. J. R. Li, R. J. Kuppler, H. C. Zhou, Selective gas adsorption and separation in metal-organic frameworks. *Chem. Soc. Rev.* **38**, 1477–1504 (2009).

22. L. Alaerts, C. E. A. Kirschhock, M. Maes, M. A. van der Veen, V. Finsy, A. Depla, J. A. Martens, G. V. Baron, P. A. Jacobs, J. F. M. Denayer, D. E. De Vos, Selective adsorption and separation of xylene isomers and ethylbenzene with the microporous vanadium(IV) terephthalate MIL-47. *Angew. Chem. Int. Ed. Engl.* **46**, 4293–4297 (2007).

23. K. B. Idrees, Z. Li, H. Xie, K. O. Kirlikovali, M. Kazem-Rostami, X. Wang, X. Wang, T.-Y. Tai, T. Islamoglu, J. F. Stoddart, R. Q. Snurr, O. K. Farha, Separation of aromatic hydrocarbons in porous materials. *J. Am. Chem. Soc.* **144**, 12212–12218 (2022).

24. S. Mukherjee, B. Joarder, B. Manna, A. V. Desai, A. K. Chaudhari, S. K. Ghosh, Framework-flexibility driven selective sorption of p-xylene over other isomers by a dynamic metal-organic framework. *Sci. Rep.* **4**, 5761 (2014).

25. L. Li, L. Guo, D. H. Olson, S. Xian, Z. Zhang, Q. Yang, K. Wu, Y. Yang, Z. Bao, Q. Ren, J. Li, Discrimination of xylene isomers in a stacked coordination polymer. *Science* **377**, 335–339 (2022).

26. X. Yang, H.-L. Zhou, C.-T. He, Z.-W. Mo, J.-W. Ye, X.-M. Chen, J.-P. Zhang, Flexibility of metal-organic framework tunable by crystal size at the micrometer to submillimeter scale for efficient xylene isomer separation. *Research* **2019**, 9463719 (2019).

27. Q. Dong, X. Zhang, S. Liu, R.-B. Lin, Y. Guo, Y. Ma, A. Yonezu, R. Krishna, G. Liu, J. Duan, R. Matsuda, W. Jin, B. Chen, Tuning gate-opening of a flexible metal-organic framework for ternary gas sieving separation. *Angew. Chem. Int. Ed. Engl.* **59**, 22756–22762 (2020).

28. X. Zhang, D. Zhou, J. Zhang, Tuning the gating energy barrier of metal-organic framework for molecular sieving. *Chem* **7**, 1006–1019 (2021).

29. K.-J. Chen, D. G. Madden, S. Mukherjee, T. Pham, K. A. Forrest, A. Kumar, B. Space, J. Kong, Q.-Y. Zhang, M. J. Zaworotko, Synergistic sorbent separation for one-step ethylene purification from a four-component mixture. *Science* **366**, 241–246 (2019).

30. Y. Su, K.-i. Otake, J.-J. Zheng, S. Horike, S. Kitagawa, C. Gu, Separating water isotopologues using diffusion-regulatory porous materials. *Nature* **611**, 289–294 (2022).

31. H. Zeng, M. Xie, T. Wang, R.-J. Wei, X.-J. Xie, Y. Zhao, W. Lu, D. Li, Orthogonal-array dynamic molecular sieving of propylene/propane mixtures. *Nature* **595**, 542–548 (2021).

32. J. A. Mason, J. Oktawiec, M. K. Taylor, M. R. Hudson, J. Rodriguez, J. E. Bachman, M. I. Gonzalez, A. Cervellino, A. Guagliardi, C. M. Brown, P. L. Llewellyn, N. Masciocchi, J. R. Long, Methane storage in flexible metal-organic frameworks with intrinsic thermal management. *Nature* **527**, 357–361 (2015).

33. D. Antypov, A. Shkurenko, P. M. Bhatt, Y. Belmabkhout, K. Adil, A. Cadiau, M. Suyetin, M. Eddaoudi, M. J. Rosseinsky, M. S. Dyer, Differential guest location by host dynamics enhances propylene/propane separation in a metal-organic framework. *Nat. Commun.* **11**, 6099 (2020).

34. M. I. Gonzalez, M. T. Kapelewski, E. D. Bloch, P. J. Milner, D. A. Reed, M. R. Hudson, J. A. Mason, G. Barin, C. M. Brown, J. R. Long, Separation of xylene isomers through multiple metal site interactions in metal-organic frameworks. *J. Am. Chem. Soc.* **140**, 3412–3422 (2018).

35. X. Li, J. Wang, N. Bai, X. Zhang, X. Han, I. da Silva, C. G. Morris, S. Xu, D. M. Wilary, Y. Sun, Y. Cheng, C. A. Murray, C. C. Tang, M. D. Frogley, G. Cinque, T. Lowe, H. Zhang, A. J. Ramirez-Cuesta, K. M. Thomas, L. W. Bolton, S. Yang, M. Schröder, Refinement of pore size at sub-angstrom precision in robust metal-organic frameworks for separation of xylenes. *Nat. Commun.* **11**, 4280 (2020).

36. S.-Q. Wang, S. Mukherjee, E. Patyk-Kaźmierczak, S. Darwish, A. Bajpai, Q.-Y. Yang, M. J. Zaworotko, Highly selective, high-capacity separation of o-xylene from C8 aromatics by a switching adsorbent layered material. *Angew. Chem. Int. Ed. Engl.* **131**, 6702–6706 (2019).

37. D. D. Zhou, J. P. Zhang, On the role of flexibility for adsorptive separation. *Acc. Chem. Res.* **55**, 2966–2977 (2022).

38. S. Krause, N. Hosono, S. Kitagawa, Chemistry of soft porous crystals: Structural dynamics and gas adsorption properties. *Angew. Chem. Int. Ed. Engl.* **59**, 15325–15341 (2020).

39. L. Wu, M. Xue, L. Huang, S. L. Qiu, Hydrothermal synthesis, structure, fluorescence property of a novel 3D metal-organic framework with high thermal stability. *Sci. China Chem.* **54**, 1441–1445 (2011).

40. R. Krishna, Screening metal-organic frameworks for mixture separations in fixed-bed adsorbers using a combined selectivity/capacity metric. *RSC Adv.* **7**, 35724–35737 (2017).

41. J. W. Yoon, J. S. Lee, G. W. Piburn, K. H. Cho, K. Jeon, H. K. Lim, H. Kim, C. H. Jun, S. M. Humphrey, R. Krishna, J. S. Chang, Highly selective adsorption of p-xylene over other C8 aromatic hydrocarbons by Co-CUK-1: A combined experimental and theoretical assessment. *Dalton Trans.* **46**, 16096–16101 (2017).

42. E. Glueckauf, Theory of chromatography. Part 10.—Formulæ for diffusion into spheres and their application to chromatography. *Trans. Faraday Soc.* **51**, 1540–1551 (1955).

43. E. B. Stuart, J. Coull, Liquid-phase adsorption equilibria. *AIChE J.* **4**, 383–388 (1958).

44. C. E. Webster, R. S. Drago, M. C. Zerner, Molecular dimensions for adsorptives. *J. Am. Chem. Soc.* **120**, 5509–5516 (1998).

45. T. Kundu, B. B. Shah, L. Bolinois, D. Zhao, Functionalization-induced breathing control in metal-organic frameworks for methane storage with high deliverable capacity. *Chem. Mater.* **31**, 2842–2847 (2019).

46. M. Minceva, A. E. Rodrigues, Modeling and simulation of a simulated moving bed for the separation of p-xylene. *Ind. Eng. Chem. Res.* **41**, 3454–3461 (2002).

47. M. Minceva, A. E. Rodrigues, Influence of the transfer line dead volume on the performance of an industrial scale simulated moving bed for p-xylene separation. *Sep. Sci. Technol.* **38**, 1463–1497 (2003).

48. R. E. Dinnebier, A. Leineweber, J. S. O. Evans, *Rietveld Refinement: Practical Powder Diffraction Pattern Analysis Using TOPAS* (De Gruyter, 2019).

49. O. V. Dolomanov, L. J. Bourhis, R. J. Gildea, J. A. K. Howard, H. Puschmann, OLEX2: A complete structure solution, refinement and analysis program. *J. Appl. Cryst.* **42**, 339–341 (2009).

50. G. M. Sheldrick, SHELXT-integrated space-group and crystal-structure determination. *Acta Crystallogr. Sect. A Found. Adv.* **71**, 3–8 (2015).

51. G. M. Sheldrick, Crystal structure refinement with SHELXL. *Acta Crystallogr. Sect. C Struct. Chem.* **71**, 3–8 (2015).

52. W. Wan, J. Sun, J. Su, S. Hovmoller, X. Zou, Three-dimensional rotation electron diffraction: Software RED for automated data collection and data processing. *J. Appl. Cryst.* **46**, 1863–1873 (2013).

53. W. Kabsch, XDS. *Acta Crystallogr. Sect. D Biol. Crystallogr.* **66**, 125–132 (2010).

54. S. J. Clark, M. D. Segall, C. J. Pickard, P. J. Hasnip, M. I. J. Probert, K. Refson, M. C. Payne, First principles methods using CASTEP. *Z. Kristall. Kristallogr. Cryst. Mater.* **220**, 567–570 (2005).

55. J. P. Perdew, K. Burke, M. Ernzerhof, Generalized gradient approximation made simple. *Phys. Rev. Lett.* **77**, 3865–3868 (1997).

56. S. Grimme, Semiempirical GGA-type density functional constructed with a long-range dispersion correction. *J. Comput. Chem.* **27**, 1787–1799 (2006).

57. M. S. P. Silva, M. A. Moreira, A. F. P. Ferreira, J. C. Santos, V. M. T. M. Silva, P. S. Gomes, M. Minceva, J. P. B. Mota, A. E. Rodrigues, Adsorbent evaluation based on experimental

breakthrough curves: Separation of p-xylene from C8 isomers. *Chem. Eng. Technol.* **35**, 1777–1785 (2012).

58. M. S. P. Silva, J. P. B. Mota, A. E. Rodrigues, Fixed-bed adsorption of aromatic C-8 isomers: Breakthrough experiments, modeling and simulation. *Sep. Purif. Technol.* **90**, 246–256 (2012).

59. A. Kapoor, J. A. Ritter, R. T. Yang, An extended Langmuir model for adsorption of gas mixtures on heterogeneous surfaces. *Langmuir* **6**, 660–664 (1990).

60. M. Minceva, A. E. Rodrigues, Two-level optimization of an existing SMB for p-xylene separation. *Comput. Chem. Eng.* **29**, 2215–2228 (2005).

61. E. Santacesaria, M. Morbidelli, P. Danise, M. Mercenari, S. Carra, Separation of xylenes on Y zeolite. Part 1. Determination of the adsorption equilibrium parameters, selectivities and mass transfer coefficients through finite bath experiments. *Ind. Eng. Chem. Process Des. Dev.* **21**, 440–445 (1982).

62. A. Seidel-Morgenstern, H. Schmidt-Traub, M. Michel, A. Epping, A. Jupke, "Modeling and model parameters," in *Preparative Chromatography*, H. Schmidt-Traub, Ed. (Wiley-VCH Verlag GmbH & Co. KGaA, 2012), pp. 321–424.

63. A. E. Rodrigues, C. Pereira, M. Minceva, L. S. Pais, A. M. Ribeiro, A. Ribeiro, M. Silva, N. Gra, A. J. C. Santos, *Simulated Moving Bed Technology* (Elsevier Butterworth-Heinemann, ed. 1, 2015).

64. M. Zabka, M. Minceva, P. S. Gornes, A. E. Rodrigues, Chiral separation of R,S-alpha-tetralol by simulated moving bed. *Sep. Sci. Technol.* **43**, 727–765 (2008).

65. M. Mazzotti, G. Storti, M. Morbidelli, Optimal operation of simulated moving bed units for nonlinear chromatographic separations. *J. Chromatogr. A* **769**, 3–24 (1997).

66. M. Mazzotti, G. Storti, M. Morbidelli, Robust design of countercurrent adsorption separation processes: 2 Multicomponent systems. *AIChE J.* **40**, 1825–1842 (1994).

67. H. Renon, J. M. Prausnitz, Local compositions in thermodynamic excess functions for liquid mixtures. *Ind. Eng. Chem. Process Des. Dev.* **7**, 220–225 (1968).

68. M. Minceva, A. E. Rodrigues, Adsorption of xylenes on faujasite-type zeolite. *Chem. Eng. Res. Des.* **82**, 667–681 (2004).

69. H. Tournier, A. Barreau, B. Tavitian, D. Le Roux, J.-C. Moïse, J.-P. Bellat, C. Paulin, Adsorption equilibrium of xylene isomers and p-diethylbenzene on a prehydrated BaX zeolite. *Ind. Eng. Chem. Res.* **40**, 5983–5990 (2001).

70. M. Rasouli, N. Yaghobi, S. Z. M. Gilani, H. Atashi, M. Rasouli, Influence of monovalent alkaline metal cations on binder-free nano-zeolite X in para-xylene separation. *Chin. J. Chem. Eng.* **23**, 64–70 (2015).

71. Z. Gu, D. Jiang, H. Wang, X. Cui, X. Yan, Adsorption and separation of xylene isomers and ethylbenzene on two Zn-terephthalate metal-organic frameworks. *J. Phys. Chem. C* **114**, 311–316 (2010).

72. J. E. Warren, C. G. Perkins, K. E. Jelfs, P. Boldrin, P. A. Chater, G. J. Miller, T. D. Manning, M. E. Briggs, K. C. Stylianou, J. B. Claridge, M. J. Rosseinsky, Shape selectivity by guest-driven restructuring of a porous material. *Angew. Chem. Int. Ed. Engl.* **53**, 4592–4596 (2014).

73. J. Lannoeye, B. Van de Voorde, B. Bozbiyik, H. Reinsch, J. Denayer, D. De Vos, An aliphatic copper metal-organic framework as versatile shape selective adsorbent in liquid phase separations. *Micropor. Mesopor. Mater.* **226**, 292–298 (2016).

74. M. A. Moreira, M. P. S. Santos, C. G. Silva, J. M. Loureiro, J.-S. Chang, C. Serre, A. F. P. Ferreira, A. E. Rodrigues, Adsorption equilibrium of xylene isomers and ethylbenzene on MIL-125(Ti)-NH_2: The temperature influence on the para-selectivity. *Adsorption* **24**, 715–724 (2018).

75. S. I. Kim, S. Lee, Y. G. Chung, Y. S. Bae, The origin of p-xylene selectivity in a dabco pillar-layered metal-organic framework: A combined experimental and computational investigation. *ACS Appl. Mater. Interfaces* **11**, 31227–31236 (2019).

76. J. Lee, Y. Kim, Y. Son, H. Kim, Y. N. Choi, D. D'Alessandro, P. C. Rao, M. Yoon, Breathing-assisted selective adsorption of C_8 alkyl aromatics in Zn-based metal-organic frameworks. *Chem. A Eur. J.* **27**, 14851–14857 (2021).

77. H. J. Bae, S. I. Kim, Y. Choi, K. M. Kim, Y. S. Bae, High p-xylene selectivity in aluminum-based metal-organic framework with 1-D channels. *J. Ind. Eng. Chem.* **117**, 333–341 (2023).

78. J. A. Gee, K. Zhang, S. Bhattacharyya, J. Bentley, M. Rungta, J. S. Abichandani, D. S. Sholl, S. Nair, Computational identification and experimental evaluation of metal-organic frameworks for xylene enrichment. *J. Phys. Chem. C* **120**, 12075–12082 (2016).

79. J. Li, B. Sheng, R. Chen, H. Wu, W. Zhou, F. Zheng, B. Liu, Q. Yang, Z. Zhang, Y. Yang, Q. Ren, Z. Bao, An adaptive metal-organic framework discriminates xylene isomers by shape-responsive deformation. *Adv. Mater.* **37**, 2413506 (2025).

80. L. Yu, J. Zhang, S. Ullah, J. Yao, H. Luo, J. Huang, Q. Xia, T. Thonhauser, J. Li, H. Wang, Separating xylene isomers with a calcium metal-organic framework. *Angew. Chem. Int. Ed. Engl.* **62**, e202310672 (2023).

81. D. Peralta, G. Chaplais, J.-L. Paillaud, A. Simon-Masseron, K. Barthelet, G. D. Pirngruber, The separation of xylene isomers by ZIF-8: A demonstration of the extraordinary flexibility of the ZIF-8 framework. *Micropor. Mesopor. Mater.* **173**, 1–5 (2013).

82. J. M. Lin, C. T. He, P. Q. Liao, R. B. Lin, J. P. Zhang, Structural, energetic, and dynamic insights into the abnormal xylene separation behavior of hierarchical porous crystal. *Sci. Rep.* **5**, 11537 (2015).

83. G. Guo, H. Chen, Y. Long, Separation of p-xylene from C8 aromatics on binder-free hydrophobic adsorbent of MFI zeolite. I. Studies on static equilibrium. *Micropor. Mesopor. Mater.* **39**, 149–161 (2000).

84. A. Torres-Knoop, R. Krishna, D. Dubbeldam, Separating xylene isomers by commensurate stacking of p-xylene within channels of MAF-X8. *Angew. Chem. Int. Ed. Engl.* **53**, 7774–7778 (2014).

85. B. Saccoccia, A. M. Bohnsack, N. W. Waggoner, K. H. Cho, J. S. Lee, D.-Y. Hong, V. M. Lynch, J.-S. Chang, S. M. Humphrey, Separation of p-divinylbenzene by selective room-temperature adsorption inside Mg-CUK-1 prepared by aqueous microwave synthesis. *Angew. Chem. Int. Ed. Engl.* **54**, 5394–5398 (2015).

86. S. A. Mohamed, R. Zheng, N. Zhu, D. Zhao, J. Jiang, A systematic approach for incorporating structural flexibility in high-throughput computational screening of metal-organic frameworks for xylene separation. *J. Am. Chem. Soc.* **147**, 12251–12262 (2025).

87. Z. Qiao, Y. Yan, Y. Tang, H. Liang, J. Jiang, Metal-organic frameworks for xylene separation: From computational screening to machine learning. *J. Phys. Chem. C* **125**, 7839–7848 (2021).

88. P. Halder, J. K. Singh, Screening of hypothetical metal-organic frameworks for xylene isomers and ethylbenzene separation. *Energy Fuel* **37**, 2230–2236 (2023).
