# Supplementary material for: Separation of xylene isomers in an adaptable metal-organic framework
Source: Sci Adv. 2026 Aug 14;12(33):eaeg1691. doi: 10.1126/sciadv.aeg1691 (PMC13475607; doi:10.1126/sciadv.aeg1691)
Supplement: Supplementary file 2 [file sciadv.aeg1691_data_s1_and_s2.zip › aeg1691_data_s1.zip!/CIF/checkCIF-2266101.pdf]

checkCIF (basic structural check) running

checkCIF/PLATON (basic structural check)

You have not supplied any structure factors. As a result the full set of tests cannot be run.

No syntax errors found. CIF dictionary
Please wait while processing .... Interpreting this report

Structure factor report

Datablock: 23te03041

Bond precision:	C-C = 0.0136 A	Wavelength=0.02508

Cell:	a=20.690(4)	b=23.880(5)	c=7.8400(16)
	alpha=90	beta=90	gamma=90
Temperature: 293 K			

	Calculated	Reported
Volume	3873.6(14)	3873.6(13)
Space group	C 2 2 21	C 2 2 21
Hall group	C 2c 2	C 2c 2
Moiety formula	C16 H10 N4 Ni O4 [+ solvent]	C16 H10 N4 Ni O4
Sum formula	C16 H10 N4 Ni O4 [+ solvent]	C16 H10 N4 Ni O4
Mr	380.97	380.97
Dx,g cm-3	1.306	1.307
Z	8	8
Mu (mm-1)	0.000	0.000
F000	0.0	542.0
F000'	1551.60	
h,k,lmax		25,29,9
Nref		3814
Tmin,Tmax		
Tmin'		

Correction method= Not given

Data completeness= Theta(max)= 0.896

R(reflections)= 0.1871(2145) wR2(reflections)= 0.4504(3814)

S = 1.207 Npar= 70

The following ALERTS were generated. Each ALERT has the format
test-name_ALERT_alert-type_alert-level.
Click on the hyperlinks for more details of the test.

Alert level A

ATOM007_ALERT_1_A _atom_site_aniso_label is missing
Unique label identifying the atom site.

Author Response: All non-H atoms are modelled isotropically as their contribution to the scattering is expected for electron diffraction.

RINTA01_ALERT_3_A The value of Rint is greater than 0.25
Rint given 0.293
PLAT020_ALERT_3_A The Value of Rint is Greater Than 0.12 .... 0.293 Report

Author Response: A high Rint value is expected for merging multiple data.

PLAT201_ALERT_2_A Isotropic non-H Atoms in Main Residue(s) .... 26 Report
Ni01 Ni02 O004 O009 O00A etc.

Author Response: All non-H atoms are modelled isotropically as their contribution to the scattering is expected for electron diffraction.

Alert level B

PLAT082_ALERT_2_B High R1 Value .... 0.19 Report

Author Response: A high wR1 value is expected for electron diffraction.

PLAT084_ALERT_3_B High wR2 Value (i.e. > 0.25) .... 0.45 Report

Author Response: A high wR2 value is expected for electron diffraction.

Alert level C

PLAT241_ALERT_2_C High 'MainMol' Ueq as Compared to Neighbors of N03 Check
And 3 other PLAT241 Alerts
More ...
PLAT242_ALERT_2_C Low 'MainMol' Ueq as Compared to Neighbors of C006 Check
And 2 other PLAT242 Alerts
More ...
PLAT341_ALERT_3_C Low Bond Precision on C-C Bonds .... 0.01364 Ang.
PLAT411_ALERT_2_C Short Inter H...H Contact H00H ..H00B . 2.00 Ang.
x,y,1+z = 1_556 Check
PLAT411_ALERT_2_C Short Inter H...H Contact H00J ..H00Q . 2.08 Ang.
x,y,-1+z = 1_554 Check
PLAT767_ALERT_4_C INS Embedded LIST 6 Instruction Should be LIST 4 Please Check

Alert level G

ABSMU01_ALERT_1_G Calculation of _exptl_absorpt_correction_mu
not performed for this radiation type.
PLAT004_ALERT_5_G Polymeric Structure Found with Maximum Dimension 3 Info
PLAT007_ALERT_5_G Number of Unrefined Donor-H Atoms .... 2 Report
PLAT072_ALERT_2_G SHELXL First Parameter in WGHT Unusually Large 0.20 Report
PLAT171_ALERT_4_G The CIF-Embedded .res File Contains EADP Records 2 Report
PLAT172_ALERT_4_G The CIF-Embedded .res File Contains DFIX Records 1 Report
PLAT180_ALERT_4_G Check Cell Rounding: # of Values Ending with 0 = 3 Note
PLAT199_ALERT_1_G Reported _cell_measurement_temperature ..... (K) 293 Check
PLAT200_ALERT_1_G Reported _diffrn_ambient_temperature ..... (K) 293 Check
PLAT606_ALERT_4_G Solvent Accessible VOID(S) in Structure .... ! Info
PLAT720_ALERT_4_G Number of Unusual/Non-Standard Labels .... 34 Note
PLAT794_ALERT_5_G Tentative Bond Valency for Ni01 (II) . 2.02 Info
PLAT794_ALERT_5_G Tentative Bond Valency for Ni02 (II) . 1.92 Info
PLAT804_ALERT_5_G Number of ARU-Code Packing Problem(s) in PLATON 6 Info
PLAT860_ALERT_3_G Number of Least-Squares Restraints .... 1 Note

4 **ALERT level A** = Most likely a serious problem - resolve or explain
2 **ALERT level B** = A potentially serious problem, consider carefully
11 **ALERT level C** = Check. Ensure it is not caused by an omission or oversight
15 **ALERT level G** = General information/check it is not something unexpected

4 ALERT type 1 CIF construction/syntax error, inconsistent or missing data
12 ALERT type 2 Indicator that the structure model may be wrong or deficient
5 ALERT type 3 Indicator that the structure quality may be low
6 ALERT type 4 Improvement, methodology, query or suggestion
5 ALERT type 5 Informative message, check

It is advisable to attempt to resolve as many as possible of the alerts in all categories. Often the minor alerts point to easily fixed oversights, errors and omissions in your CIF or refinement strategy, so attention to these fine details can be worthwhile. In order to resolve some of the more serious problems it may be necessary to carry out additional measurements or structure refinements. However, the purpose of your study may justify the reported deviations and the more serious of these should normally be commented upon in the discussion or experimental section of a paper or in the "special_details" fields of the CIF. checkCIF was carefully designed to identify outliers and unusual parameters, but every test has its limitations and alerts that are not important in a particular case may appear. Conversely, the absence of alerts does not guarantee there are no aspects of the results needing attention. It is up to the individual to critically assess their own results and, if necessary, seek expert advice.

Publication of your CIF in IUCr journals

A basic structural check has been run on your CIF. These basic checks will be run on all CIFs submitted for publication in IUCr journals (*Acta Crystallographica*, *Journal of Applied Crystallography*, *Journal of Synchrotron Radiation*); however, if you intend to submit to *Acta Crystallographica Section C* or *E* or *IUCrData*, you should make sure that full publication checks are run on the final version of your CIF prior to submission.

Publication of your CIF in other journals

Please refer to the *Notes for Authors* of the relevant journal for any special instructions relating to CIF submission.

PLATON version of 28/11/2022; check.def file version of 28/11/2022

Datablock 23te03041 - ellipsoid plot

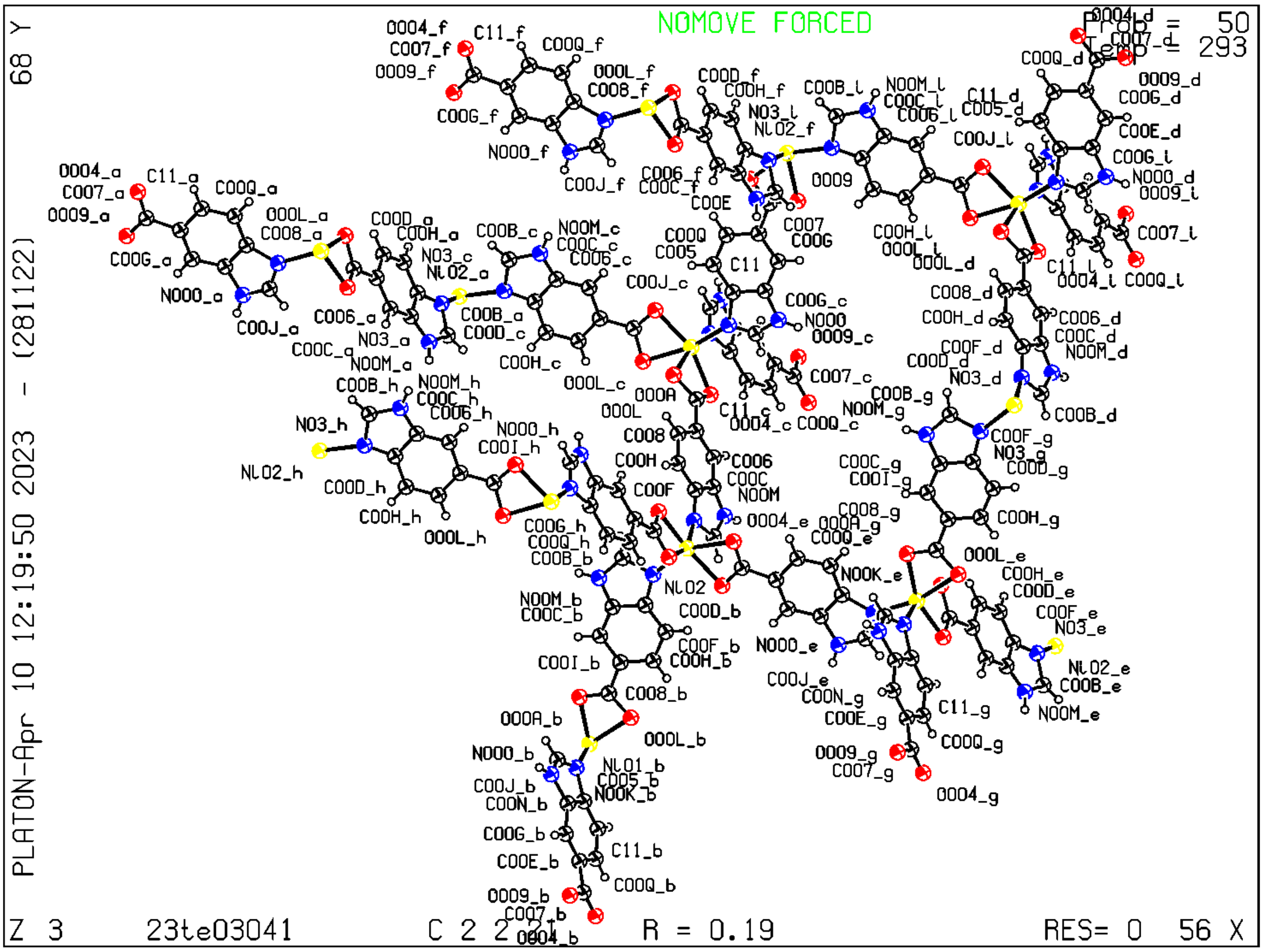

Download CIF editor (publCIF) from the IUCr
Download CIF editor (enCIFer) from the CCDC
Test a new CIF entry
