# Supplementary material for: Separation of xylene isomers in an adaptable metal-organic framework
Source: Sci Adv. 2026 Aug 14;12(33):eaeg1691. doi: 10.1126/sciadv.aeg1691 (PMC13475607; doi:10.1126/sciadv.aeg1691)

checkCIF/PLATON report

Structure factors have been supplied for datablock(s) 20200406zju_zjy_mx_0m_a_sq

THIS REPORT IS FOR GUIDANCE ONLY. IF USED AS PART OF A REVIEW PROCEDURE FOR PUBLICATION, IT SHOULD NOT REPLACE THE EXPERTISE OF AN EXPERIENCED CRYSTALLOGRAPHIC REFEREE.

No syntax errors found. CIF dictionary Interpreting this report

Datablock: 20200406zju_zjy_mx_0m_a_sq

Bond precision:	C-C = 0.0029 A		Wavelength=0.71073
Cell:	a=14.9659(7)	b=14.9659(7)	c=8.3925(4)
	alpha=90	beta=90	gamma=90
Temperature:	193 K		

	Calculated	Reported
Volume	1879.7(2)	1879.7(2)
Space group	P 43 21 2	P 43 21 2
Hall group	P 4nw 2abw	P 4nw 2abw
Moiety formula	C16 H10 N4 Ni O4 [+ solvent]	?
Sum formula	C16 H10 N4 Ni O4 [+ solvent]	C16 H10 N4 Ni O4
Mr	380.97	380.99
Dx,g cm-3	1.346	1.346
Z	4	4
Mu (mm-1)	1.057	1.057
F000	776.0	776.0
F000’	777.65	
h,k,lmax	17,17,9	17,17,9
Nref	1654[998]	1649
Tmin,Tmax	0.872,0.909	
Tmin’	0.872	

Correction method= Not given

Data completeness= 1.65/1.00 Theta(max)= 25.009

R(reflections)= 0.0208(1545) wR2(reflections)= 0.0597(1649)

S = 1.087 Npar= 115

```
The following ALERTS were generated. Each ALERT has the format
       test-name_ALERT_alert-type_alert-level.
Click on the hyperlinks for more details of the test.
```

Alert level C

```
STRVA01_ALERT_4_C          Flack test results are ambiguous.
           From the CIF: _refine_ls_abs_structure_Flack    0.490
           From the CIF: _refine_ls_abs_structure_Flack_su    0.030
PLAT052_ALERT_1_C Info on Absorption Correction Method  Not Given     Please Do !
PLAT250_ALERT_2_C Large U3/U1 Ratio for Average U(i,j) Tensor ....        2.5 Note
PLAT911_ALERT_3_C Missing FCF Refl Between Thmin & STh/L=    0.595          3 Report
```

Alert level G

```
PLAT004_ALERT_5_G Polymeric Structure Found with Maximum Dimension          3 Info
PLAT007_ALERT_5_G Number of Unrefined Donor-H Atoms .............           1 Report
PLAT033_ALERT_4_G Flack x Value Deviates > 3.0 * sigma from Zero .      0.490 Note
PLAT605_ALERT_4_G Largest Solvent Accessible VOID in the Structure        147 A**3
PLAT764_ALERT_4_G Overcomplete CIF Bond List Detected (Rep/Expd) .       1.11 Ratio
PLAT794_ALERT_5_G Tentative Bond Valency for Ni1       (II)      .       1.93 Info
PLAT869_ALERT_4_G ALERTS Related to the Use of SQUEEZE  Suppressed          ! Info
PLAT883_ALERT_1_G No Info/Value for _atom_sites_solution_primary .     Please Do !
PLAT899_ALERT_4_G SHELXL2018 is Deprecated and Succeeded by SHELXL     2019/3 Note
PLAT909_ALERT_3_G Percentage of I>2sig(I) Data at Theta(Max) Still        82% Note
PLAT910_ALERT_3_G Missing # of FCF Reflection(s) Below Theta(Min).          2 Note
PLAT913_ALERT_3_G Missing # of Very Strong Reflections in FCF ....          3 Note
PLAT965_ALERT_2_G The SHELXL WEIGHT Optimisation has not Converged     Please Check
PLAT967_ALERT_5_G Note: Two-Theta Cutoff Value in Embedded .res ..       50.0 Degree
PLAT978_ALERT_2_G Number C-C Bonds with Positive Residual Density.          3 Info
```

```
   0 ALERT level A = Most likely a serious problem - resolve or explain
   0 ALERT level B = A potentially serious problem, consider carefully
   4 ALERT level C = Check. Ensure it is not caused by an omission or oversight
  15 ALERT level G = General information/check it is not something unexpected

   2 ALERT type 1 CIF construction/syntax error, inconsistent or missing data
   3 ALERT type 2 Indicator that the structure model may be wrong or deficient
   4 ALERT type 3 Indicator that the structure quality may be low
   6 ALERT type 4 Improvement, methodology, query or suggestion
   4 ALERT type 5 Informative message, check
```

Validation response form

Please find below a validation response form (VRF) that can be filled in and pasted into your CIF.

```
# start Validation Reply Form
_vrf_STRVA01_20200406zju_zjy_mx_0m_a_sq
;
PROBLEM: Flack test results are ambiguous.
RESPONSE: ...
;
_vrf_PLAT052_20200406zju_zjy_mx_0m_a_sq
```

```
;
PROBLEM: Info on Absorption Correction Method   Not Given     Please Do !
RESPONSE: ...
;
_vrf_PLAT250_20200406zju_zjy_mx_0m_a_sq
;
PROBLEM: Large U3/U1 Ratio for Average U(i,j) Tensor ....        2.5 Note
RESPONSE: ...
;
_vrf_PLAT911_20200406zju_zjy_mx_0m_a_sq
;
PROBLEM: Missing FCF Refl Between Thmin & STh/L=    0.595          3 Report
RESPONSE: ...
;
# end Validation Reply Form
```

It is advisable to attempt to resolve as many as possible of the alerts in all categories. Often the minor alerts point to easily fixed oversights, errors and omissions in your CIF or refinement strategy, so attention to these fine details can be worthwhile. In order to resolve some of the more serious problems it may be necessary to carry out additional measurements or structure refinements. However, the purpose of your study may justify the reported deviations and the more serious of these should normally be commented upon in the discussion or experimental section of a paper or in the "special_details" fields of the CIF. checkCIF was carefully designed to identify outliers and unusual parameters, but every test has its limitations and alerts that are not important in a particular case may appear. Conversely, the absence of alerts does not guarantee there are no aspects of the results needing attention. It is up to the individual to critically assess their own results and, if necessary, seek expert advice.

Publication of your CIF in IUCr journals

A basic structural check has been run on your CIF. These basic checks will be run on all CIFs submitted for publication in IUCr journals (*Acta Crystallographica*, *Journal of Applied Crystallography*, *Journal of Synchrotron Radiation*); however, if you intend to submit to *Acta Crystallographica Section C* or *E* or *IUCrData*, you should make sure that full publication checks are run on the final version of your CIF prior to submission.

Publication of your CIF in other journals

Please refer to the *Notes for Authors* of the relevant journal for any special instructions relating to CIF submission.

PLATON version of 06/07/2023; check.def file version of 30/06/2023

Datablock 20200406zju_zjy_mx_0m_a_sq - ellipsoid plot

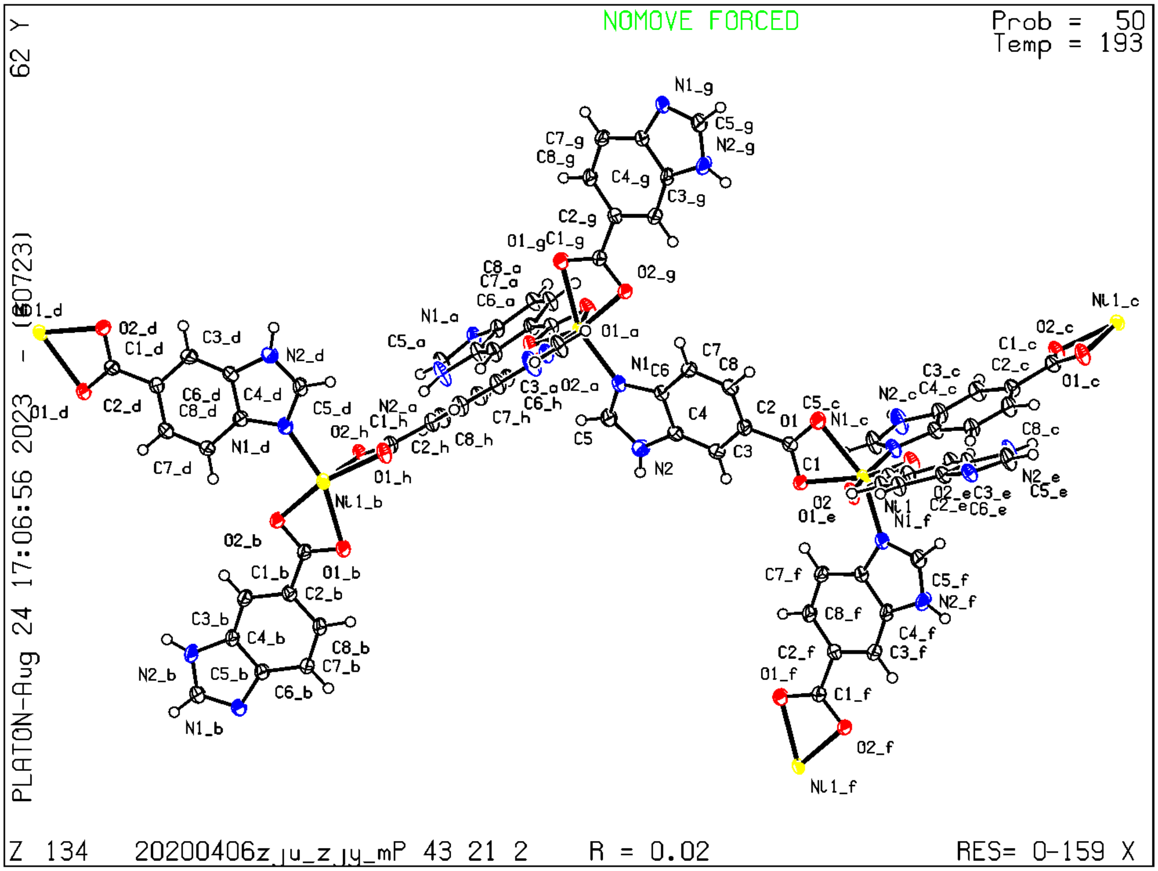

Supplement: Supplementary file 2 [file sciadv.aeg1691_data_s1_and_s2.zip › aeg1691_data_s1.zip!/CIF/checkCIF-2266103.pdf]
